# Designing narcissistic self-sorting terpyridine moieties with high coordination selectivity for complex metallo-supramolecules

**DOI:** 10.1038/s42004-021-00577-0

**Published:** 2021-09-24

**Authors:** Jianjun Ma, Tong Lu, Xiaozheng Duan, Yaping Xu, Zhikai Li, Kehuan Li, Junjuan Shi, Qixia Bai, Zhe Zhang, Xin-Qi Hao, Zhi Chen, Pingshan Wang, Ming Wang

**Affiliations:** 1 State Key Laboratory of Supramolecular Structure and Materials, College of Chemistry, Jilin University, Changchun, Jilin 130012 China; 2 State Key Laboratory of Polymer Physics and Chemistry, Changchun Institute of Applied Chemistry, Chinese Academy of Sciences, Changchun, Jilin 130022 China; 3 College of Chemistry and Environmental Engineering, Shenzhen University, Shenzhen, Guangdong 518060 China; 4 Institute of Environmental Research at Greater Bay Area; Key Laboratory for Water Quality and Conservation of the Pearl River Delta, Ministry of Education; Guangzhou Key Laboratory for Clean Energy and Materials; Guangzhou University, Guangzhou, Guangdong 510006 China; 5 Green Catalysis Center, Henan Key Laboratory of Chemical Biology and Organic Chemistry, and College of Chemistry, Zhengzhou University, Zhengzhou, Henan 450001 China

**Keywords:** Organometallic chemistry, Self-assembly

## Abstract

Coordination-driven self-assembly is a powerful approach for the construction of metallosupramolecules, but designing coordination moieties that can drive the self-assembly with high selectivity and specificity remains a challenge. Here we report two ortho-modified terpyridine ligands that form head-to-tail coordination complexes with Zn(II). Both complexes show narcissistic self-sorting behaviour. In addition, starting from these ligands, we obtain two sterically congested multitopic ligands and use them to construct more complex metallo-supramolecules hexagons. Because of the non-coaxial structural restrictions in the rotation of terpyridine moieties, these hexagonal macrocycles can hierarchically self-assemble into giant cyclic nanostructures via edge-to-edge stacking, rather than face-to-face stacking. Our design of dissymmetrical coordination moieties from congested coordination pairs show remarkable self-assembly selectivity and specificity.

## Abstract (web summary)

Coordination-driven self-assembly presents a key challenge of selectively targeting desired products. Here terpy-Zn complexes assemble head-to-tail, providing selective access to metallo-macrocycles

## Introduction

Coordination-driven self-assembly has been proven to be an effective approach for constructing supramolecular structures, due to its high predictability^1–6^. As such, a variety of discrete metallo-supramolecular architectures have been fabricated through different types of self-assembly strategies, including the coordinations of pyridyl^7^, bipyridyl^8^, terpyridyl^9^, or heterotopic ligands^10–12^ with naked metal ions or metal-organic components^13,14^. Given the increasing demands for diverse and predesigned molecular structures and functions, scientists are encouraged to further develop novel building blocks to achieve high coordination selectivity in precise and controllable supermolecular assembly.

2,2′:6′,2″-tpy and its derivatives have been widely employed in coordination-driven self-assembly on account of their variable coordination ability with transition metal ions, as well as the unique properties and wide applications in optical devices^15^, catalysis^16^, self-healing^17^, and drug delivery^18^ after complexation. Among the design of tpy-based metallo-supramolecules, the most common strategy is connecting tpy units and directing units at the central pyridine for subsequent coordination, which usually caused the poor selectivity of coordination units^4,19–23^. To further enhance geometrical diversity and complexity of assemblies, a hierarchical stepwise assembly strategy has been developed to promote the controllability of the self-assembly process by using metals that can form strong coordinative bonds with tpy, such as <tpy-Ru(II)-tpy> or <tpy-Fe(II)-tpy>^9,24,25^. However, the tedious synthesis and column separation procedures limit its extensive application in supermolecular constructions^25–27^. Currently, it still remains a challenge to precisely construct complex supramolecular structures through the one-step method.

**Fig. 1:**
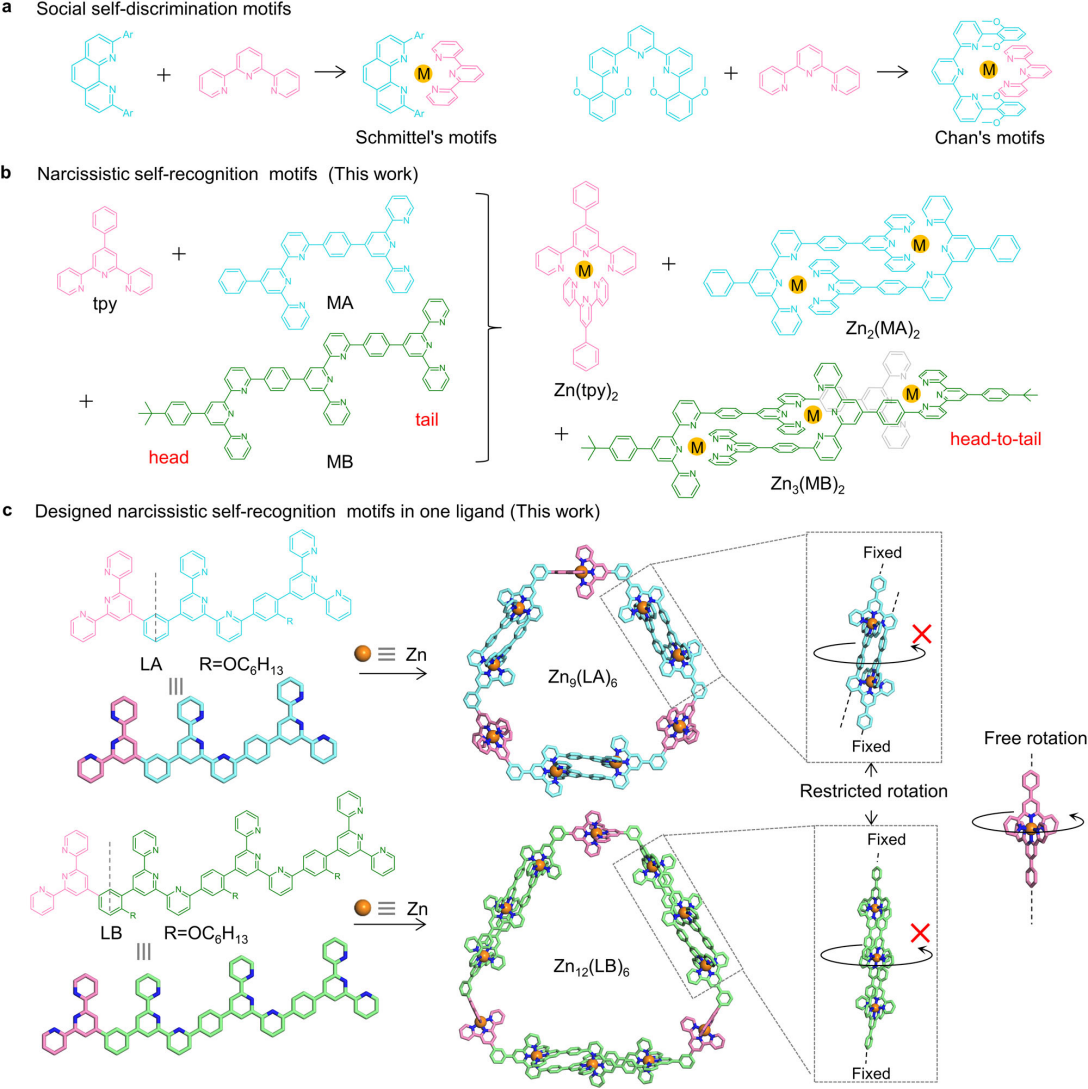
The self-assembly of metallo-macrocycles by the narcissistic self-recognition tpy moieties. Two different self-sorting processes in coordination-driven self-assembly; **a** social self-discrimination motifs in literatures; **b** narcissistic self-recognition motifs in the current work. We combined the narcissistic self-recognition tpy moieties with tpy to form multitopic ligands for precise self-assembly of metallo-macrocycles. **c** Schematics for the self-assembly of hexagonal macrocycles Zn_9_(LA)_6_ and Zn_12_(LB)_6_.

Nature has given us the enlightenment in the exploration of controllable self-assembly of supramolecules with the self-sorting process, such as the complementary pairing of DNA and the folding of polypeptides of proteins^28^. In recent years, much more attention has been paid to the design of different ligands for the diversity and controllability of supramolecular assembly through social self-discrimination or narcissistic self-recognition manner^29–33^. Particularly, social self-discrimination is relatively common for tpy moieties, and multiple different building blocks can be engineered to form impressively complex structures^34,35^. For example, phenanthroline with modification at 2,9-positions and tpy with modification at 6,6″-position could show the complete social self-sorting behaviors when mixed with tpy (Fig. 1a)^36,37^. In a recent review, Schmittel summarized a dynamic library formed by hetero-ligand motifs and introduced a lot of elegant structures constructed by the social self-sorting process^38^. In contrast, the narcissistic self-recognition manner for tpy moieties is rarely reported^39–41^. It has been more than 20 years since Lehn reported the first narcissistic self-sorting of a double helix, and there have also been many reports about narcissistic self-sorting in metal coordination^42–46^. However, most of these studies considering the narcissistic self-sorting of the structure as a whole, while the report focusing on narcissistic self-sorting used as self-recognition sites remains limited^45–51^. Furthermore, the homogeneous interactions in biological systems also prompted scientists to urgently develop ligand motifs for self-recognition^52,53^.

In this paper, to achieve coordination with high selectivity and specificity, we design two ortho-modified terpyridine ligands, i.e., MA and MB as model systems to form head-to-tail coordination complexes Zn_2_(MA)_2_ and Zn_3_(MB)_2_, respectively (Fig. 1b). The single-crystal structures of Zn_2_(MA)_2_ and Zn_3_(MB)_2_ show the non-coaxial feature of ortho-modified tpy after coordination, which is apparently different from the rotation of conventional tpy around its axis^19,54^. Remarkably, MA, MB, and tpy mixture with Zn(II) could assemble into three distinct complexes Zn_2_(MA)_2_, Zn_3_(MB)_2_, and Zn(tpy)_2_, suggesting the narcissistic self-sorting behaviors. We then combined these moieties with tpy to form sterically congested multitopic ligands (LA and LB) for precise self-assembly of hexagonal macrocycles Zn_9_(LA)_6_ and Zn_12_(LB)_6_ (Fig. 1c). It’s a common strategy to promote the hierarchical assembly of metallacycles by introducing orthogonal interactions^55–57^. The tpy-based metallacycles could hierarchically self-assembly into hollow tubular or berry-type nanostructure through the face-to-face stacking according to the previous reports^15,21,58,59^. Interestingly, it is found that the significantly steric congestion and non-coaxial structure of Zn_9_(LA)_6_ caused the restriction in the edging rotation of the hexagons, which further causes the hexagons to hierarchically assemble into giant cyclic nanostructures and metallogels via edge-to-edge stacking rather than face-to-face stacking.

## Results and discussion

### Synthesis and characterization of model system MA and complex Zn_2_(MA)_2_

In our design, the modification at terpyridyl 6-position played a central role in the self-soring assembly. The simple and efficient synthesis process of MA only included a three-step reaction of the starting material, followed by the one-step purification of the product using column chromatography. Especially, the key compound **1** was synthesized by a typical Kröhnke reaction with pyridinium salt **5** (Supplementary Fig. 1)^60^. The final motif MA was obtained via Suzuki coupling reaction in a good yield (77%) and characterized by NMR and mass spectrometry (Supplementary Figs. 2–8). After that, MA and Zn(NO_3_)_2_•6H_2_O (with a precise stoichiometric ratio of 1:1, Fig. 2a) were mixed in CHCl_3_ and MeOH (1:3, v/v) at 50 °C for 12 h, followed by the addition of excessive NH_4_PF_6_ (to exchange NO_3_^−^ with PF_6_^−^) in methanol to give a white precipitate Zn_2_(MA)_2_ in a yield of 91%.

**Fig. 2:**
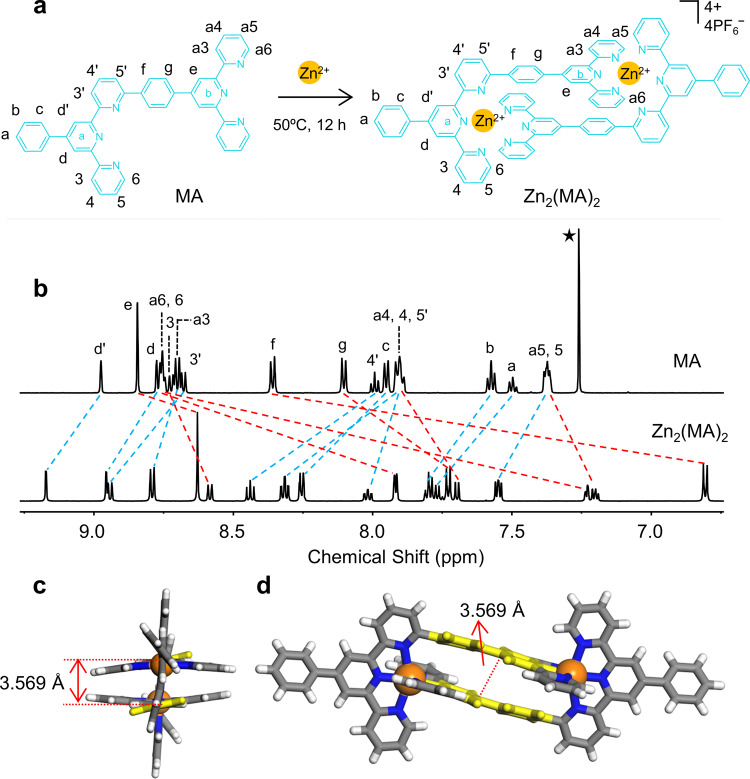
The self-assembly and characterization of head-to-tail complex Zn_2_(MA)_2_. **a** The self-assembly of model system MA with Zn(II). **b** The ^1^H NMR spectra (600 MHz, 300 K) of MA in CDCl_3_ and complex Zn_2_(MA)_2_ in CD_3_CN (3 mg/mL). **c** X-ray crystal structure in a side view of complex Zn_2_(MA)_2_. **d** X-ray crystal structure in a front view of complex Zn_2_(MA)_2_. Non-coordinated anions and solvent are omitted for clarity (C, gray or yellow; H, white; N, blue; Zn, orange).

^1^H NMR spectra of MA and complex Zn_2_(MA)_2_ were shown in Fig. 2b. Given the dissymmetrical nature of MA, five kinds of expected pyridines signals attributed to the terpyridine moieties were confirmed by 2D-COSY results (Supplementary Fig. 6). In the spectrum of complex Zn_2_(MA)_2_, the peaks were sharp and well-resolved. The 2D-COSY and NOESY for Zn_2_(MA)_2_ (Supplementary Figs. 9–14) also showed five sets of pyridines signals as MA, suggesting a highly symmetrical structure of complex Zn_2_(MA)_2_. Compared with MA, 6-position (6 and a6) signals of tpy were shifted upfield due to the electron shielding effect^61^. At the same time, the proton signals of e-tpy, f-Ph, and g-Ph were also shifted to upfield, which should be attributed to the existence of π–π interaction between tpy moiety and phenyl in the other ligand. In ESI-MS (Supplementary Fig. 15A), one prominent set of peaks with charge states from 2+ to 4+ was observed (due to the loss of different numbers of PF_6_^−^). After deconvolution, the molecular weight of complex Zn_2_(MA)_2_ was 1940 Da, matching well with its expected chemical composition of two MA moieties, two Zn(II) ions, and four PF_6_^−^. All the experimental isotope patterns agree excellently with the corresponding simulated isotope patterns (Supplementary Fig. 16). In traveling wave ion mobility-mass spectrometry (TWIM-MS)^23^, complex Zn_2_(MA)_2_ showed a series of charge states with narrow drift time distribution ranging from 2+ to 4+, indicating the formation of a discrete product but without any isomers and conformers (Supplementary Fig. 15B).

To further confirm the structure, the single crystal of Zn_2_(MA)_2_ was obtained by slowly diffusing the vapor of ethyl acetate into Zn_2_(MA)_2_ in acetonitrile for over 2 weeks. As expected, X-ray crystallographic analysis (Fig. 2c, d and Supplementary Table 1) showed that two Zn(II) are sandwiched between two MA to form a dimer with the head-to-tail structure (Supplementary Data 1 and Supplementary Movie 1). The phenyl groups in the middle are parallel to each other (yellow part), and the distance between the two interlayers is 3.57 Å, indicating the existence of π–π interactions^41,62^.

### Synthesis and characterization of model system MB and complex Zn_3_(MB)_2_

**Fig. 3:**
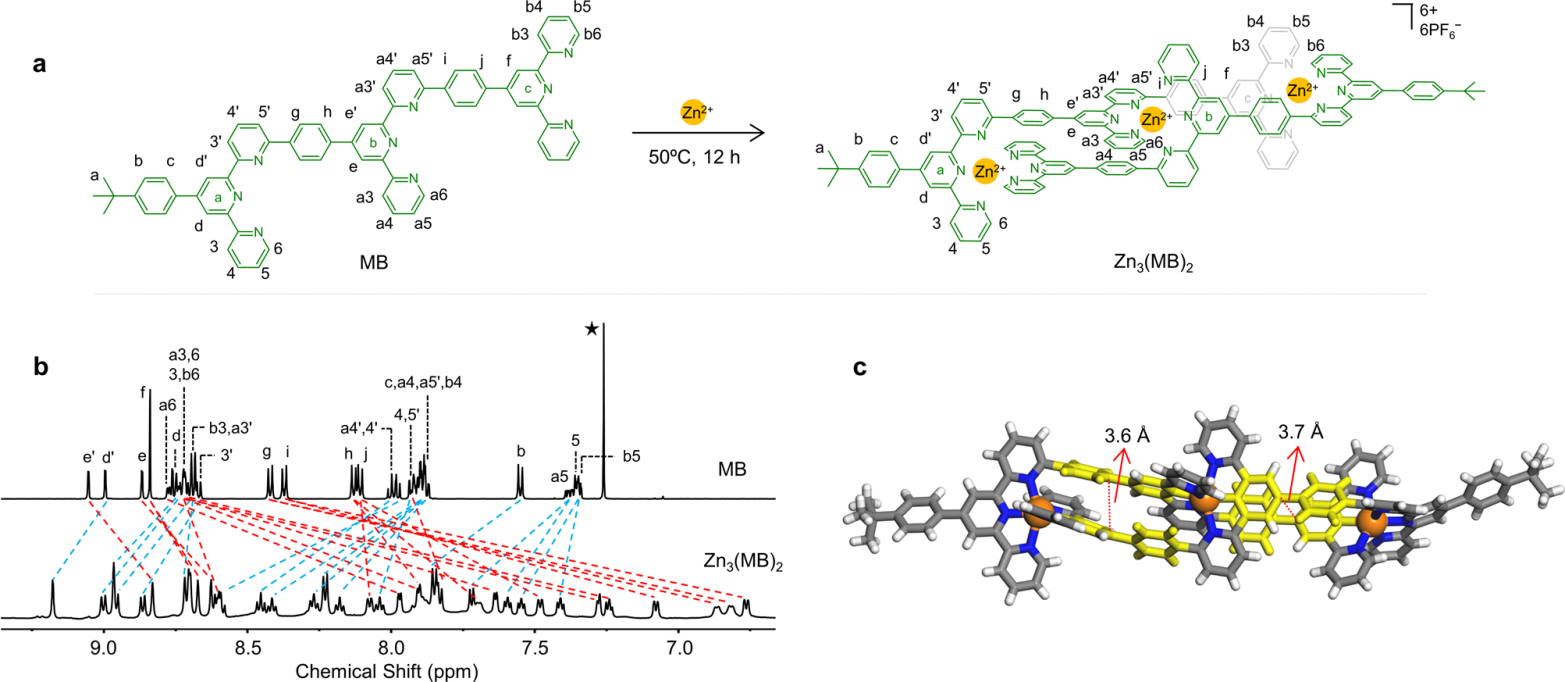
The self-assembly and characterization of head-to-tail complex Zn_3_(MB)_2_. **a** The self-assembly of model system MB with Zn(II). **b** The ^1^H NMR spectra (600 MHz, 300 K) of MB in CDCl_3_ and Zn_3_(MB)_2_ in CD_3_CN (3 mg/mL). **c** X-ray crystal structure in a front view of Zn_3_(MB)_2_. Non-coordinated anions and solvent are omitted for clarity (C, gray or yellow; H, white; N, blue; Zn, orange).

By referring to the synthetic and characterization procedures of MA, we further designed and obtain another model system MB (Fig. 3a and Supplementary Fig. 17), which includes one additional tpy unit compared to MA and thereby exhibits an enhanced sterically congested effect. An extra *tert*-butyl was introduced to improve the solubility. Tpy-based molecules have been extensively studied since the 1930s, however, it is worth noting that the unique structure of MB, has never been reported^40,63,64^. The synthesis of MB (yield 75%) and self-assembly of complex Zn_3_(MB)_2_ (yield 87%) (MB:Zn(II) = 2:3) followed the same procedure as that of MA and Zn_2_(MA)_2_.

The ^1^H NMR data of MB and Zn_3_(MB)_2_ were shown in Fig. 3b. In the spectrum of MB with three tpy units, eight kinds of pyridines signals could be observed (Supplementary Figs. 18–24). Moreover, the complex Zn_3_(MB)_2_ exhibited a more complicated structure and spectrum than Zn_2_(MA)_2_ (Supplementary Figs. 25–30), due to the enhanced steric hindrance effect caused by the introduction of the additional tpy unit. For each tpy, the differences in chemical shift (Δδ) were 1.48 ppm and 0.8 ppm for 6 position proton and a6 proton, respectively, suggesting that the 6-position of a-tpy shows the strong shielding effect. The signals of tpy-3 and tpy-5 displayed upfield or downfield shifts after coordination, which were consistent with the previous studies^65^. In addition, the restrictive structure induced the split of NMR signals. Both the signals of the middle phenyls and tpy-b6 split into two sets of peaks, which indicate their rotation restriction caused by the altered chemical environment. ESI-MS and TWIM-MS (Supplementary Figs. 31, 32) spectra of Zn_3_(MB)_2_ also exhibited a similar prominent set of peaks with different charge states and narrow drift time distribution ranging from 2+ to 4+, suggesting the formation of a single product without any overlapping isomers or conformers. The molecular weight of complex Zn_3_(MB)_2_ (3020 Da) agrees well with its expected chemical composition of two MB ligands, three Zn(II) ions, and six PF_6_^−^.

The single-crystal data of Zn_3_(MB)_2_ was obtained by slowly diffusing the vapor of carbon tetrachloride into acetonitrile solution for over 3 weeks. Complex Zn_3_(MB)_2_ also showed a sandwich-like structure with a certain degree of distortion, suggesting higher steric congestion caused the helical shape (Fig. 3c). The different distances between the two middle phenyls (yellow part) were 3.6 and 3.7 Å, respectively, which confirmed the helical structure (Supplementary Data 2, Supplementary Table 2, and Supplementary Movie 2). More importantly, under the sterically congested environment, the different tpy units coordinated with the same Zn(II) cannot maintain the vertical alignments and are forced to exhibit twisted structures. Furthermore, Zn_3_(MB)_2_ has two chiral conformations due to the unique head-to-tail coordination mode, and both of the conformations could be found in a single crystal (Supplementary Fig. 33).

### Self-sorting behavior of MA, MB, and tpy

**Fig. 4:**
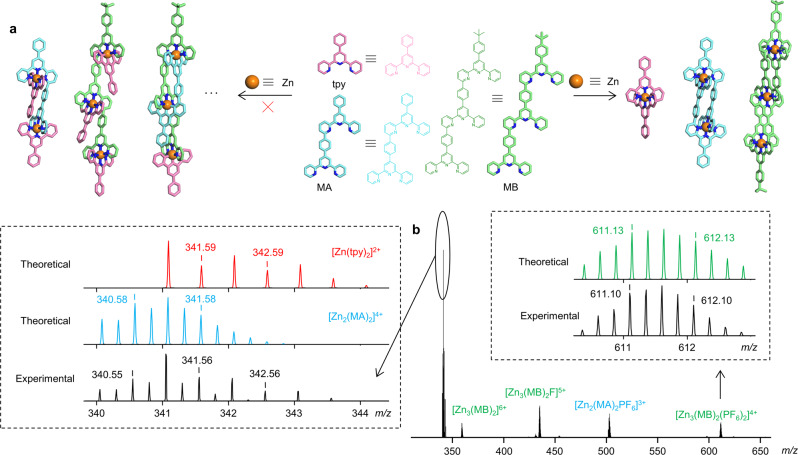
The narcissistic self-sorting behavior of MA, MB, and tpy. **a** A schematic representation for the narcissistic self-sorting process. **b** ESI-MS of the assembly of mixed MA, MB, and tpy in an equimolar ratio with the corresponding amount of Zn(II).

To evaluate the selectivity of these model systems, we investigated the self-sorting behavior of MA, MB, and conventional tpy^66^. In the self-sorting study, MA, MB, and conventional tpy were mixed together in an equimolar ratio with the corresponding amount of Zn(II) for the overnight self-assembly at 50 °C. ESI-MS clearly illustrated three sets of signals for the corresponding complexes but without statistical complexes (Fig. 4a, b), indicating that these model complexes exhibit ideal self-sorting properties in a pluralistic system. Both ESI-MS and NMR of any binary mixture (Supplementary Figs. 34–36) showed independent signals for three complexes Zn_2_(MA)_2_, Zn_3_(MB)_2_, and Zn(tpy)_2_, suggesting a characteristic narcissistic self-sorting^29,67,68^. Moreover, we also monitored the kinetic process of narcissistic self-sorting, and the system basically reached equilibrium after 24 h (Supplementary Fig. 37). As expectation, in accord to the maximum occupancy of coordination sites proposed by Lehn^46^, MA, MB, and tpy tend to narcissistically coordinate to form the most energy favorable structure. In this context, we are inspired to use MA and MB as self-recognition sites to further construct complex supramolecular structures.

### Synthesis and self-assembly of ligands LA and LB with multiple tpy moieties

**Fig. 5:**
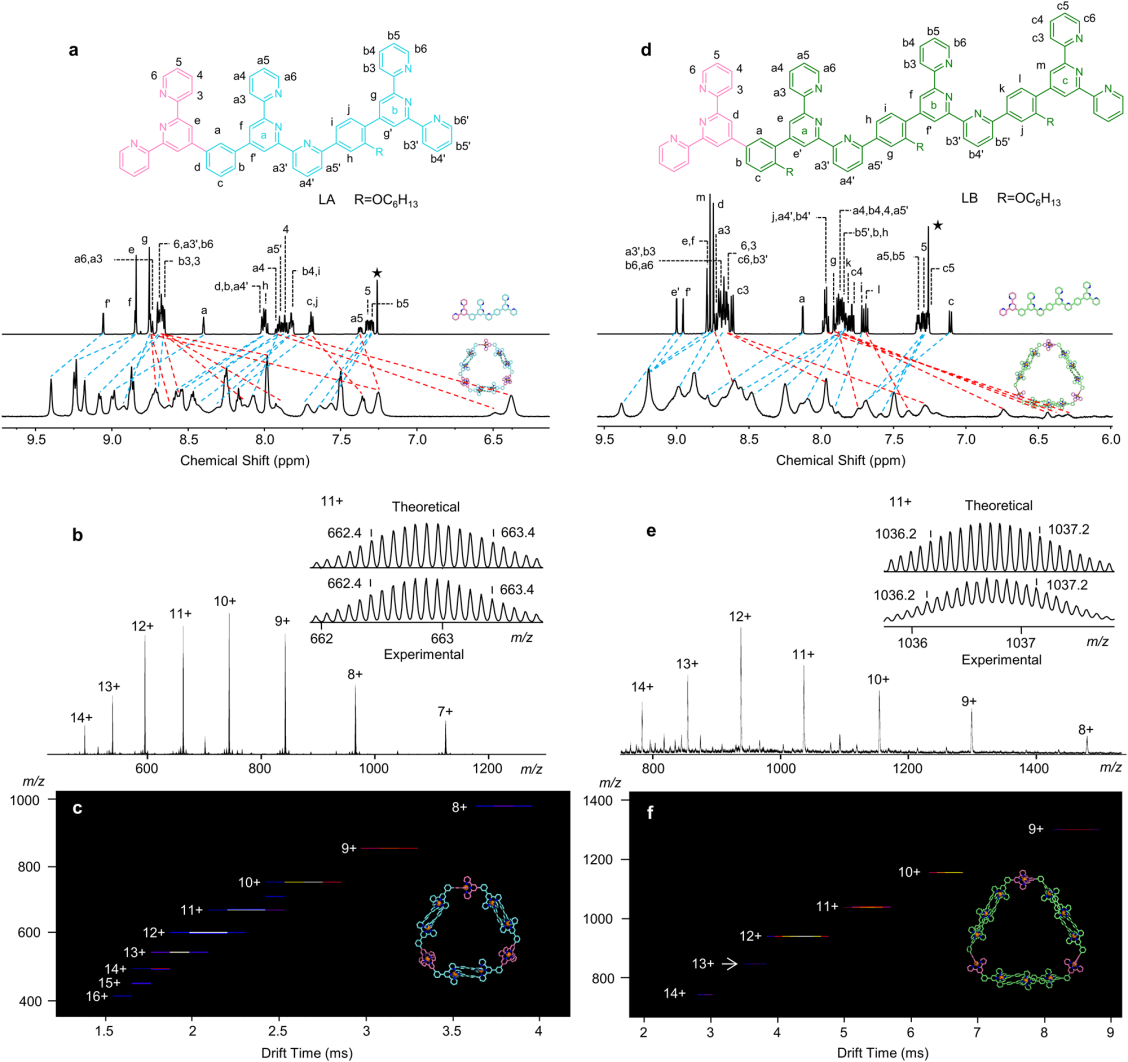
Characterization of the hexagonal macrocycles Zn_9_(LA)_6_ and Zn_12_(LB)_6_. **a** ^1^H NMR spectra (600 MHz, 300 K) of ligand LA in CDCl_3_ and hexagon Zn_9_(LA)_6_ in CD_3_CN (4 mg/mL). **b** ESI-MS, and **c** TWIM-MS plots (*m/z* vs drift time) of Zn_9_(LA)_6_. **d** ^1^H NMR spectra (600 MHz, 300 K) of ligand LB in CDCl_3_ and hexagon Zn_12_(LB)_6_ in CD_3_CN (4 mg/mL). **e** ESI-MS and **f** TWIM-MS plots (*m/z* vs drift time) of Zn_12_(LB)_6_.

It is reported that the ditopic tpy ligands with 120° angle tend to self-assemble into a mixture of macrocycles with uncontrollable size and structure^20^. However, we expect that the dissymmetrical tritopic (LA) and tetratopic (LB) (Fig. 1c) ligands with 120° angle that contain narcissistic self-sorting moieties could be used to precisely construct complex supramolecular macrocycles Zn_9_(LA)_6_ and Zn_12_(LB)_6_ (Supplementary Movie 3 and Supplementary Movie 4). In order to improve the solubility of ligands, alkoxy chains (R = -OC_6_H_13_) were introduced. Compared with LA (yield, 76%), the synthesis of LB with one more tpy units turns to be significantly challenging (Supplementary Fig. 38 and Supplementary Fig. 39). Fortunately, we successfully obtained LB (yield, 37%) by adding CH_3_COOH/DMF as an auxiliary agent. The obtained ligands LA and LB were assembled into complexes Zn_9_(LA)_6_ (yield, 86%) and Zn_12_(LB)_6_ (yield, 84%) and were characterized by NMR, MALDI-TOF, ESI-MS, and TWIM-MS (Fig. 5 and Supplementary Figs. 40–70).

Compared with LA and LB, ^1^H NMR spectra of the complexes Zn_9_(LA)_6_ and Zn_12_(LB)_6_ were significantly broadened (Fig. 5a, d), suggesting the assembly of large complexes with a slower tumbling on the NMR time scale^24^. Importantly, the peaks of pyridines on the b-tpy and c-tpy turn broader, due to the sterical congestion of the coordination units and the restriction in free rotation of the phenyl caused by the high steric congestion of alkoxy chains. In the spectrum of LA, seven kinds of expected pyridines signals were observed with respect to the tpy moieties. Owing to the continuous dissymmetrical modification of a- and b-tpy, LB displayed more complicated proton signals (i.e., ten distinguished kinds of pyridine peaks) than that of LA. All six positions of pyridines were shifted towards upfield, because of the electron shielding effects after complexation. However, the chemical shifts of different protons differ significantly owing to the variable shielding strength. For instance, the proton at a6 position showed a maximum chemical shift of Δδ = 1.41 ppm; while a slightly upfield-shift Δδ = 0.55 ppm was observed for b6 position. 2D-COSY and NOESY for two complexes Zn_9_(LA)_6_ and Zn_12_(LB)_6_ (Supplementary Figs. 53–56 and Supplementary Figs. 59–62) also showed the same sets of tpy signals as ligands, suggesting the symmetrical structures of these two complexes. The diffusion-ordered NMR spectroscopy (DOSY) provided dimensional information for Zn_9_(LA)_6_ and Zn_12_(LB)_6_. As shown in Supplementary Fig. 66, all proton signals appeared at the same band, indicating the formation of discrete assemblies. The diffusion coefficients in CD_3_CN were log D = −9.61 for Zn_9_(LA)_6_ and log D = −9.64 for Zn_12_(LB)_6_, respectively. The experimental hydrodynamic radii (rH) for Zn_9_(LA)_6_ (2.4 nm) and Zn_12_(LB)_6_ (2.6 nm) agreed well with the modeling structures.

In ESI-MS (Fig. 5b, e), one prominent set of peaks with charge states (from 7+ to 14+ for Zn_9_(LA)_6_ and from 8+ to 14+ for Zn_12_(LB)_6_) was observed on account of the loss of different numbers of PF_6_^−^. After deconvolution, the molecular weights of these two complexes were 8869 and 12974 Da, respectively, matching well with the expected chemical composition of Zn_9_(LA)_6_ (6 LA ligands, 9 Zn(II) ions, and 18 PF_6_^−^) and Zn_12_(LB)_6_ (6 LB ligands, 12 Zn(II) ions, and 24 PF_6_^−^). All the experimental isotope patterns agreed excellently with the corresponding simulated isotope patterns (Supplementary Figs. 69, 70). Moreover, the TWIM-MS spectra of complexes Zn_9_(LA)_6_ and Zn_12_(LB)_6_ also showed narrow drift time distribution, supporting the formation of single species with rigid structures (Fig. 5c, f). The full characterization of NMR, ESI-MS, and TWIM-MS confirmed that the two types of tpy moieties in ligands LA and LB could precisely self-assemble into hexagonal macrocycles through the narcissistic self-recognition mechanism. Such moieties with high coordination selectivity and specificity may find their potential in the constructions of more complex metallo-supramolecules with precisely controlled shapes and sizes.

Interestingly, when complexes Zn_9_(LA)_6_ and Zn_12_(LB)_6_ were mixed in an equimolar ratio at 50 °C for overnight, ESI-MS and TWIM-MS clearly illustrated two additional sets of signals besides Zn_9_(LA)_6_ and Zn_12_(LB)_6_ (Supplementary Fig. 71). The compositions of Zn_10_(LA)_4_(LB)_2_ and Zn_11_(LA)_2_(LB)_4_ were confirmed by analyzing the ESI-MS and TWIM-MS data (Supplementary Figs. 72,  73). No exchange for odd number ligands was observed in this mixture (Supplementary Figs. 74–77), suggesting that it could be a subcomponent exchange based on the dominant coordination dimer rather than the usual ligand exchange^69,70^. It indicated that the dimer structures based on ditopic and tritopic tpy moieties not only exhibit higher selectivity but also show higher stability than single-tpy unit.

### Hierarchical self-assembly

**Fig. 6:**
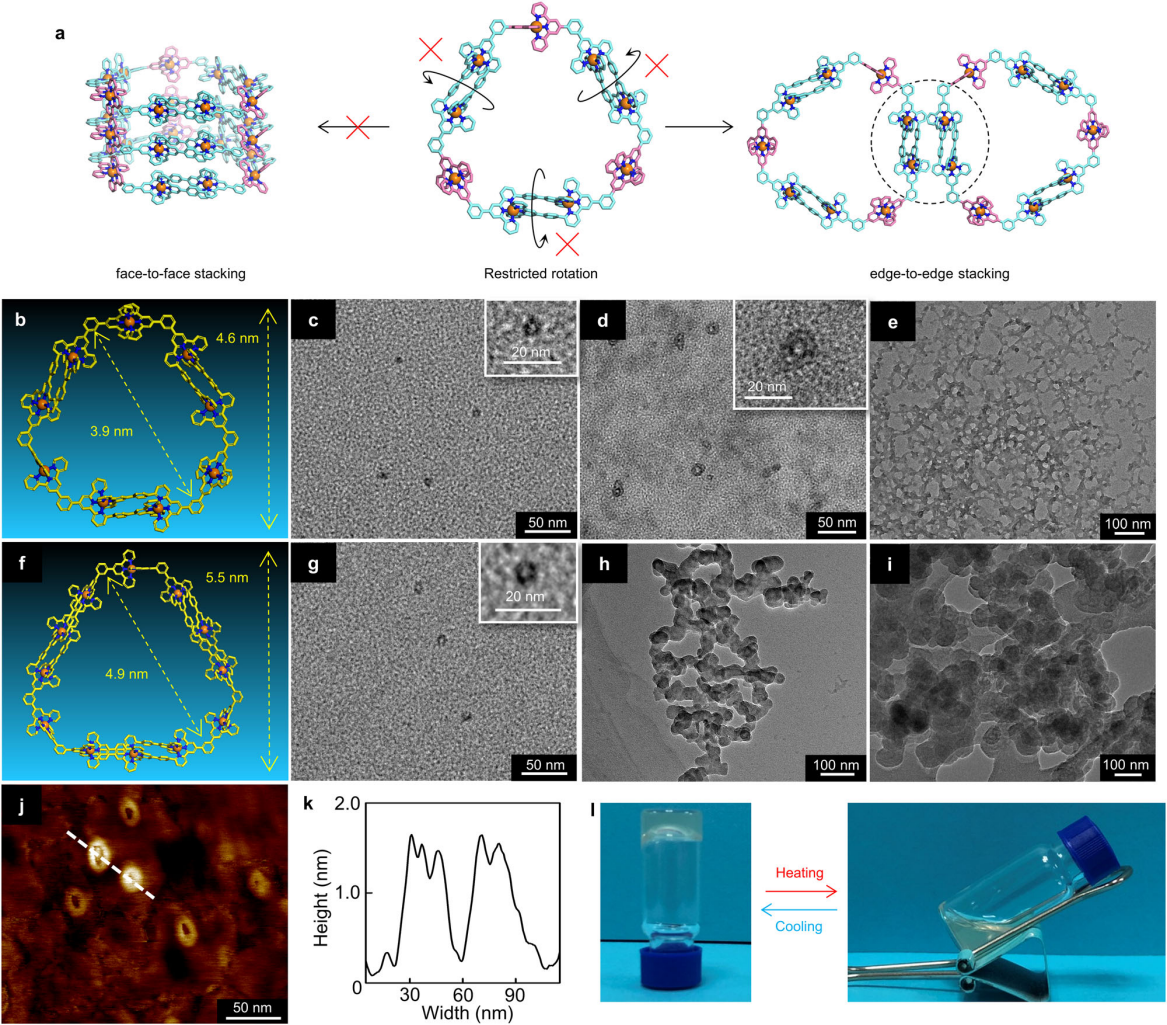
Characterization of the hierarchical self-assembly of Zn_9_(LA)_6_ and Zn_12_(LB)_6_. **a** Schematic diagram of edge-to-edge stacking by Zn_9_(LA)_6_. **b** The energy-minimized structure of Zn_9_(LA)_6_ from molecular modeling. **c** TEM images of individual Zn_9_(LA)_6_ at a concentration of 10^−6^ M in CH_3_CN (scale bar, 50 and 20 nm for zoom-in image). **d** TEM images of the assembled cyclic nanostructures of Zn_9_(LA)_6_ at a concentration of 10^−5^ M in CH_3_CN (scale bar, 50 and 20 nm for zoom-in image). **e** TEM images of the 3D network structure formed by assembled cyclic nanostructures of Zn_9_(LA)_6_ at a concentration of 10^−4^ M in CH_3_CN (scale bar, 100 nm). **f** The energy-minimized structure of Zn_12_(LB)_6_ from molecular modeling. **g** TEM images of individual Zn_12_(LB)_6_ at a concentration of 10^−6^ M in CH_3_CN (scale bar, 50 and 20 nm for zoom-in image). **h** TEM images of the assembled cyclic nanostructures of Zn_12_(LB)_6_ at a concentration of 10^−5^ M in CH_3_CN (scale bar, 100 nm). **i** TEM images of the 3D network structure formed by assembled cyclic nanostructures of Zn_12_(LB)_6_ at a concentration of 10^−4^ M in CH_3_CN (scale bar, 100 nm). **j** AFM images of the hierarchically assembled cyclic nanostructures by Zn_9_(LA)_6_ (scale bar, 50 nm). **k** Cross-section of the cyclic nanostructure shown in image **j**. **l** Photograph of Zn_12_(LB)_6_ gel formation at a concentration of 25 mg/mL in CH_3_CN.

Our study shows that, with increasing the concentration of Zn_9_(LA)_6_ and Zn_12_(LB)_6_ in acetonitrile at room temperature, both of the solutions could form metallogels at a concentration of 45 mg/mL for Zn_9_(LA)_6_ and 25 mg/mL for Zn_12_(LB)_6_ (Fig. 6l). The gels were also temperature-responsive and could be converted into solutions after heating at 50 °C for 1 h. To explore the mechanism of gelation, we then used TEM to investigate the morphology of Zn_9_(LA)_6_ and Zn_12_(LB)_6_ at different concentrations (Supplementary Figs. 78,  79). At low concentrations (10^−6^ M), the assemblies were almost distributed in a monodispersed manner. Both complexes exhibited unique hollow structures, suggesting high rigidity, stability, and large inner space. The measured sizes of the two complexes were 5.5 nm for Zn_9_(LA)_6_ and 6.5 nm for Zn_12_(LB)_6_, respectively (Fig. 6b, c, f, g). However, at a high concentration (10^−5^ M), some giant cyclic nanostructures in larger size were observed (Fig. 6d). The diameters of these structures were much larger than that of individual Zn_9_(LA)_6_, implying the clustering of Zn_9_(LA)_6_. The height of the hierarchically assembled nanostructures measured by the AFM image (Fig. 6j, k and Supplementary Figs. 80–83) was about 1.5 nm, which was very close to the height of the monolayer. When the concentration further increased, these giant macrocycles formed a three-dimensional network via intermolecular interaction (Fig. 6e). For Zn_12_(LB)_6_ with a larger size, although similar monolayer nanostructures were observed (Fig. 6h), the degree of aggregation was significantly increased (Supplementary Figs. 84–86). Further, at a much higher concentration of 10^−4^ M, the formed giant macrocycles showed obvious vertical stacking in space (Fig. 6i).

**Fig. 7:**
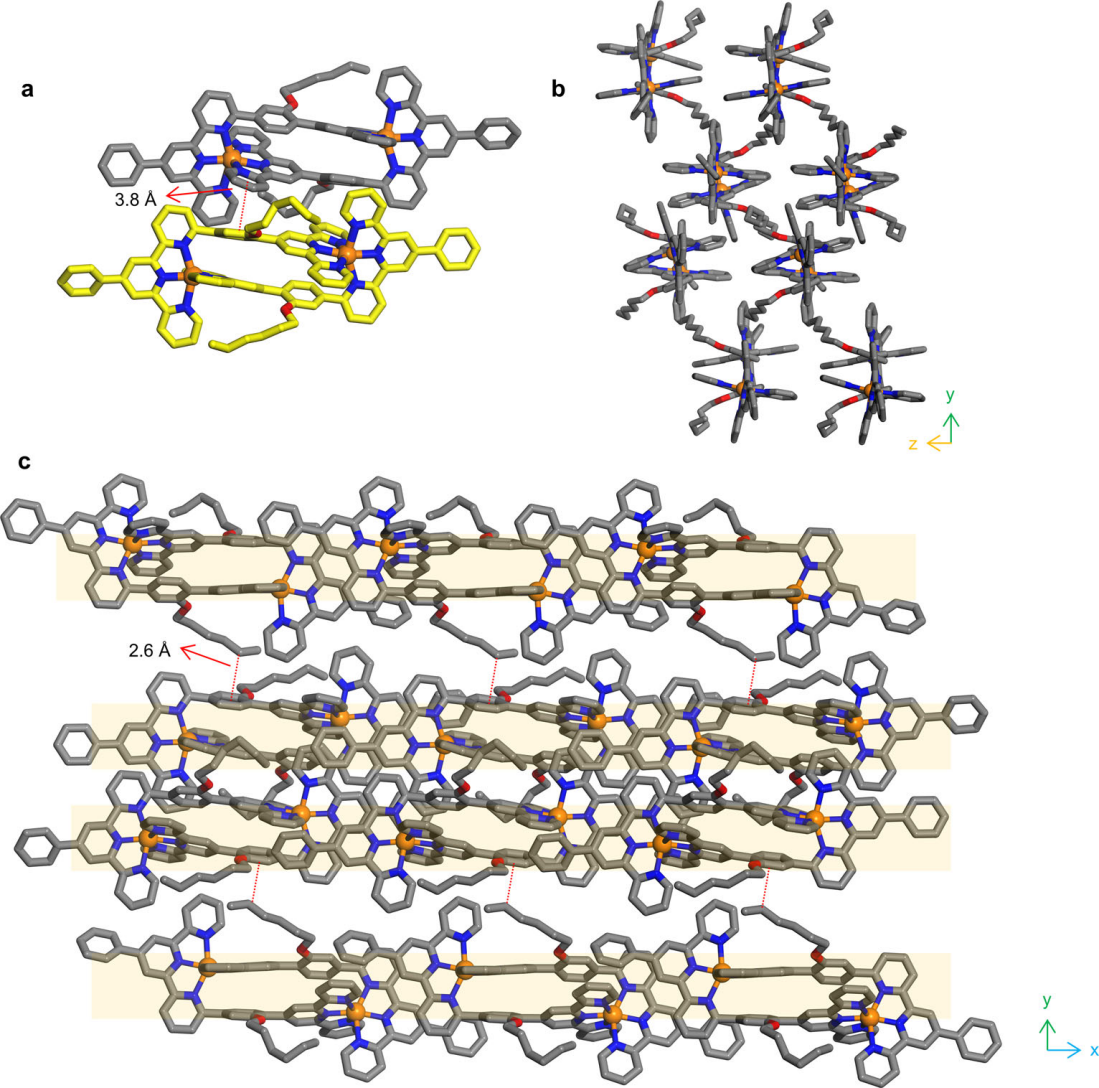
Crystallographic structure of complex Zn_2_(MA-OC_6_H_13_)_2_. **a** Crystal packing of two complexes Zn_2_(MA-OC_6_H_13_)_2_. **b** Crystal packing structure in a side view of complex Zn_2_(MA-OC_6_H_13_)_2_. **c** Crystal packing structure in a front view of complex Zn_2_(MA-OC_6_H_13_)_2_. H atoms, non-coordinated anions, and solvent are omitted for clarity (C, gray or yellow; N, blue; Zn, orange).

As well-known, the <tpy-M(II)-tpy> unit could rotate freely around the axis without space obstruction. However, due to the special spatially complementary coordination of LA and LB, these two axial dissymmetrical ligands exhibited entirely steric congestions. Therefore, it can be inferred that the hexagons are basically composed of vertical edges, since the rotation of the dimer can result in the disassembly (Fig. 6a). In order to study the edge-to-edge stacking of Zn_9_(LA)_6_, we tried to grow single crystal of Zn_9_(LA)_6_. However, all efforts to grow a single crystal of Zn_9_(LA)_6_ has proven to be unsuccessful. Instead, we synthesized a model ligand MA-OC_6_H_13_ (Supplementary Figs. 87–94) and further got the complex Zn_2_(MA-OC_6_H_13_)_2_ which has exactly the same structure as the ditopic part of Zn_9_(LA)_6_ (Supplementary Figs. 95–102). The single-crystal packing of Zn_2_(MA-OC_6_H_13_)_2_ provided strong evidence for the edge-to-edge stacking of Zn_9_(LA)_6_ (Supplementary Data 3 and Supplementary Table 3). As shown in Fig. 7, the intermolecular packing of Zn_2_(MA-OC_6_H_13_)_2_ is more compact than that of Zn_2_(MA)_2_ (Supplementary Fig. 103), which indicated that the introduction of alkyl chains can enhance the intermolecular interactions. The distance between pyridine and central phenyl of another molecule is 3.8 Å, which indicated the existence of π–π interaction. The distance between the alkyl chain and central phenyl of another molecule is 2.6 Å, which indicated the existence of CH–π interactions. Since Zn_12_(LB)_6_ could provide additional sites in the vertical direction than complex Zn_9_(LA)_6_, Zn_12_(LB)_6_ is hard to find the discrete cyclic nanostructures as that of Zn_9_(LA)_6_.

### Photophysical properties

**Fig. 8:**
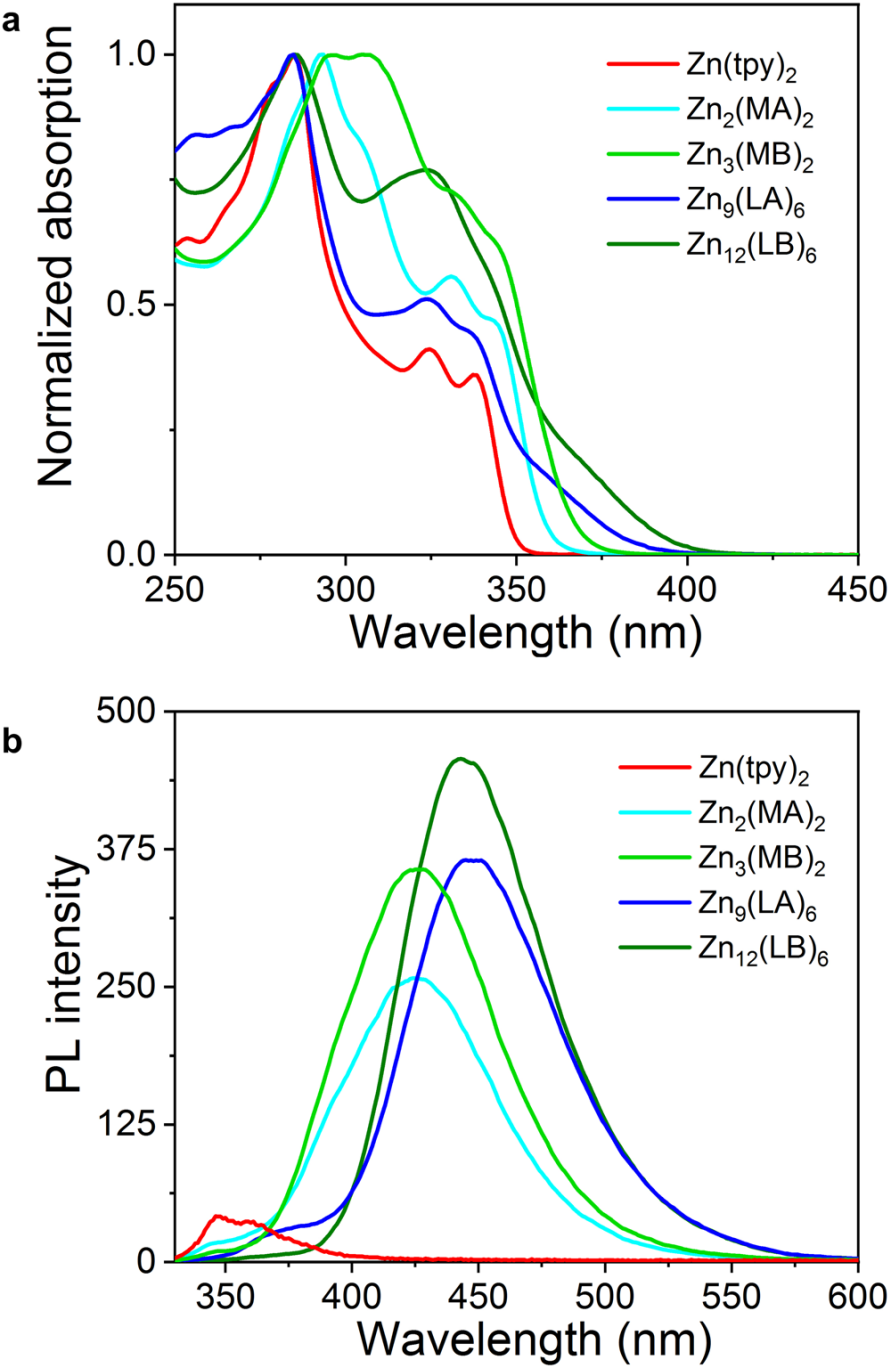
Photophysical data of the complexes. **a** Normalized UV/Vis absorption of all complexes in CH_3_CN. **b** PL spectra of all complexes in CH_3_CN (10^−6^ M, λ_e_ = 325 nm).

Considering the enhanced conjugation and restricted rotation of the structures, we further investigated UV/Vis absorption and photoluminescence of all ligands and complexes in the solution state (10^−6^ M). As shown in Fig. 8a, compared with the absorption spectra of ligands (Supplementary Figs. 104, 105), all complexes displayed a significant redshift originating from intra-ligand charge transfer (ILCT)^71^. In sharp contrast with Zn(tpy)_2,_ the maximum emission wavelength of Zn_2_(MA)_2_ displayed a ca. 75 nm redshift, ascribed to the enhanced conjugation (Fig. 8b). However, complex Zn_3_(MB)_2_ with a more complicated structure also showed a similar maximum emission wavelength to Zn_2_(MA)_2,_ the reason should be the twisted structures induced the conjugation. For hexagonal macrocycles Zn_9_(LA)_6_ and Zn_12_(LB)_6_, the restrictive intramolecular rotation in the congested and non-coaxial structures caused further enhance of their fluorescence intensity. Moreover, due to the introduction of alkoxy chains as electron donors, the maximum emission peaks of Zn_9_(LA)_6_ and Zn_12_(LB)_6_ exhibited 20 nm red-shifted than that of Zn_2_(MA)_2_ and Zn_3_(MB)_2_.

## Conclusion

In summary, we have designed and synthesized a series of multitopic tpy ligands with designed coordination selectivity by ortho-modification. As shown by the single-crystal detections, the model systems MA and MB can assemble into head-to-tail coordination complexes Zn_2_(MA)_2_ and Zn_3_(MB)_2_, respectively. Moreover, the assembly of mixtures of MA, MB, and tpy exhibit excellent selectivity due to the narcissistic self-sorting mechanism. Further, we introduced these moieties with high selectivity into multitopic ligands LA and LB, which were used to further precisely construct the hexagonal metallo-supramolecules. Such a design caused the restriction in rotations of the hexagons, which further leads to their hierarchical assembly into giant cyclic nanostructures and metallogels. Moreover, these complexes showed significantly enhanced fluorescence intensity in the solution state than the complexes based on conventional tpy due to the additional ligand conjugation and the restricted chemical environment. Our design and fabrication of dissymmetrical coordination moieties could pave a new avenue for the development of a set of congested coordination pairs with high selectivity and specificity for the assembly of sequence-specific metallo-supramolecular architectures.

## Methods

### General procedures

All reagents were purchased from Sigma-Aldrich, Matrix Scientific, Alfa Aesar, Jilin Chinese Academy of Sciences—Yanshen Technology Co. Ltd., and used without further purification. Column chromatography was conducted using SiO_2_ (VWR, 40–60 µm, 60 Å) and the separated products were visualized by UV light.

### Synthesis

All the new compounds were fully characterized and spectra are given in Supplementary Methods.

### Nuclear magnetic resonance (NMR)

NMR spectra data were recorded on a 400, 500, and 600 MHz Bruker Avance NMR spectrometer in CDCl_3_ or CD_3_CN with TMS as reference. For NMR spectra see Supplementary Information.

### ESI-MS and TWIM-MS

Electrospray ionization (ESI) mass spectra were recorded with a Waters Synapt G2 tandem mass spectrometer, using solutions of 0.5 mg sample in 1 mL of MeCN/MeOH (3:1, v/v) for complexes. The TWIM-MS experiments were performed under the following conditions: ESI capillary voltage, 3 kV; sample cone voltage, 30 V; extraction cone voltage, 3.5 V; source temperature 100 °C; desolvation temperature, 100 °C; cone gas flow, 10 L/h; desolvation gas flow, 700 L/h (N_2_); source gas control, 0 mL/min; trap gas control, 2 mL/min; helium cell gas control, 100 mL/min; ion mobility (IM) cell gas control, 30 mL/min; sample flow rate, 5 μL/min; IM traveling wave height, 25 V; and IM traveling wave velocity, 1000 m/s.

### Single-crystal X-ray diffractions

X-ray diffraction data for Zn_2_(MA)_2_ and Zn_2_(MA-OC_6_H_13_)_2_ were measured by a Bruker D8 Venture X-ray single-crystal diffractometer using a Cu·Kα radiation (λ = 1.54178 Å) at 100 K. X-ray diffraction data for Zn_3_(MB)_2_ was collected using synchrotron radiation and MAR325 CCD detector at Shanghai Synchrotron Radiation BL17B Beamline. Crystallographic data and structural characteristics of Zn_2_(MA)_2_, Zn_2_(MA-OC_6_H_13_)_2_, and Zn_3_(MB)_2_ are summarized in Supplementary Tables 1–3, respectively. Crystallographic information files for the complexes are provided in Supplementary Data 1–3, respectively.

### Transmission electron microscopy (TEM) analysis

The sample of Zn_9_(LA)_6_ and Zn_12_(LB)_6_ were dissolved in CH_3_CN at concentrations of 10^−6^, 10^−5^, and 10^−4^ M, respectively. The solutions were left overnight at 298 K and then dropped cast onto copper grids (ultrathin carbon supported by a lacey carbon film on a 400 Mesh copper grid) and the extra solution was absorbed by filter paper to avoid aggregation. The TEM images of the drop cast samples were taken with a JEM-2100F transmission electron microscope.

### AFM imaging

AFM imaging was performed on a Bruker Dimension Icon AFM system with ScanAsyst and the data was processed by NanoScope Analysis version 2.0 (Bruker Software, Inc.). The sample of Zn_9_(LA)_6_ and Zn_12_(LB)_6_ were dissolved in CH_3_CN at a concentration of 10^−5^ M, The solution was left overnight and then dropped cast onto a silicon wafer after surface cleaning.

### Photophysical measurements

UV-vis spectra of solutions were recorded on a PerkinElmer LAMBDA-365 spectrophotometer. Fluorescence emission spectra were measured by a Shimadzu spectrofluorimeter RF-5301PC. Solutions were placed in 1 cm path length quartz cells.

### Molecular modeling

Energy-minimized structures were obtained following the settings in the literature. Calculations were proceeded with Geometry Optimization and followed by Anneal in Forcite module of Materials Studio version 8.0 program (Accelrys Software, Inc.).

## Supplementary information

Description of Additional Supplementary Files

Supplementary Information

Peer Review File

Supplementary Data 1

Supplementary Data 2

Supplementary Data 3

Supplementary Movie 1

Supplementary Movie 2

Supplementary Movie 3

Supplementary Movie 4

## Data Availability

The authors declare that all data supporting the findings of this study are available within the article and Supplementary Information files, and also from the corresponding author upon reasonable request. The X-ray crystallographic coordinates for Zn_2_(MA)_2_ (Supplementary Data 1), Zn_3_(MB)_2_ (Supplementary Data 2), and Zn_2_(MA-OC_6_H_13_)_2_ (Supplementary Data 3) have been deposited at the Cambridge Crystallographic Data Centre (CCDC), under deposition numbers CCDC 2050233, 2050080, and 2106067, respectively. These data can be obtained free of charge from The Cambridge Crystallographic Data Centre at www.ccdc.cam.ac.uk/data_request/cif.

## References

[1] Lehn J-M (2007). From supramolecular chemistry towards constitutional dynamic chemistry and adaptive chemistry. Chem. Soc. Rev..

[2] Chakrabarty R, Mukherjee PS, Stang PJ (2011). Supramolecular coordination: self-assembly of finite two- and three-dimensional ensembles. Chem. Rev..

[3] Cook TR, Stang PJ (2015). Recent developments in the preparation and chemistry of metallacycles and metallacages via coordination. Chem. Rev..

[4] Chakraborty S, Newkome GR (2018). Terpyridine-based metallosupramolecular constructs: tailored monomers to precise 2D-motifs and 3D-metallocages. Chem. Soc. Rev..

[5] Olenyuk B, Whiteford JA, Fechtenkötter A, Stang PJ (1999). Self-assembly of nanoscale cuboctahedra by coordination chemistry. Nature.

[6] Fujita D (2016). Self-assembly of tetravalent Goldberg polyhedra from 144 small components. Nature.

[7] Sun QF (2010). Self-assembled M24L48 polyhedra and their sharp structural switch upon subtle ligand variation. Science.

[8] Danon JJ (2017). Braiding a molecular knot with eight crossings. Science.

[9] Newkome GR (2006). Nanoassembly of a fractal polymer: a molecular “Sierpinski hexagonal gasket”. Science.

[10] Zhang Y-Y, Gao W-X, Lin L, Jin G-X (2017). Recent advances in the construction and applications of heterometallic macrocycles and cages. Coord. Chem. Rev..

[11] Goswami A, Schmittel M (2018). Heteroleptic copper phenanthroline complexes in motion: From stand-alone devices to multi-component machinery. Coord. Chem. Rev..

[12] Tai C-Y (2019). Facile synthesis of multicomponent heterobimetallic metallomacrocycles through selective metal–ligand coordination. Chem. Commun..

[13] Hiraoka, S., Kubota, Y. & Fujita, M. Self- and hetero-recognition in the guest-controlled assembly of Pd(ii)-linked cages from two different ligands. *Chem. Commun*. 1509–1510 (2000).

[14] Zhao L (2008). Self-selection in the self-assembly of isomeric supramolecular squares from unsymmetrical bis(4-pyridyl)acetylene ligands. J. Org. Chem..

[15] Yin GQ (2018). Self-assembly of emissive supramolecular rosettes with increasing complexity using multitopic terpyridine ligands. Nat. Commun..

[16] Wei C, He Y, Shi X, Song Z (2019). Terpyridine-metal complexes: applications in catalysis and supramolecular chemistry. Coord. Chem. Rev..

[17] Bode S (2013). Self-healing polymer coatings based on crosslinked metallosupramolecular copolymers. Adv. Mater..

[18] Eryazici I, Moorefield CN, Newkome GR (2008). Square-planar Pd(II), Pt(II), and Au(III) terpyridine complexes: their syntheses, physical properties, supramolecular constructs, and biomedical activities. Chem. Rev..

[19] Newkome GR, Moorefield CN (2015). From 1 -> 3 dendritic designs to fractal supramacromolecular constructs: understanding the pathway to the Sierpinski gasket. Chem. Soc. Rev..

[20] Li X (2011). Separation and characterization of metallosupramolecular libraries by ion mobility mass spectrometry. Anal. Chem..

[21] Shi J (2021). Self-assembly of metallo-supramolecules with dissymmetrical ligands and characterization by scanning tunneling microscopy. J. Am. Chem. Soc..

[22] Chan Y-T, Li X, Moorefield CN, Wesdemiotis C, Newkome GR (2011). Towards larger polygonal architectures: synthesis and characterization of iron(II)- and ruthenium(II)-bis(terpyridine) metallomacrocycles. Chemistry.

[23] Chan Y-T (2011). Design, synthesis, and traveling wave ion mobility mass spectrometry characterization of iron(II)- and ruthenium(II)-terpyridine metallomacrocycles. J. Am. Chem. Soc..

[24] Zhang Z (2020). Intra- and intermolecular self-assembly of a 20-nm-wide supramolecular hexagonal grid. Nat. Chem..

[25] Jiang Z (2017). Self-assembly of a supramolecular hexagram and a supramolecular pentagram. Nat. Commun..

[26] Fu J-H, Wang S-Y, Chen Y-S, Prusty S, Chan Y-T (2019). One-pot self-assembly of stellated metallosupramolecules from multivalent and complementary terpyridine-based ligands. J. Am. Chem. Soc..

[27] Jiang Z (2020). Precise self-assembly of molecular four- and six-pointed stars. Inorg. Chem..

[28] Andersen ES (2009). Self-assembly of a nanoscale DNA box with a controllable lid. Nature.

[29] Wu A, Isaacs L (2003). Self-sorting: the exception or the rule?. J. Am. Chem. Soc..

[30] Acharyya K, Mukherjee S, Mukherjee PS (2013). Molecular marriage through partner preferences in covalent cage formation and cage-to-cage transformation. J. Am. Chem. Soc..

[31] Jiménez A (2014). Selective encapsulation and sequential release of guests within a self-sorting mixture of three tetrahedral cages. Angew. Chem. Int. Ed..

[32] Yamanaka M, Yamada Y, Sei Y, Yamaguchi K, Kobayashi K (2006). Selective formation of a self-assembling homo or hetero cavitand cage via metal coordination based on thermodynamic or kinetic control. J. Am. Chem. Soc..

[33] Sun QF, Sato S, Fujita M (2014). An M_12_(L(1))_12_(L(2))_12_ cantellated tetrahedron: a case study on mixed-ligand self-assembly. Angew. Chem. Int. Ed. Engl..

[34] Mahata K, Saha ML, Schmittel M (2010). From an eight-component self-sorting algorithm to a trisheterometallic scalene triangle. J. Am. Chem. Soc..

[35] Wang S-C, Cheng K-Y, Fu J-H, Cheng Y-C, Chan Y-T (2020). Conformational regulation of multivalent terpyridine ligands for self-assembly of heteroleptic metallo-supramolecules. J. Am. Chem. Soc..

[36] De S, Mahata K, Schmittel M (2010). Metal-coordination-driven dynamic heteroleptic architectures. Chem. Soc. Rev..

[37] Wang S-Y (2016). Metallo-supramolecular self-assembly of a multicomponent ditrigon based on complementary terpyridine ligand pairing. J. Am. Chem. Soc..

[38] Goswami A, Saha S, Biswas PK, Schmittel M (2020). (Nano)mechanical motion triggered by metal coordination: from functional devices to networked multicomponent catalytic machinery. Chem. Rev..

[39] Swiegers GF, Malefetse TJ (2000). New self-assembled structural motifs in coordination chemistry. Chem. Rev..

[40] Hofmeier H, Schubert US (2004). Recent developments in the supramolecular chemistry of terpyridine-metal complexes. Chem. Soc. Rev..

[41] Constable EC (2007). 2,2’:6’,2”-terpyridines: from chemical obscurity to common supramolecular motifs. Chem. Soc. Rev..

[42] Pinalli R (2004). Cavitand-based nanoscale coordination cages. J. Am. Chem. Soc..

[43] Caulder DL, Raymond KN (1997). Superamolecular self-recognition and self-assembly in gallium(III) catecholamide triple helices. Angew. Chem. Int. Ed. Engl..

[44] Taylor PN, Anderson HL (1999). Cooperative self-assembly of double-strand conjugated porphyrin ladders. J. Am. Chem. Soc..

[45] Rizzuto FJ, Nitschke JR (2020). Narcissistic, integrative, and kinetic self-sorting within a system of coordination cages. J. Am. Chem. Soc..

[46] Kramer R, Lehn JM, Marquis-Rigault A (1993). Self-recognition in helicate self-assembly: spontaneous formation of helical metal complexes from mixtures of ligands and metal ions. Proc. Natl Acad. Sci. USA.

[47] Safont-Sempere MM, Fernández G, Würthner F (2011). Self-sorting phenomena in complex supramolecular systems. Chem. Rev..

[48] He Z, Jiang W, Schalley CA (2015). Integrative self-sorting: a versatile strategy for the construction of complex supramolecular architecture. Chem. Soc. Rev..

[49] Ghosh A, Schmittel M (2020). Using multiple self-sorting for switching functions in discrete multicomponent systems. Beilstein J. Org. Chem..

[50] Zheng Y-R, Yang H-B, Northrop BH, Ghosh K, Stang PJ (2008). Size selective self-sorting in coordination-driven self-assembly of finite ensembles. Inorg. Chem..

[51] Johnson AM (2015). Narcissistic self-sorting in self-assembled cages of rare earth metals and rigid ligands. Angew. Chem. Int. Ed..

[52] Rubinstein R (2015). Molecular logic of neuronal self-recognition through protocadherin domain interactions. Cell.

[53] Goodman Kerry M (2016). Structural basis of diverse homophilic recognition by clustered α- and β-protocadherins. Neuron.

[54] Wild A, Winter A, Schlutter F, Schubert US (2011). Advances in the field of pi-conjugated 2,2’:6’,2”-terpyridines. Chem. Soc. Rev..

[55] Datta S, Saha ML, Stang PJ (2018). Hierarchical assemblies of supramolecular coordination complexes. Acc. Chem. Res..

[56] Sun Y, Chen C, Stang PJ (2019). Soft materials with diverse suprastructures via the self-assembly of metal-organic complexes. Acc. Chem. Res..

[57] Sepehrpour H, Fu W, Sun Y, Stang PJ (2019). Biomedically relevant self-assembled metallacycles and metallacages. J. Am. Chem. Soc..

[58] Song B (2018). Self-assembly of polycyclic supramolecules using linear metal-organic ligands. Nat. Commun..

[59] Chen M (2018). Highly stable spherical metallo-capsule from a branched hexapodal terpyridine and its self-assembled berry-type nanostructure. J. Am. Chem. Soc..

[60] KrÖHnke F (1976). The specific synthesis of pyridines and oligopyridines. Synthesis.

[61] Wang JL (2011). Stoichiometric self-assembly of shape-persistent 2D complexes: a facile route to a symmetric supramacromolecular spoked wheel. J. Am. Chem. Soc.,.

[62] Grimme S (2008). Do special noncovalent π–π stacking interactions really exist?. Angew. Chem. Int. Ed. Engl..

[63] Cargill Thompson AMW. (1997). The synthesis of 2,2′:6′,2″-terpyridine ligands — versatile building blocks for supramolecular chemistry. Coord. Chem. Rev..

[64] Housecroft CE, Constable EC (2020). The terpyridine isomer game: from chelate to coordination network building block. Chem. Commun..

[65] Wang M (2014). Hexagon wreaths: self-assembly of discrete supramolecular fractal architectures using multitopic terpyridine ligands. J. Am. Chem. Soc..

[66] Heller M. & Schubert, U. S. Syntheses of functionalized 2,2′:6′,2′′-terpyridines. *Eur. J. Org. Chem.* **2003**, 947–961 (2003).

[67] Lal Saha M, Schmittel M (2012). Degree of molecular self-sorting in multicomponent systems. Org. Biomol. Chem..

[68] Wang M (2014). From trigonal bipyramidal to platonic solids: self-assembly and self-sorting study of terpyridine-based 3D architectures. J. Am. Chem. Soc..

[69] Sato S, Ishido Y, Fujita M (2009). Remarkable stabilization of M_12_L_24_ spherical frameworks through the cooperation of 48 Pd(II)−pyridine interactions. J. Am. Chem. Soc..

[70] Wang L (2020). Introducing seven transition metal ions into terpyridine-based supramolecules: self-assembly and dynamic ligand exchange study. J. Am. Chem. Soc..

[71] Wang, X.-Y., Del Guerzo, A. & Schmehl, R. H. Preferential solvation of an ILCT excited state in bis(terpyridine–phenylene–vinylene) Zn(ii) complexes. *Chem. Commun*. **20**, 2344–2345 (2002). 10.1039/b205042k 12430432

