# Supplementary material for: Designing narcissistic self-sorting terpyridine moieties with high coordination selectivity for complex metallo-supramolecules
Source: Commun Chem. 2021 Sep 24;4:136. doi: 10.1038/s42004-021-00577-0 (PMC9814872; doi:10.1038/s42004-021-00577-0)
Supplement: Supplementary file 1 — Description of Additional Supplementary Files [file 42004_2021_577_MOESM1_ESM.pdf]

## Description of Additional Supplementary Files

**File Name:** Supplementary Data 1

**Description:** CIF file for $Zn_2(MA)_2$.

**File Name:** Supplementary Data 2

**Description:** CIF file for $Zn_3(MB)_2$.

**File Name:** Supplementary Data 3

**Description:** CIF file for $Zn_2(MA\text{-}OC_6H_{13})_2$.

**File Name:** Supplementary Movie 1

**Description:** The energy-minimized structure of $Zn_2(MA)_2$.

**File Name:** Supplementary Movie 2

**Description:** The energy-minimized structure of $Zn_3(MB)_2$.

**File Name:** Supplementary Movie 3

**Description:** The energy-minimized structure of $Zn_9(LA)_6$.

**File Name:** Supplementary Movie 4

**Description:** The energy-minimized structure of $Zn_{12}(LB)_6$.
