# Supplementary material for: Designing narcissistic self-sorting terpyridine moieties with high coordination selectivity for complex metallo-supramolecules
Source: Commun Chem. 2021 Sep 24;4:136. doi: 10.1038/s42004-021-00577-0 (PMC9814872; doi:10.1038/s42004-021-00577-0)
Supplement: Supplementary file 2 — Supplementary Information [file 42004_2021_577_MOESM2_ESM.pdf]

Designing Narcissistic Self-Sorting Terpyridine Moieties with High Coordination Selectivity for Complex Metallo-Supramolecules

Jianjun Ma[1], Tong Lu[1], Xiaozheng Duan[2], Yaping Xu[1], Zhikai Li[3], Kehuan Li[1], Junjuan Shi[1], Qixia Bai[4], Zhe Zhang[4], Xin-Qi Hao[5], Zhi Chen[3], Pingshan Wang[4], and Ming Wang[1,*]

[1] State Key Laboratory of Supramolecular Structure and Materials, College of Chemistry, Jilin University, Changchun, Jilin 130012, China

[2] State Key Laboratory of Polymer Physics and Chemistry, Changchun Institute of Applied Chemistry, Chinese Academy of Sciences, Changchun, Jilin 130022, China

[3] College of Chemistry and Environmental Engineering, Shenzhen University, Shenzhen, Guangdong 518060, China.

[4] Institute of Environmental Research at Greater Bay Area; Key Laboratory for Water Quality and Conservation of the Pearl River Delta, Ministry of Education; Guangzhou Key Laboratory for Clean Energy and Materials; Guangzhou University, Guangzhou, Guangdong 510006, China.

[5] Green Catalysis Center, Henan Key Laboratory of Chemical Biology and Organic Chemistry, and College of Chemistry, Zhengzhou University, Zhengzhou, Henan 450001, China.

Experimental Section

General Procedures. All reagents were purchased from Sigma-Aldrich, Matrix Scientific, Alfa Aesar, Jilin Chinese Academy of Sciences - Yanshen Technology Co. Ltd., and used without further purification. Column chromatography was conducted using SiO_2 (VWR, 40-60 μm, 60 Å) and the separated products were visualized by UV light.

Nuclear magnetic resonance (NMR). NMR spectra data were recorded on a 400 MHz, 500 MHz, and 600 MHz Bruker Avance NMR spectrometer in $CDCl_3$ or CD_3CN with TMS as reference.

Electrospray ionization-mass spectrometry (ESI-MS) and **travelling wave ion mobility-mass spectrometry (TWIM-MS)**. Electrospray ionization (ESI) mass spectra was recorded with a Waters Synapt G2 tandem mass spectrometer, using solutions of 0.5 mg sample in 1 mL of MeCN/MeOH (3:1, v/v) for complexes. The TWIM-MS experiments were performed under the following conditions: ESI capillary voltage, 3 kV; sample cone voltage, 30 V; extraction cone voltage, 3.5 V; source temperature 100 ºC; desolvation temperature, 100 ºC; cone gas flow, 10 L/h; desolvation gas flow, 700 L/h (N_2); source gas control, 0 mL/min; trap gas control, 2 mL/min; helium cell gas control, 100 mL/min; ion mobility (IM) cell gas control, 30 mL/min; sample flow rate, 5 μL/min; IM traveling wave height, 25 V; and IM traveling wave velocity, 1000 m/s.

Matrix-assisted laser desorption/ionization time-of-flight (MALDI-TOF) mass spectrometry. MALDI-TOF-MS was performed on a Bruker AutoflexIII using trans-2-[3-(4-*tert*-butylphenyl)-2-methyl-2-2-propenyli-dene]malononitrile (DCTB) as a matrix. The matrix dissolved in $CHCl_3$ at 20 mg mL^{-1} and organic compounds were dissolved in $CHCl_3$ at 10 mg mL^{-1}. Sample was prepared by depositing 0.5 μL of matrix on the wells of a 384-well ground-steel plate, allowing the spots to dry, depositing 0.5 μL of the sample on a spot of dry matrix, and adding another 0.5 μL of matrix on top of the dry sample. The plate was inserted into the MALDI source after drying. The sample was conducted in linear mode. And the data analysis was

conducted with Bruker's FlexAnalysis software.

TEM. The sample was dissolved in CH_3CN at concentrations of 10^{-6} M, 10^{-5} M and 10^{-4} M, respectively. The solutions were drop cast on to a copper grids (ultrathin carbon supported by a lacey carbon film on a 400 Mesh copper grid) and extra solution was absorbed by filter paper to avoid aggregation. The TEM images of the drop cast samples were taken with a JEM-2100F transmission electron microscope.

AFM. AFM imaging was performed on a Bruker Dimension Icon AFM system with ScanAsyst and the data was processed by NanoScope Analysis version 2.0 (Bruker Software, Inc.). The sample was dissolved in CH_3CN at a concentration of 10^{-5} M, and then dropping the diluted solution onto a silicon wafer after surface cleaning.

Single crystal X-ray diffractions. X-ray diffraction data for $Zn_2(MA)_2$ and $Zn_2(MA\text{-}OC_6H_{13})_2$ were measured by a Bruker D8 Venture X-ray single crystal diffractometer using a Cu·Kα radiation (λ = 1.54178 Å) at 100 K. X-ray diffraction data for $Zn_3(MB)_2$ was collected using synchrotron radiation and MAR325 CCD detector at Shanghai Synchrotron Radiation BL17B Beamline.

Photophysical measurements. UV-vis spectra of solutions were recorded on a PerkinElmer LAMBDA-365 Spectrophotometer. Fluorescence emission spectra was measured by a Shimadzu spectrofluorimeter RF-5301PC. Solutions were placed in 1 cm path length quartz cells.

Molecular Modeling. Energy-minimized structures were obtained following the settings in the literature. Calculations were proceeded with Geometry Optimization and followed by Anneal in Forcite module of Materials Studio version 8.0 program (Accelrys Software, Inc.).

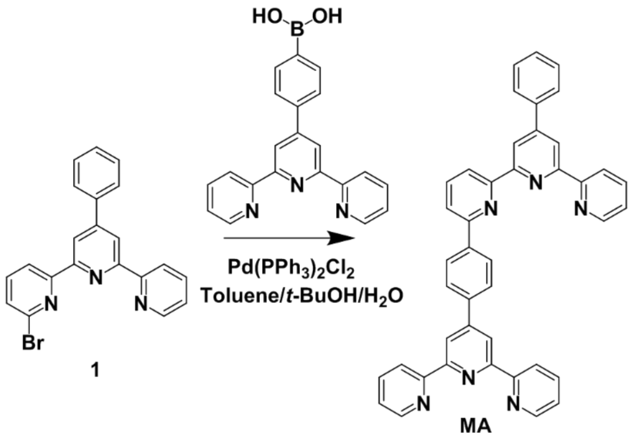

Supplementary Figure 1. Synthesis of ligand **MA**.

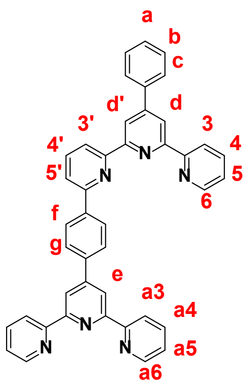

MA: A mixture of compound **1**[1] (775 mg, 2 mmol), 4-boronatophenyl terpyridine (1.06 g, 3 mmol) and $Pd(PPh_3)_2Cl_2$ (112 mg, 0.16 mmol) in 100 mL Schlenk flask was degassed three times. Then toluene (30 mL), 1 M Na_2CO_3 (15 mL) and *tert*-butyl alcohol (7 mL) were added under N_2. The resultant mixture was kept at 85 ^{o}C for 24 h. After cooling down to the room temperature, the solution was extracted three times with $CHCl_3$, and the solvent was removed under reduced pressure. The crude product was purified by column chromatography on silica gel ($CHCl_3$/EtOH=75/1) to give compound **MA** as white solid (950 mg, 77%). ^{1}H NMR (600 MHz, $CDCl_3$, 300 K) δ 8.97 (s, 1H, tpy-$H^{d'}$), 8.84 (s, 2H, tpy-H^{e}), 8.78 (m, 4H, - tpy-H^{d}, tpy-H^{a6} and tpy-H^{6}), 8.73 (d, 1H, J = 7.8 Hz, tpy-H^{3}), 8.71 (d, 2H, J = 7.8 Hz, tpy-H^{a3}), 8.69 (d, 1H, J = 7.8 Hz, tpy-$H^{3'}$), 8.38 (d, 2H, J = 7.8 Hz, Ph-H^{f}), 8.12 (d, 2H, J = 7.8 Hz, Ph-H^{g}), 8.02 (t, 1H, J = 7.8 Hz, tpy-$H^{4'}$), 7.98 (d, 2H, J = 7.8 Hz, Ph-H^{c}), 7.93 (m, 4H, tpy-H^{a4}, tpy-H^{4} and tpy-$H^{5'}$), 7.60 (t, 2H, J = 7.8 Hz, Ph-H^{b}), 7.52 (t, 1H, J = 7.2 Hz, Ph-H^{a}), 7.37 (m, 3H, tpy-H^{a5} and tpy-H^{5}). ^{13}C NMR (150 MHz, $CDCl_3$, 300 K) δ 156.50, 156.42,

156.18, 156.03, 155.89, 150.51, 149.88, 149.31, 140.11, 139.13, 138.91, 137.87, 137.06, 129.19, 127.89, 127.67, 127.53, 124.02, 121.56, 120.67, 120.10, 119.36, 119.24, 118.97. MALDI-TOF MS (*m/z*): Calcd. for $[C_{42}H_{28}N_6+H]^+$ 617.24. Found: 617.24.

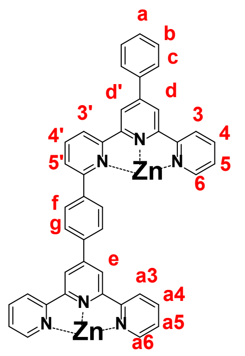

Complex $Zn_2(MA)_2$: To a solution of **MA** (4.3 mg, 6 μmol) in $CHCl_3$ (1.0 mL), a solution of $Zn(NO_3)_2$•$6H_2O$ (1.8 mg, 6 μmol) in MeOH (3.0 mL) was added, then the mixture was kept in a 50 °C for 12 h. After cooling to room temperature, excess NH_4PF_6 (around 80 mg) was added to generate a white precipitate (5.3 mg, 91%). ^{1}H NMR (600 MHz, CD_3CN, 300 K) δ 9.18 (d, 1H, *J* = 1.2 Hz, tpy-$H^{d'}$), 8.97 (d, 1H, *J* = 1.2 Hz, tpy-H^{d}), 8.94 (dd, 1H, *J* = 8.4, 0.6 Hz, tpy-$H^{3'}$), 8.81 (d, 2H, *J* = 7.8 Hz, tpy-H^{a3}), 8.64 (s, 2H, tpy-H^{e}), 8.59 (d, 1H, *J* = 8.4 Hz, tpy-H^{3}), 8.45 (t, 1H, *J* = 7.8 Hz, tpy-$H^{4'}$), 8.32 (dt, 2H, *J* = 7.8, 1.2 Hz, tpy-H^{a4}), 8.26 (m, 2H, Ph-H^{c}), 8.02 (dt, 1H, *J* = 7.8, 1.2 Hz, tpy-H^{4}), 7.93 (dd, 2H, *J* = 5.4, 0.6 Hz, tpy-H^{a6}), 7.83 - 7.75 (m, 3H, Ph-H^{b} and Ph-H^{a}), 7.73 (d, 2H, *J* = 7.8 Hz, Ph-H^{g}), 7.70 (dd, 1H, *J* = 7.8, 1.2 Hz, tpy-$H^{5'}$), 7.56 (ddd, 2H, *J* = 7.2, 5.4, 1.2 Hz, tpy-H^{a5}), 7.24 (m, 1H, tpy-H^{6}), 7.21 (m, 1H, tpy-H^{5}), 6.82 (d, 2H, *J* = 8.4 Hz, Ph-H^{f}). ^{13}C NMR (150 MHz, CD_3CN, 300 K) δ 193.99, 160.80, 157.38, 154.10, 152.04, 151.21, 150.53, 149.62, 149.31, 148.60, 147.82, 147.77, 142.50, 142.45, 141.80, 141.38, 137.27, 136.91, 132.27, 130.65, 129.53, 129.07, 128.92, 128.26, 127.85, 124.72, 124.09, 123.96, 123.05, 122.75, 121.58. ESI-MS (*m/z*): 1799.3 $[M-PF_6^-]^{1+}$ (calcd *m/z*: 1799.3), 827.2 $[M-2PF_6^-]^{2+}$ (calcd *m/z*: 827.2), 503.1 $[M-3PF_6^-]^{3+}$ (calcd *m/z*: 503.1), 341.1 $[M-4PF_6^-]^{4+}$ (calcd *m/z*: 341.1).

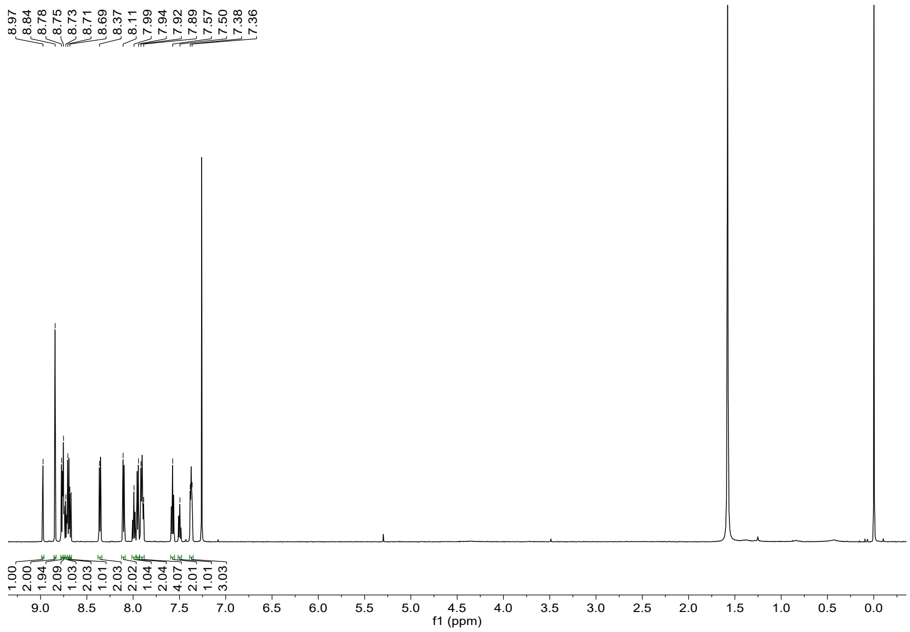

Supplementary Figure 2. ^{1}H NMR (600 MHz, $CDCl_3$, 300 K) spectrum of **MA**.

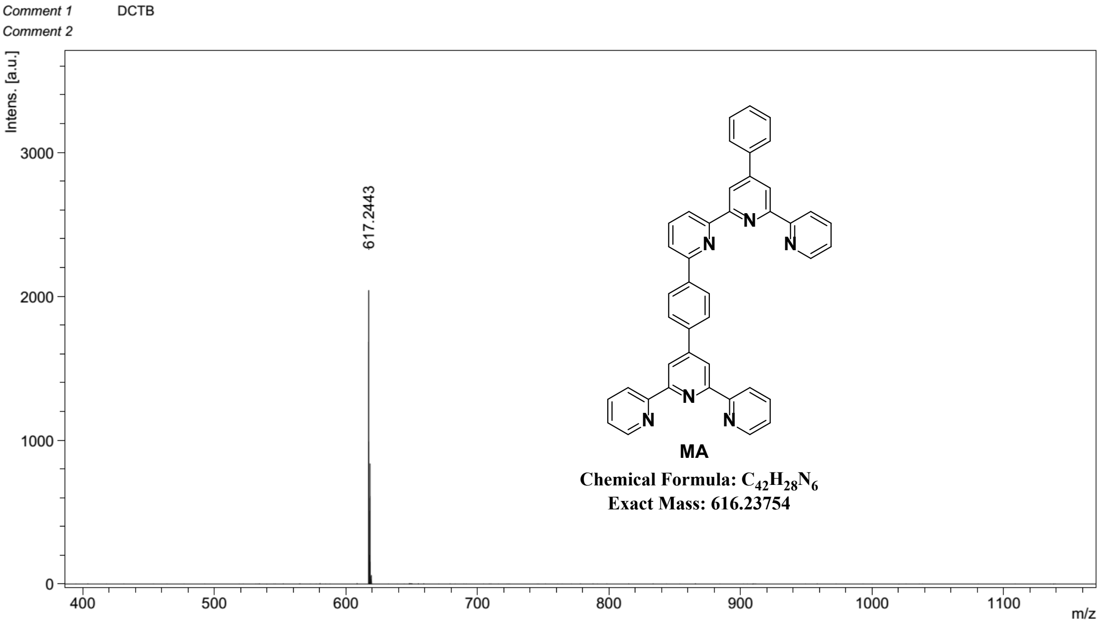

Supplementary Figure 3. MALDI-TOF plot of **MA**.

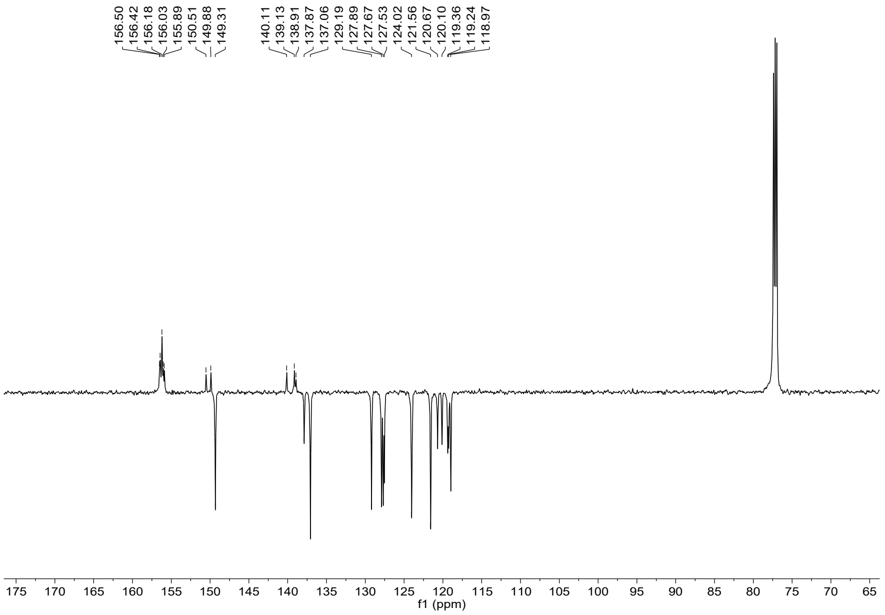

Supplementary Figure 4. DEPTQ ^{13}C NMR (150 MHz, $CDCl_3$, 300 K) spectrum of **MA**.

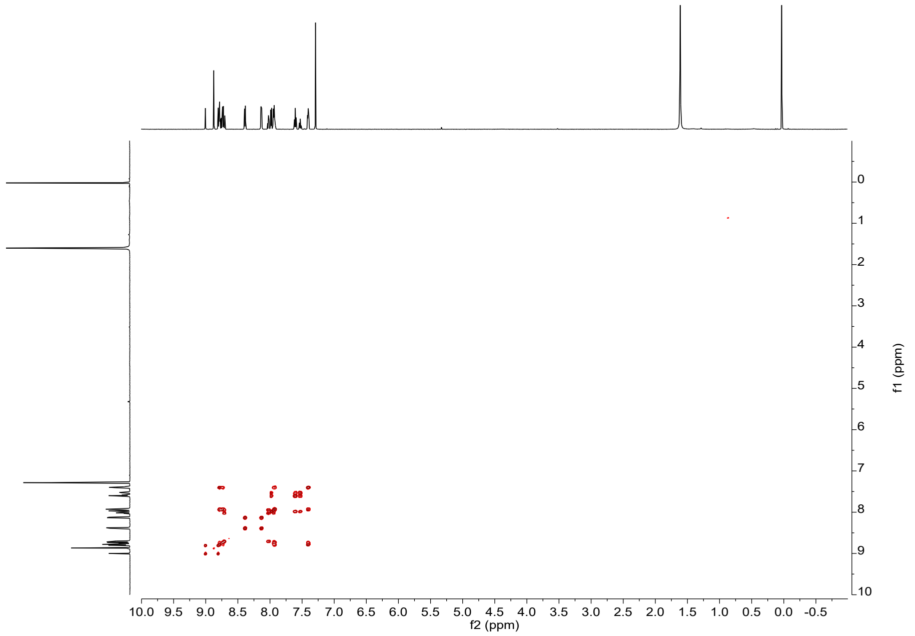

Supplementary Figure 5. 2D COSY NMR (600 MHz, $CDCl_3$, 300 K) spectrum of **MA**.

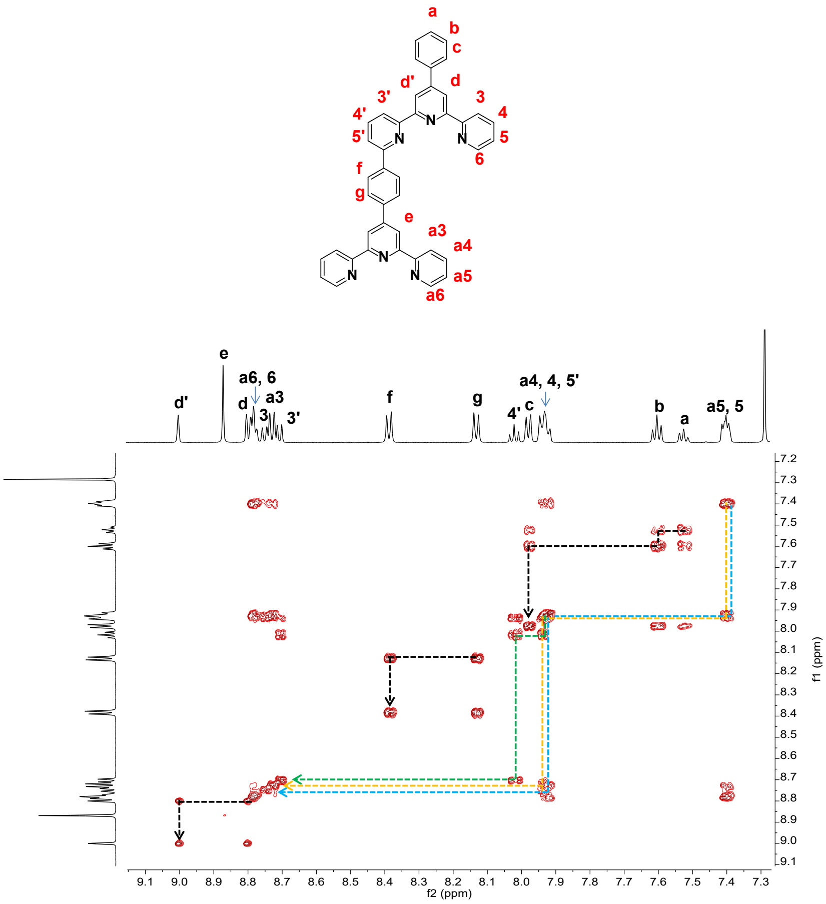

Supplementary Figure 6. 2D COSY NMR (600 MHz, $CDCl_3$, 300 K) spectrum of **MA** (aromatic region).

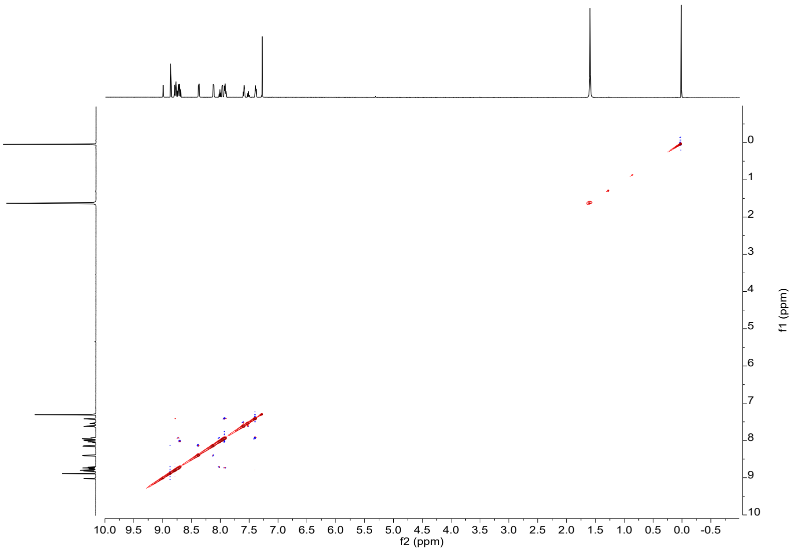

Supplementary Figure 7. 2D NOESY NMR (600 MHz, $CDCl_3$, 300 K) spectrum of **MA**.

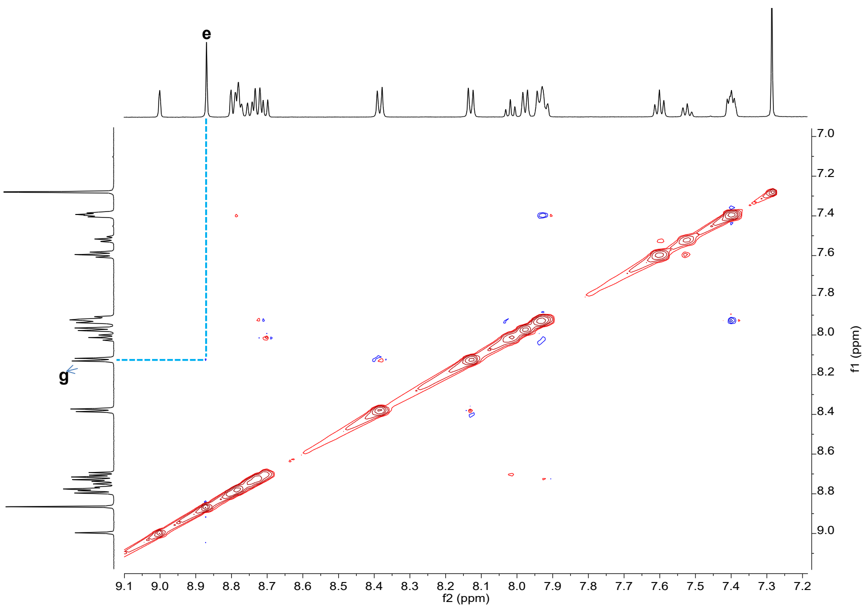

Supplementary Figure 8. 2D NOESY NMR (600 MHz, $CDCl_3$, 300 K) spectrum of **MA** (aromatic region)

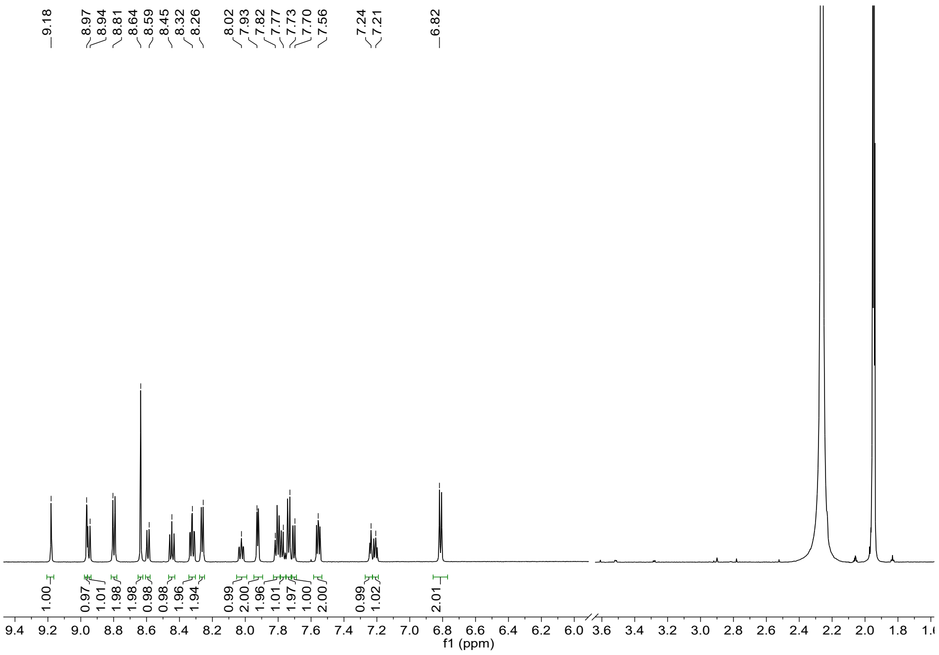

Supplementary Figure 9. ^{1}H NMR (600 MHz, CD_3CN, 300 K) spectrum of complex **$Zn_2(MA)_2$**.

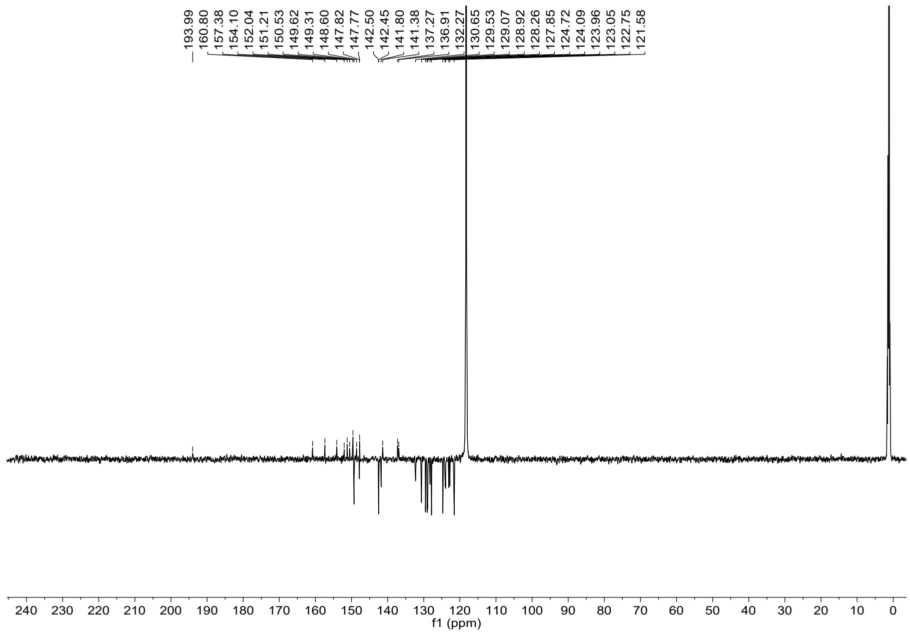

Supplementary Figure 10. DEPTQ ^{13}C NMR (150 MHz, CD_3CN, 300 K) spectrum of complex **$Zn_2(MA)_2$**.

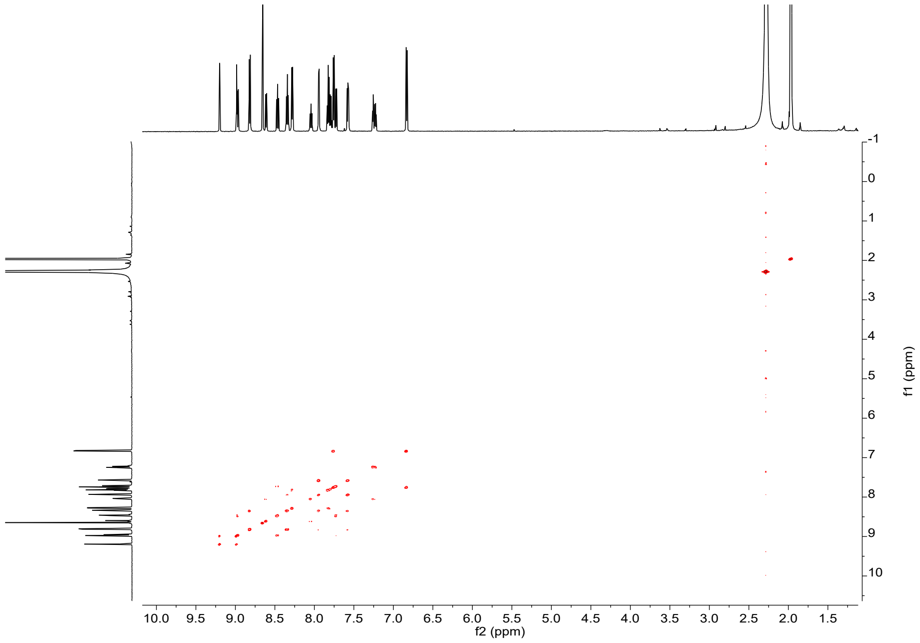

Supplementary Figure 11. 2D COSY NMR (600 MHz, CD_3CN, 300 K) spectrum of complex **$Zn_2(MA)_2$**.

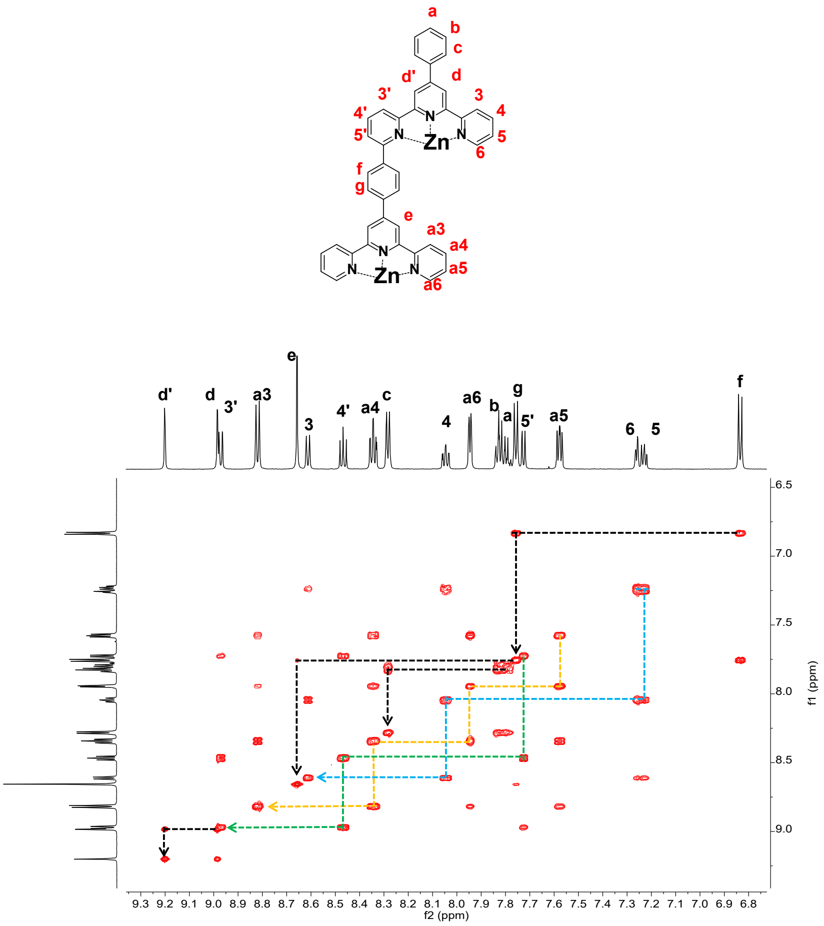

Supplementary Figure 12. 2D COSY NMR (600 MHz, CD_3CN, 300 K) spectrum of ligand **$Zn_2(MA)_2$** (aromatic region).

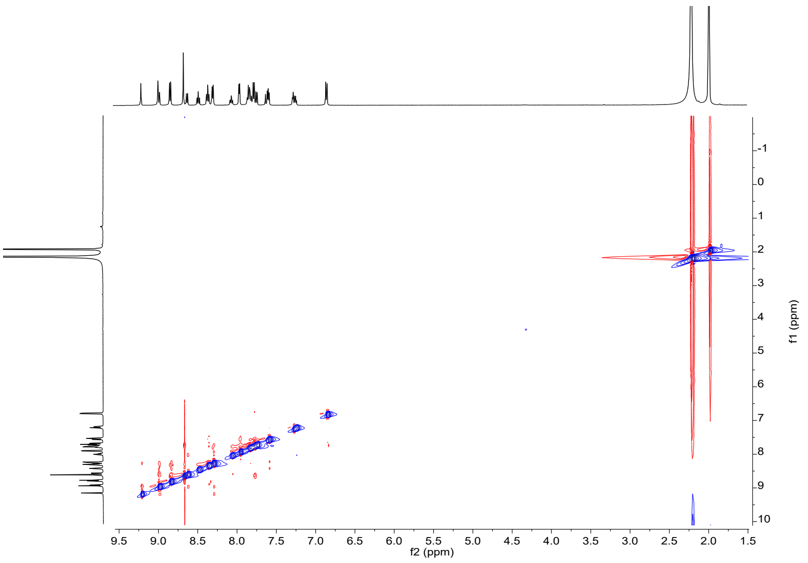

Supplementary Figure 13. 2D NOESY NMR (600 MHz, CD_3CN, 300 K) spectrum of complex **$Zn_2(MA)_2$**.

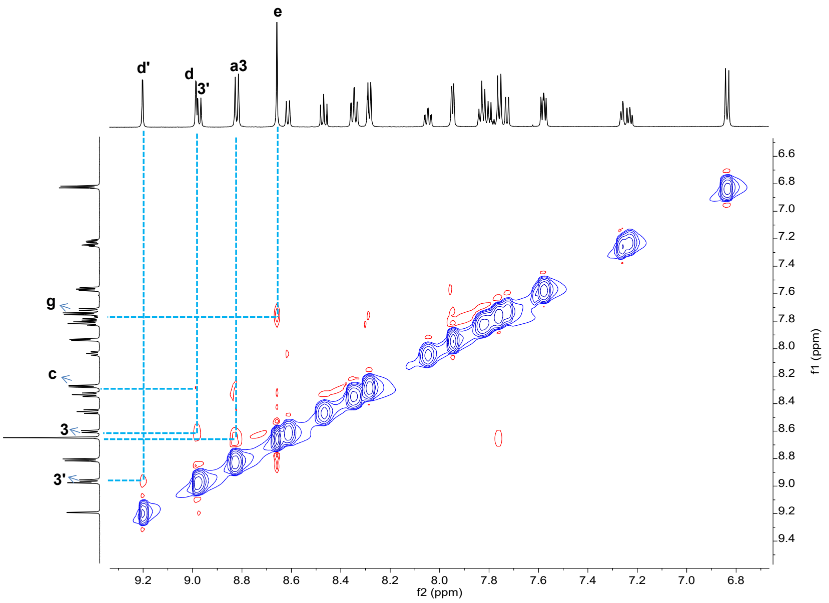

Supplementary Figure 14. 2D NOESY NMR (600 MHz, CD_3CN, 300 K) spectrum of ligand **$Zn_2(MA)_2$** (aromatic region).

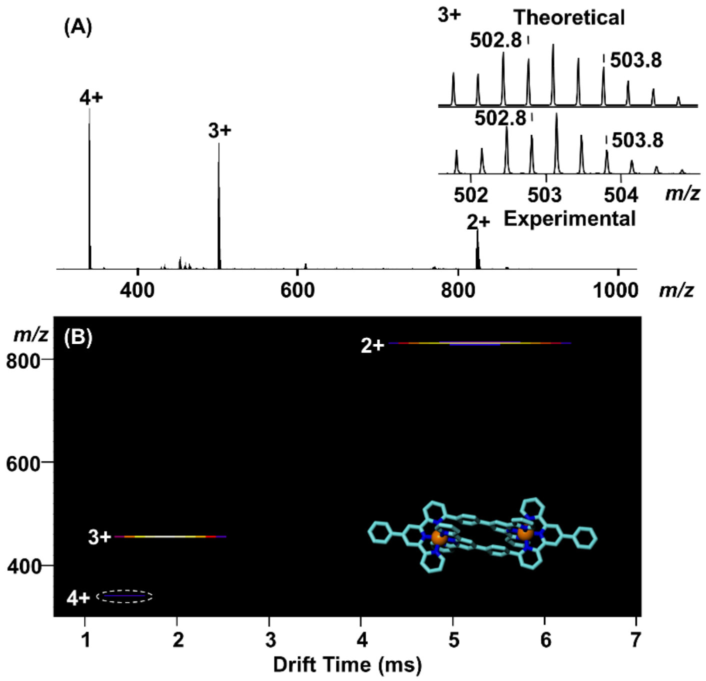

Supplementary Figure 15. (A) ESI-MS and (B) TWIM-MS plots (*m/z* vs drift time) of **$Zn_2(MA)_2$**.

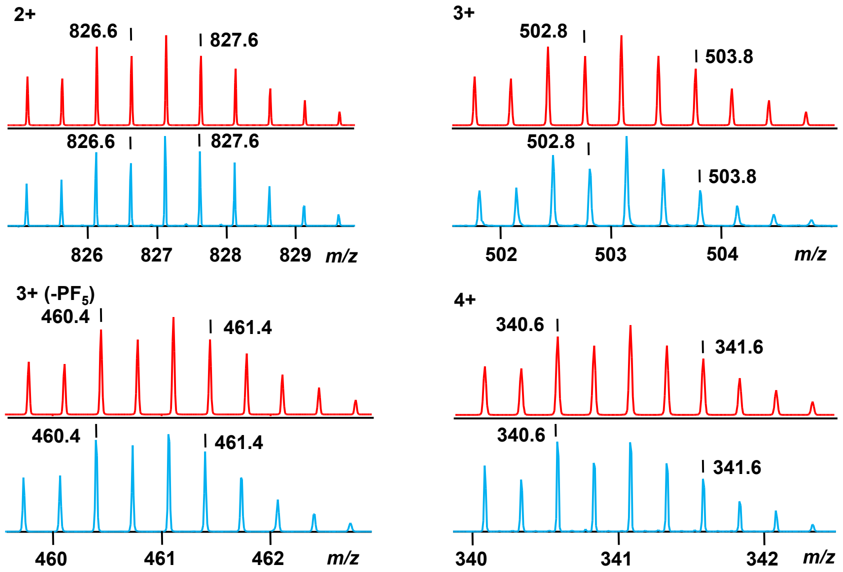

Supplementary Figure 16. Measured (blue) and calculated (red) isotope patterns for different charge states observed from **$Zn_2(MA)_2$** (PF_6^- as counterion).

Supplementary Table 1. Crystal data and structure refinement for **$Zn_2(MA)_2$**.

Identification code	$Zn_2(MA)_2$
Empirical formula	$C_{92}H_{68}F_{24}N_{16}P_4Zn_2$
Formula weight	2108.28
Temperature/K	110.0
Crystal system	monoclinic
Space group	$P2_1/c$
a/Å	17.5341(5)
b/Å	14.7590(4)
c/Å	16.3938(4)
α/°	90.00
β/°	91.5110(10)
γ/°	90.00
Volume/Å^3	4241.0(2)
Z	4
ρ_{calc}g/cm^3	1.651
μ/mm^{-1}	0.755
F(000)	2136.0
Crystal size/mm^3	0.12 × 0.11 × 0.1
Radiation	MoKα (λ = 0.71073)
2Θ range for data collection/°	5.9 to 54.22
Index ranges	$-22 \leq h \leq 22$, $-18 \leq k \leq 18$, $-21 \leq l \leq 21$
Reflections collected	60690
Independent reflections	9345 [R_{int} = 0.0479, R_{sigma} = 0.0293]
Data/restraints/parameters	9345/0/624
Goodness-of-fit on F^2	1.026

Final R indexes [I>=2σ (I)]	R_1 = 0.0557, wR_2 = 0.1474
Final R indexes [all data]	R_1 = 0.0718, wR_2 = 0.1576
Largest diff. peak/hole / e Å^{-3}	1.03/-0.72

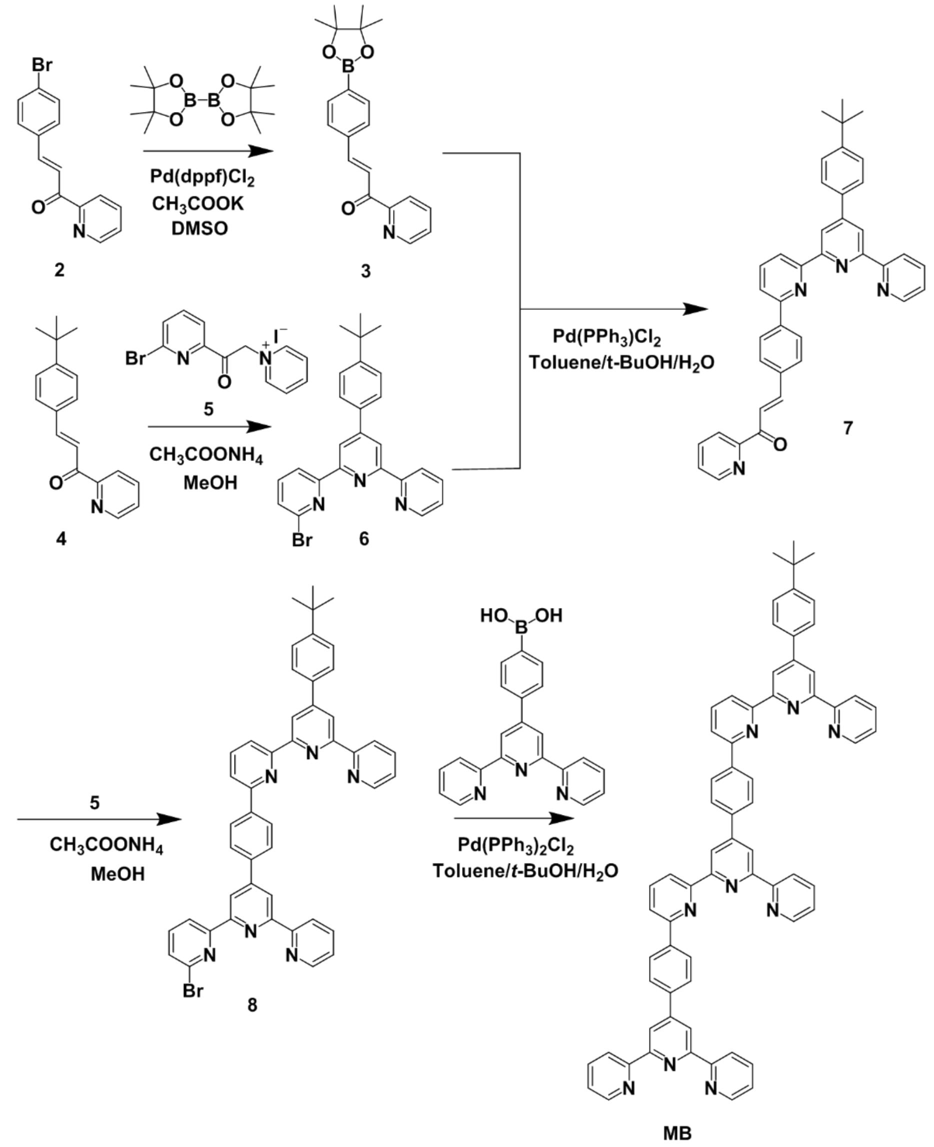

Supplementary Figure 17. Synthesis of ligand **MB**.

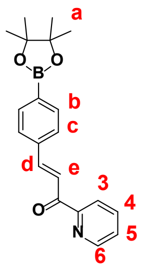

Compound 3: Compound **2**[2] (2.1 g, 7.4 mmol), *bis*-(pinacolato)diboron (2.3 g, 8.9

mmol), $Pd(dppf)Cl_2$ (216 mg, 0.3 mmol) and potassium acetate (2.9 g, 30 mmol) were added into a Schlenk flask. After that, the flask was degassed three times and added 32 mL anhydrous DMSO. The mixture was stirred at 85 °C for 14 h and then cooled to room temperature. The solution was extracted three times with $CHCl_3$ and then the solvent was removed under reduced pressure. The crude product was purified by column chromatography on silica gel (DCM) to give compound **3** as green solid (1.83 g, 74%). ^{1}H NMR (400 MHz, $CDCl_3$, 300 K) δ 8.74 (s, 1H, Ph-H^6), 8.32 (s, 1H, Ph-H^3), 8.18 (s, 1H, Ph-H^d), 7.96 (s, 1H, Ph-H^e), 7.87-7.84 (d, J = 13.3 Hz, 3H, Ph-H^4 and Ph-H^c), 7.73 (s, 2H, Ph-H^b), 7.50-7.47 (d, J = 12.3 Hz, 1H, Ph-H^5), 1.35 (s, 12H, H^a). ^{13}C NMR (100 MHz, $CDCl_3$, 300 K) δ 189.32, 154.12, 148.86, 144.46, 137.59, 137.00, 135.20, 127.97, 126.93, 122.90, 121.61, 83.99, 25.03, 24.89.

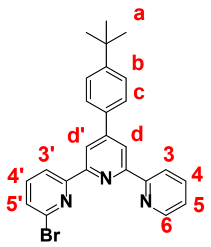

Compound 6: To a 30 mL anhydrous MeOH solution of compound **4**[3] (5.56 g, 21 mmol), pyridinium salt **5**[4] (11.0 g, 27.3 mmol) and NH_4OAc (33.0 g, 400 mmol) were added. The solution was refluxed overnight and then cooled to the room temperature. The formed precipitate was filtered and washed by MeOH to afford white solid (5.8 g, 63%). ^{1}H NMR (400 MHz, $CDCl_3$, 300 K) δ 8.77 – 8.71 (m, 2H, tpy-$H^{d'}$ and tpy-H^6), 8.69 (t, J = 1.3 Hz, 1H, tpy-H^d), 8.62 (dq, J = 7.9, 1.3 Hz, 2H, tpy-H^3 and tpy-$H^{3'}$), 7.92 – 7.80 (m, 3H, tpy-H^4 and Ph-H^b), 7.72 (td, J = 7.8, 1.5 Hz, 1H, tpy-$H^{4'}$), 7.58 – 7.48 (m, 3H, Ph-H^c and tpy-$H^{5'}$), 7.35 (ddt, J = 7.4, 4.9, 1.2 Hz, 1H, tpy-H^5), 1.39 (s, 9H, H^a). ^{13}C NMR (100 MHz, $CDCl_3$, 300 K) δ 157.70, 156.25, 156.06, 154.35, 152.52, 150.50, 149.31, 141.71, 139.25, 136.99, 135.50, 128.14, 127.18, 126.05, 124.00, 121.44, 120.12, 119.42, 119.29, 34.88, 31.45.

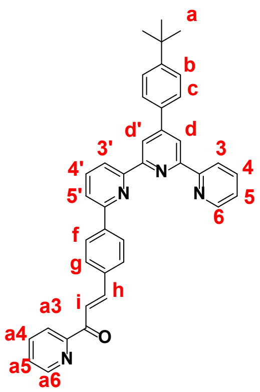

Compound 7: Compound **6** (1.87 g, 4.23 mmol), compound **3** (1.83 g, 5.5 mmol) and $Pd(PPh_3)_2Cl_2$ (300 mg, 0.4 mmol) were added into 200 mL Schlenk flask and degassed three times. Then toluene (80 mL), 1 M Na_2CO_3 (40 mL) and *tert*-butyl alcohol (20 mL) were added under N_2. The resultant mixture was kept at 85 °C for 24 h. After cooling down to the room temperature, the solution was extracted three times with $CHCl_3$, and the solvent was removed under reduced pressure. The crude product was purified by column chromatography on silica gel (DCM/EtOH=100/1) to give compound **7** as white solid (1.85 g, 76%). ^{1}H NMR (400 MHz, $CDCl_3$, 300 K) δ 8.94 (d, J = 1.7 Hz, 1H, tpy-$H^{d'}$), 8.76 (tt, J = 5.8, 3.0 Hz, 3H, Ph-H^{a6}, tpy-H^{d} and tpy-H^{6}), 8.70 (d, J = 8.0 Hz, 1H, tpy-H^{3}), 8.67 (d, J = 7.8 Hz, 1H, tpy-$H^{3'}$), 8.41 (d, J = 16.0 Hz, 1H, H^{i}), 8.27 (d, J = 8.2 Hz, 2H, Ph-H^{b}), 8.22 (dt, J = 7.9, 1.1 Hz, 1H, tpy-H^{a3}), 8.03 (d, J = 16.1 Hz, 1H, H^{h}), 7.97 (t, J = 7.8 Hz, 1H, tpy-$H^{4'}$), 7.92 – 7.85 (m, 7H, tpy-$H^{5'}$, Ph-H^{c}, Ph-H^{f}, tpy-H^{4} and Ph-H^{a4}), 7.61 – 7.57 (m, 2H, Ph-H^{g}), 7.51 (ddd, J = 7.6, 4.7, 1.2 Hz, 1H, Ph-H^{a5}), 7.36 (ddd, J = 7.5, 4.7, 1.2 Hz, 1H, tpy-H^{5}), 1.41 (s, 9H, H^{a}). ^{13}C NMR (100 MHz, $CDCl_3$, 300 K) δ 189.59, 156.51, 156.26, 156.00, 155.95, 155.49, 154.36, 152.42, 150.29, 149.28, 149.02, 144.37, 141.40, 137.85, 137.19, 136.99, 135.91, 135.87, 129.47, 127.50, 127.18, 127.08, 126.12, 123.91, 123.11, 121.50, 121.34, 121.30, 120.60, 120.24, 119.10, 34.91, 31.49.

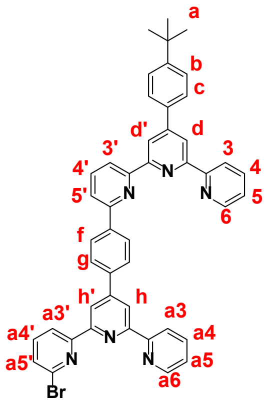

Compound 8: A mixture of NH_4OAc (5.0 g, 65 mmol), pyridinium salt **5** (1.96 g, 4.85 mmol) and compound **7** (1.85 g, 3.23 mmol) was added into 50 mL anhydrous MeOH, 50 mL anhydrous DMF and 20 mL AcOH. The solution was refluxed for 24 h, and then cooled to room temperature. The formed precipitate was filtered and washed by MeOH to afford yellow solid (5.8 g, 78%). ^{1}H NMR (400 MHz, $CDCl_3$, 300 K) δ 8.97 (d, J = 1.7 Hz, 1H, tpy-$H^{d'}$), 8.82 (d, J = 1.7 Hz, 1H, tpy-H^{d}), 8.77 (d, J = 1.7 Hz, 1H, tpy-$H^{h'}$), 8.75 – 8.72 (m, 3H, tpy-H^{6}, tpy-H^{a6} and tpy-H^{h}), 8.72 – 8.68 (m, 1H, tpy-H^{3}), 8.65 (td, J = 7.7, 1.0 Hz, 3H, tpy-H^{a3}, tpy-$H^{3'}$ and tpy-$H^{a3'}$), 8.39 – 8.32 (m, 2H, tpy-H^{g}), 8.11 – 8.04 (m, 2H, tpy-H^{f}), 7.97 (t, J = 7.8 Hz, 1H, tpy-$H^{4'}$), 7.92 – 7.86 (m, 5H, Ph-H^{b}, tpy-H^{4}, tpy-H^{a4} and tpy-$H^{5'}$), 7.73 (t, J = 7.7 Hz, 1H, tpy-$H^{a4'}$), 7.62 – 7.57 (m, 2H, Ph-H^{c}), 7.54 (dd, J = 7.8, 0.9 Hz, 1H, tpy-$H^{a5'}$), 7.37 (m, J = 7.4, 4.5, 1.2 Hz, 2H, tpy-H^{a5} and tpy-H^{5}), 1.40 (s, 9H, H^{a}). ^{13}C NMR (100 MHz, $CDCl_3$, 300 K) δ 157.62, 156.55, 156.20, 156.18, 156.12, 156.08, 155.91, 155.66, 154.51, 152.40, 150.22, 150.04, 149.34, 149.27, 141.76, 140.15, 139.31, 138.80, 137.84, 137.05, 137.00, 135.86, 128.26, 127.87, 127.65, 127.17, 126.15, 124.12, 123.90, 121.54, 121.48, 120.55, 120.21, 120.07, 119.47, 119.23, 119.12, 119.00, 34.92, 31.50.

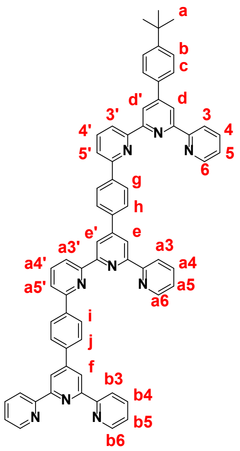

MB: A mixture of compound **8** (750 mg, 1 mmol), 4-boronatophenyl terpyridine (706 mg, 2 mmol) and $Pd(PPh_3)_2Cl_2$ (56 mg, 0.08 mmol) in 100 mL Schlenk flask was degassed three times. Then toluene (60 mL), 1 M Na_2CO_3 (30 mL) and *tert*-butyl alcohol (15 mL) were added under N_2. The resultant mixture was kept at 85 ^{o}C for 24 h. After cooling to the room temperature, the solution was extracted three times with $CHCl_3$, and the solvent was removed under reduced pressure. The crude product was purified by column chromatography on silica gel ($CHCl_3$/EtOH=70/1) to give compound **MB** as white solid (734 mg, 75%). ^{1}H NMR (600 MHz, $CDCl_3$, 300 K) δ 9.05 (d, J = 1.7 Hz, 1H, tpy-$H^{e'}$), 8.99 (d, J = 1.7 Hz, 1H, tpy-$H^{d'}$), 8.87 (d, J = 1.7 Hz, 1H, tpy-H^{e}), 8.84 (s, 2H, tpy-H^{f}), 8.77 (ddd, J = 4.6, 1.8, 0.9 Hz, 1H, tpy-H^{a6}), 8.76 (d, J = 1.7 Hz, 1H, tpy-H^{d}), 8.75 – 8.70 (m, 5H, tpy-H^{a3}, tpy-H^{6}, tpy-H^{3} and tpy-H^{b6}), 8.69 (dt, J = 7.9, 1.1 Hz, 3H, tpy-H^{b3} and tpy-$H^{a3'}$), 8.67 (dd, J = 7.7, 1.0 Hz, 1H, tpy-$H^{3'}$), 8.44 – 8.40 (m, 2H, Ph-H^{g}), 8.39 – 8.35 (m, 2H, Ph-H^{i}), 8.15 – 8.09 (m, 4H, Ph-H^{h} and Ph-H^{j}), 7.99 (dt, J = 9.2, 7.7 Hz, 2H, tpy-$H^{a4'}$ and tpy-$H^{4'}$), 7.94 – 7.86 (m, 8H, tpy-H^{4}, tpy-$H^{5'}$, Ph-H^{c}, tpy-H^{a4}, tpy-$H^{a5'}$ and tpy-H^{b4}), 7.57 – 7.53 (m, 2H, Ph-H^{b}), 7.40 – 7.36 (m, 1H, tpy-H^{a5}), 7.36 – 7.33 (m, 3H, tpy-H^{5} and tpy-H^{b5}), 1.31 (s, 9H, H^{a}). ^{13}C NMR (150 MHz, $CDCl_3$, 300 K) δ 156.63, 156.49, 156.40, 156.31, 156.21, 156.17, 156.12, 155.93, 155.83, 152.40, 150.29, 149.88, 149.84, 149.31, 140.11, 139.31, 139.14, 137.86, 137.81, 137.04, 136.99, 135.90, 127.89, 127.76, 127.69, 127.16, 126.13, 123.98, 123.87, 121.58, 121.54, 121.50, 120.70, 120.54, 120.10, 120.02, 119.22, 119.05, 119.01, 118.93, 31.39. MALDI-TOF MS (*m/z*): Calcd. for $[C_{67}H_{49}N_9+H]^+$ 980.42. Found: 980.42.

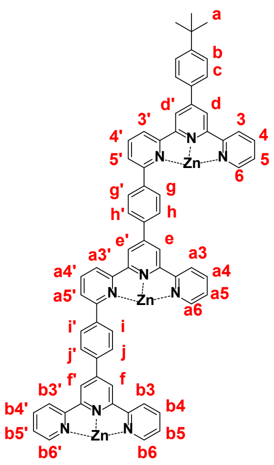

Complex $Zn_3(MB)_2$: To a solution of **MB** (4.4 mg, 3.9 μmol) in $CHCl_3$ (1.0 mL), a solution of $Zn(NO_3)_2$•$6H_2O$ (1.8 mg, 5.9 μmol) in MeOH (3.0 mL) was added, then the mixture was kept in a 50 °C for 12 h. After cooling to room temperature, excess NH_4PF_6 (around 80 mg) was added to generate a white precipitate (5.1 mg, 87%). ^{1}H NMR (600 MHz, CD_3CN, 300 K) δ 9.20 – 9.16 (m, 1H, tpy-$H^{d'}$), 9.03(d, 1H, tpy-H^{a3}), 8.97-8.95 (m, 2H, tpy-H^{d} and tpy-$H^{a3'}$), 8.87 (d, 1H, tpy-$H^{3'}$), 8.83 (s, 1H, tpy-$H^{e'}$), 8.72 – 8.70 (m, 3H, tpy-H^{b3}, tpy-$H^{b3'}$ and tpy-$H^{f'}$), 8.67 (s, 1H, tpy-H^{f}), 8.63 (s, 1H, tpy-H^{e}), 8.61 – 8.56 (m, 2H, tpy-H^{3} and tpy-$H^{4'}$), 8.45 (t, 1H, tpy-$H^{a4'}$), 8.41 (t, 1H, tpy-H^{a4}), 8.27 (t, 1H, tpy-$H^{b4'}$), 8.22 (d, 2H, Ph-H^{c}), 8.18 (m, 1H, tpy-H^{b4}), 8.10 (d, 1H, Ph-$H^{j'}$), 8.01 (t, 1H, tpy-H^{4}), 7.97 (d, J = 5.4 Hz, 1H, tpy-H^{a6}), 7.94 – 7.81 (m, 5H, Ph-$H^{h'}$, tpy-$H^{b6'}$, Ph-H^{b} and tpy-$H^{5'}$), 7.73-7.69 (dd, J = 14.9, 7.5 Hz, 2H, tpy-$H^{a5'}$ and Ph-H^{h}), 7.64 (d, 1H, tpy-H^{b6}), 7.59 (t, J = 7.4 Hz, 1H, tpy-H^{a5}), 7.55 (t, 1H, tpy-$H^{b5'}$), 7.48 (d, 1H, tpy-H^{j}), 7.41 (t, 1H, tpy-H^{b5}), 7.27 (dd, J = 7.5, 2.0 Hz, 1H, tpy-H^{6}), 7.24 (t, 1H, tpy-H^{5}), 7.08 (d, 1H, Ph-$H^{i'}$), 6.87 (d, J = 7.4 Hz, 1H, Ph-$H^{g'}$), 6.82 (d, 1H, Ph-H^{g}), 6.76 (dd, J = 7.5, 2.0 Hz, 1H, Ph-H^{i}), 1.48 (s, 9H, H^{a}). ^{13}C NMR (100 MHz, CD_3CN, 300 K) δ 159.87, 156.20, 155.15, 153.43, 153.35, 151.13, 151.11, 150.27, 149.76, 149.61, 149.06, 149.01, 148.84, 148.83, 148.41, 147.83, 147.77, 146.94, 146.86, 146.81, 146.65, 141.80, 141.69, 141.51, 141.15, 140.87, 140.60, 139.82, 136.98, 136.20, 133.06, 129.65, 128.85, 128.63, 128.54, 128.13, 128.05, 127.95, 127.83, 127.35, 127.13, 126.75, 126.51, 124.07, 123.79, 123.20, 123.04, 121.81, 121.49, 121.37, 120.70, 34.77, 30.45, 30.09. ESI-MS (*m/z*): 1367.7 $[M-2PF_6^-]^{2+}$ (calcd *m/z*: 1367.7), 863.2 $[M-3PF_6^-]^{3+}$ (calcd *m/z*: 863.2), 611.1 $[M-4PF_6^-]^{4+}$ (calcd

m/z: 611.1), 460.1 [M-5PF_6^-]$^{5+}$ (calcd *m/z*: 460.1), 434.5 [M-5PF_6^--PF_5]$^{5+}$ (calcd *m/z*: 434.5), 359.1 [M-6PF_6^-]$^{6+}$ (calcd *m/z*: 359.1).

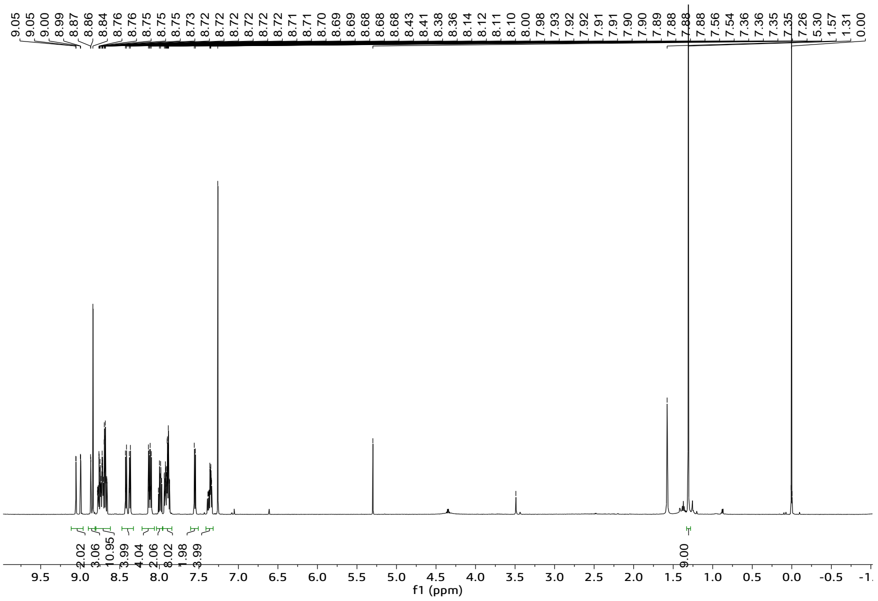

Supplementary Figure 18. ^{1}H NMR (600 MHz, $CDCl_3$, 300 K) spectrum of **MB**.

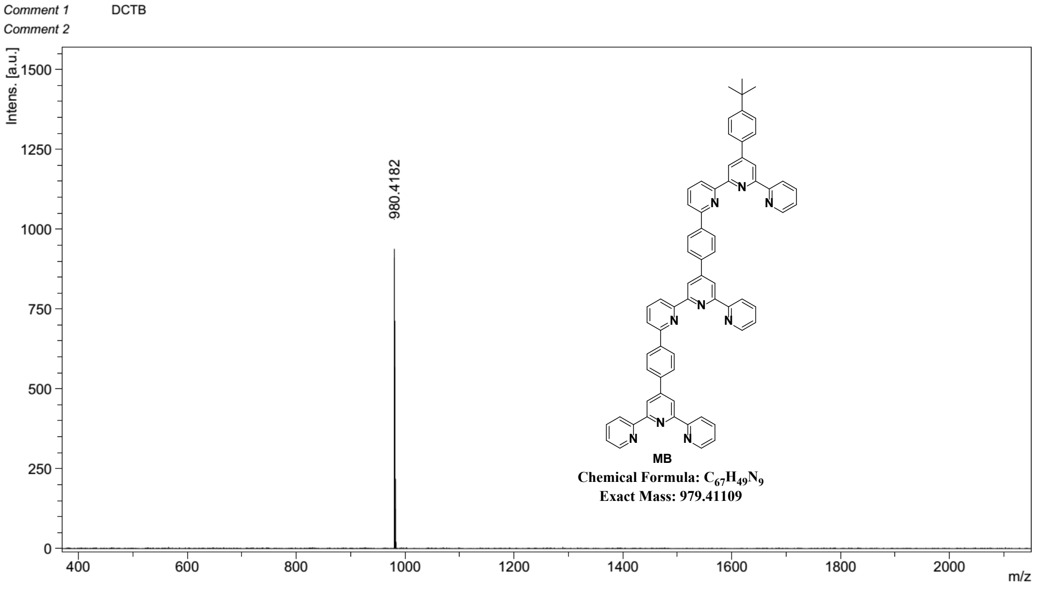

Supplementary Figure 19. MALDI-TOF plot of **MB**.

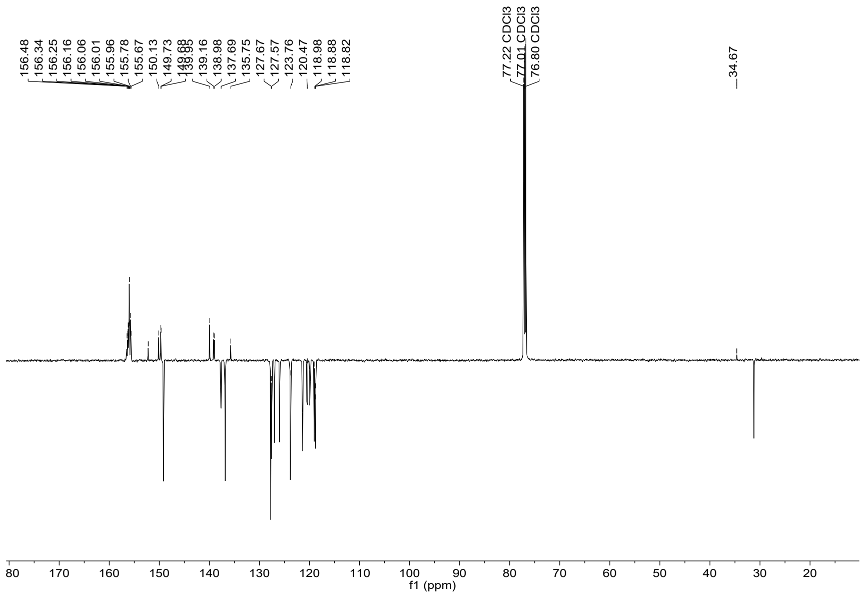

Supplementary Figure 20. DEPTQ ^{13}C NMR (150 MHz, $CDCl_3$, 300 K) spectrum of **MB**.

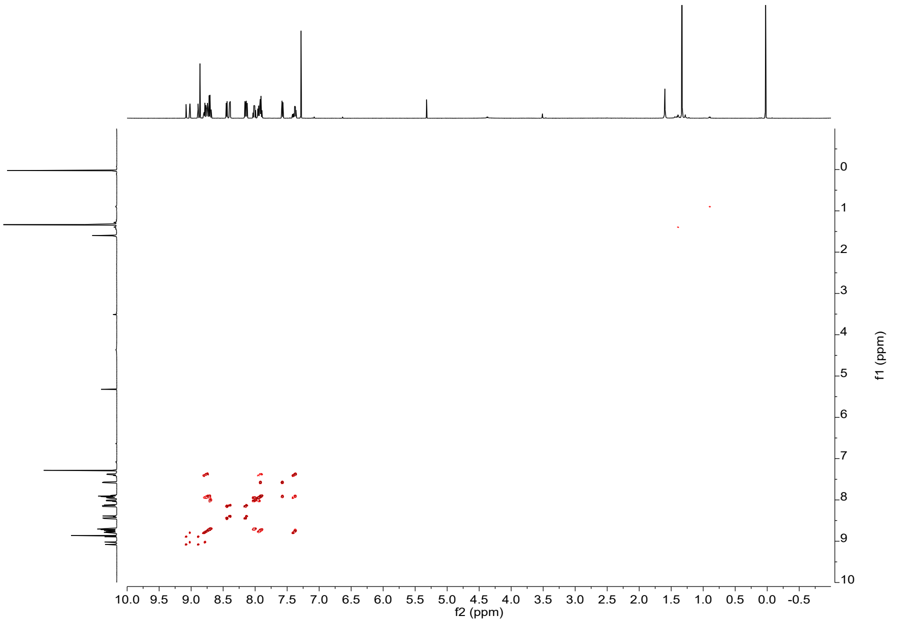

Supplementary Figure 21. 2D COSY NMR (600 MHz, $CDCl_3$, 300 K) spectrum of **MB**.

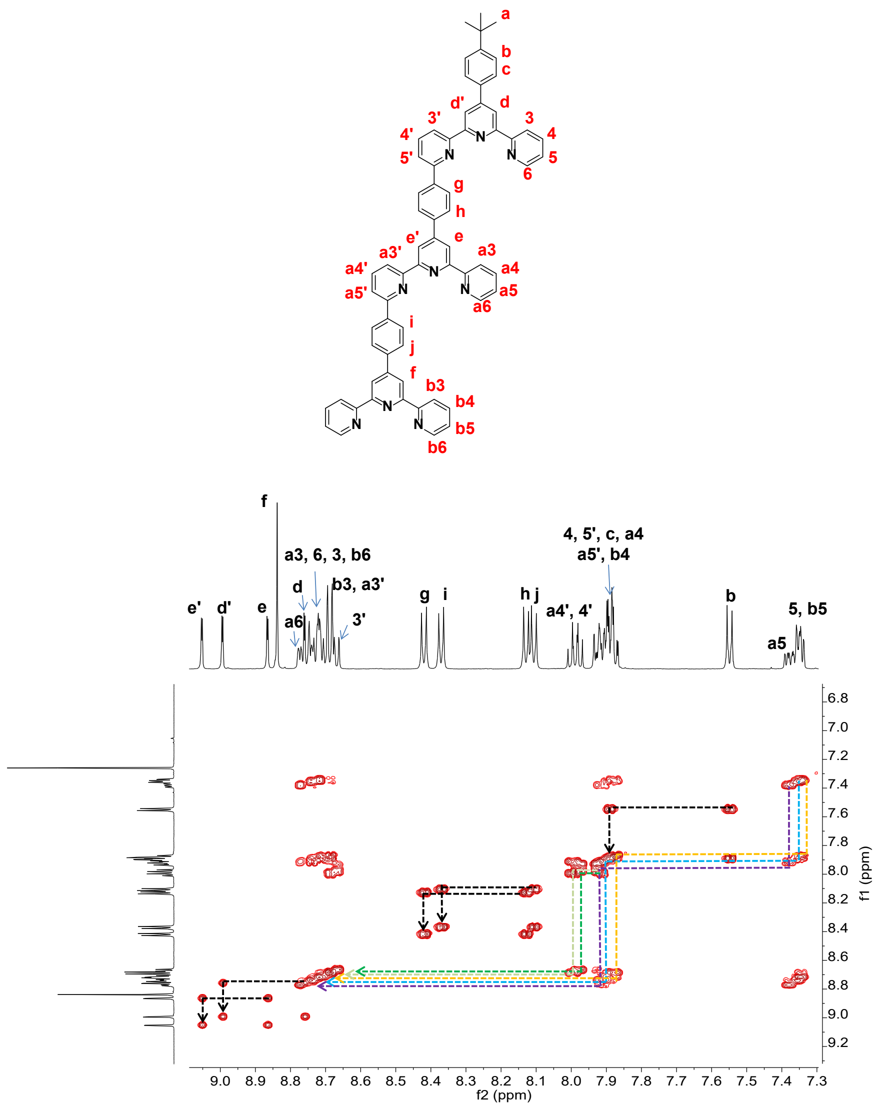

Supplementary Figure 22. 2D COSY NMR (600 MHz, $CDCl_3$, 300 K) spectrum of **MB** (aromatic region).

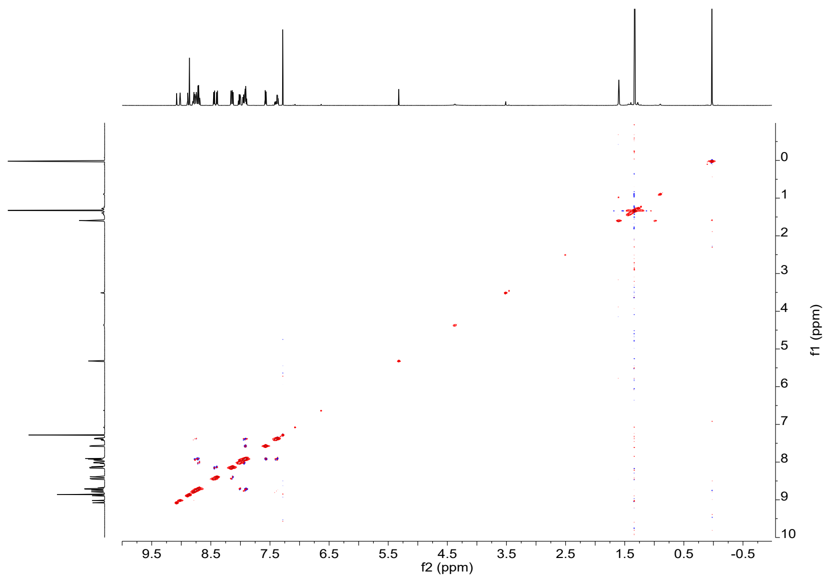

Supplementary Figure 23. 2D NOESY NMR (600 MHz, $CDCl_3$, 300 K) spectrum of **MB**.

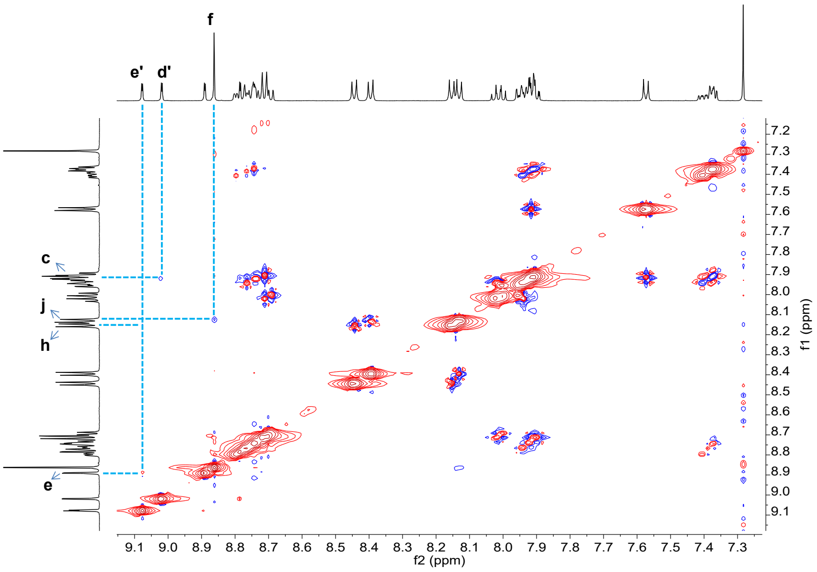

Supplementary Figure 24. 2D NOESY NMR (600 MHz, $CDCl_3$, 300 K) spectrum of **MB** (aromatic region).

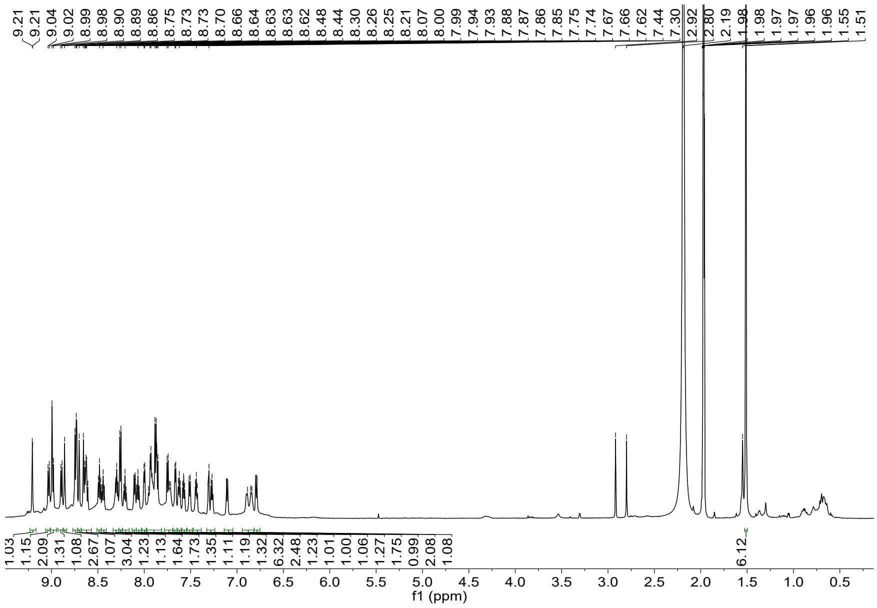

Supplementary Figure 25. ^1H NMR (600 MHz, CD_3CN, 300 K) spectrum of complex **$Zn_3(MB)_2$**.

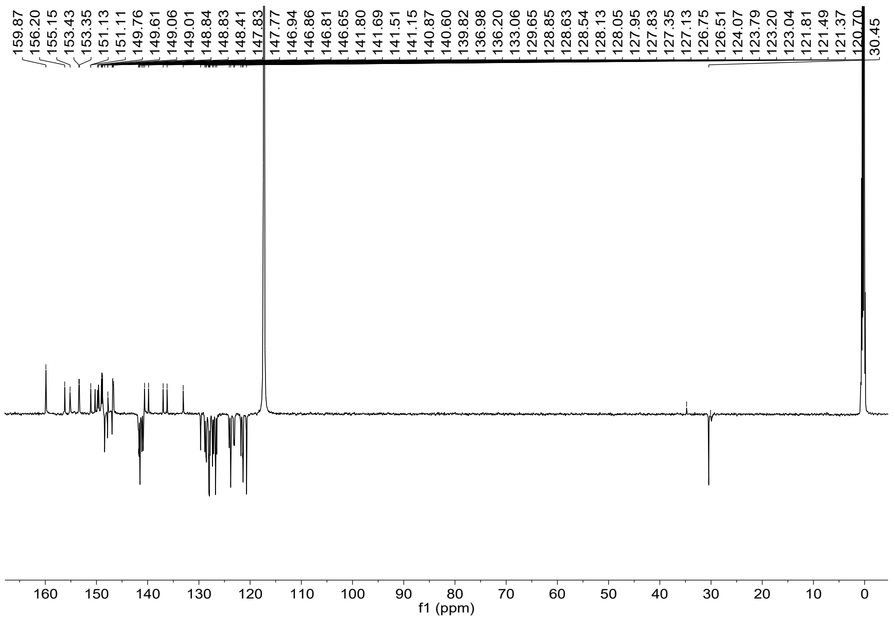

Supplementary Figure 26. DEPTQ ^{13}C NMR (150 MHz, CD_3CN, 300 K) spectrum of complex **$Zn_3(MB)_2$**.

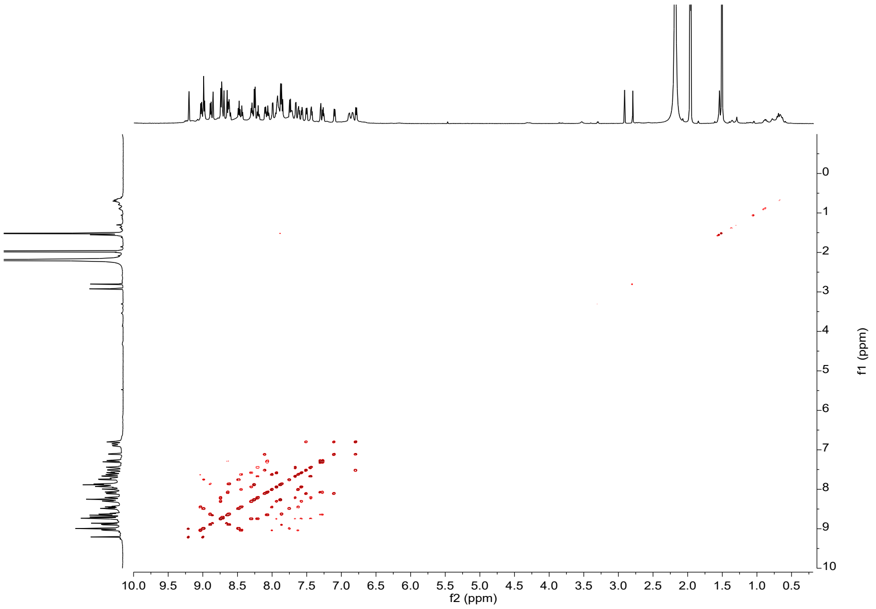

Supplementary Figure 27. 2D COSY NMR (600 MHz, CD_3CN, 300 K) spectrum of complex **$Zn_3(MB)_2$**.

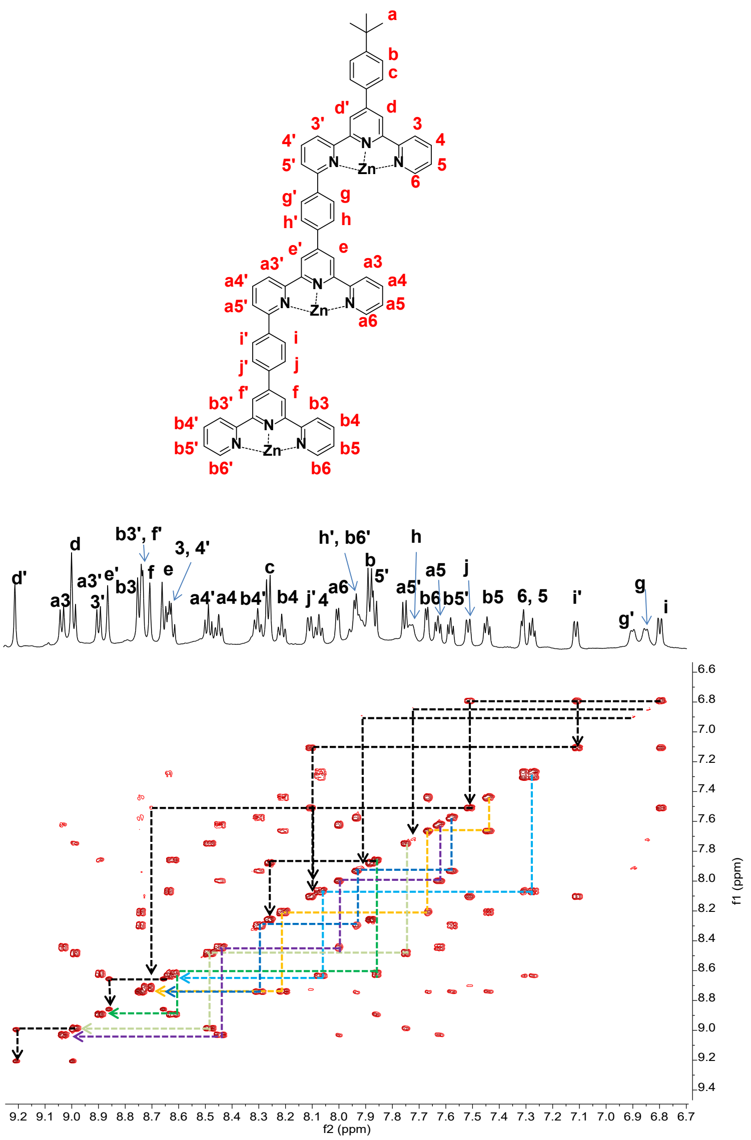

Supplementary Figure 28. 2D COSY NMR (600 MHz, CD_3CN, 300 K) spectrum of complex $\mathbf{Zn_3(MB)_2}$ (aromatic region).

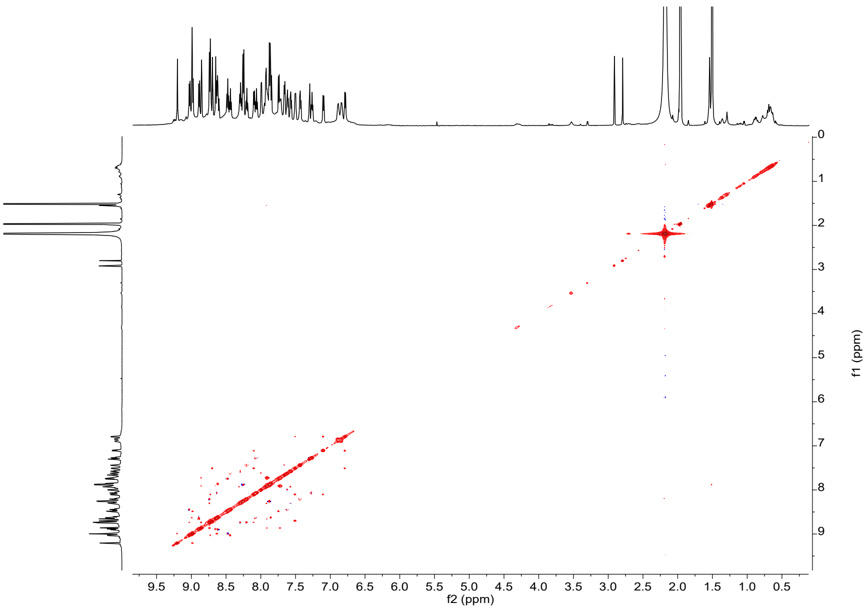

Supplementary Figure 29. 2D NOESY NMR (600 MHz, CD_3CN, 300 K) spectrum of complex **$Zn_3(MB)_2$**.

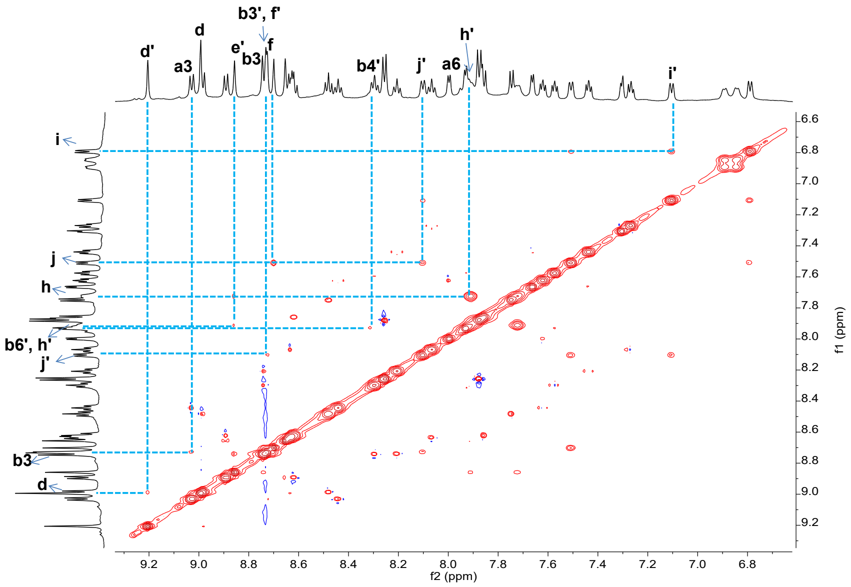

Supplementary Figure 30. 2D NOESY NMR (600 MHz, CD_3CN, 300 K) spectrum of complex **$Zn_3(MB)_2$** (aromatic region).

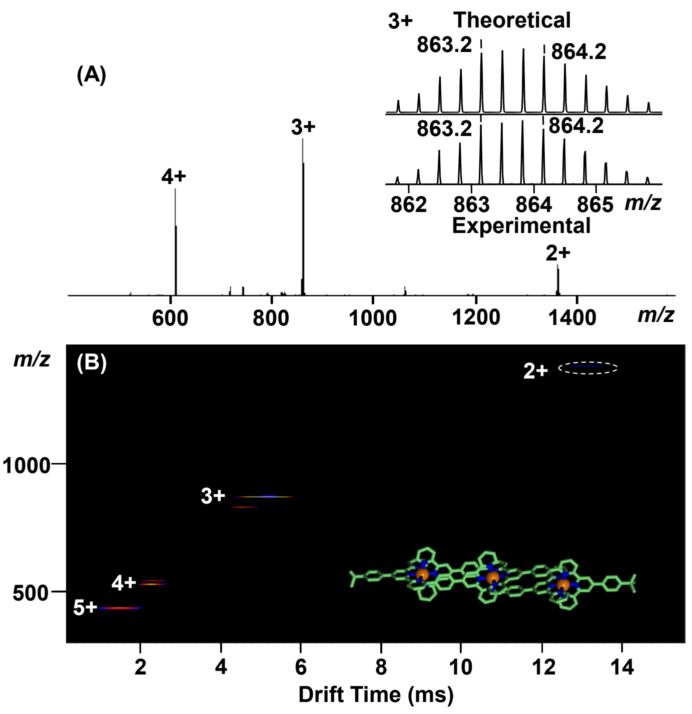

Supplementary Figure 31. (A) ESI-MS and (B) TWIM-MS plots (*m/z* vs drift time) of $\mathbf{Zn_3(MB)_2}$.

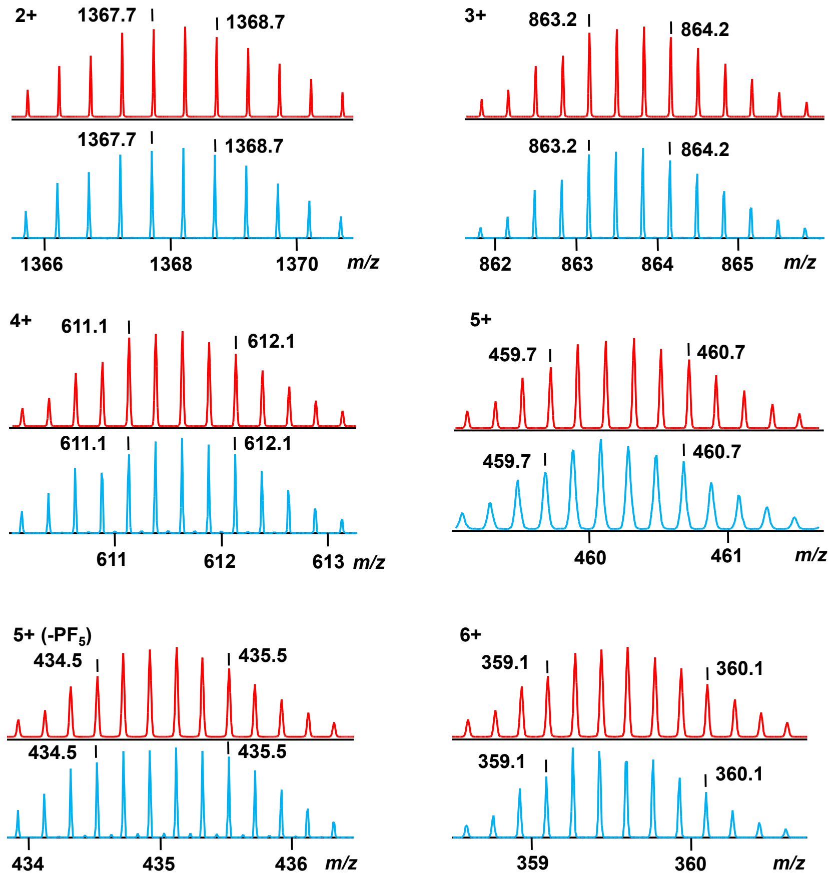

Supplementary Figure 32. Measured (blue) and calculated (red) isotope patterns for

different charge states observed from **$Zn_3(MB)_2$** (PF_6^- as counterion).

Supplementary Table 2. Crystal data and structure refinement for **$Zn_3(MB)_2$**.

Identification code	$Zn_3(MB)_2$
Empirical formula	$C_{145}H_{109}N_{22}Cl_{12}Zn_3P_6F_{36}$
Formula weight	3650.87
Temperature/K	273.15
Crystal system	triclinic
Space group	P-1
a/Å	13.5684(13)
b/Å	23.680(2)
c/Å	27.1640(19)
α/°	112.287(2)
β/°	90.731(2)
γ/°	105.632(2)
Volume/Å^3	7712.2(12)
Z	2
ρ_{calc}g/cm^3	1.572
μ/mm^{-1}	0.571
F(000)	3682.0
Crystal size/mm^3	0.14 × 0.12 × 0.11
Radiation	synchrotron (λ = 0.61978)
2Θ range for data collection/°	2.676 to 48.154
Index ranges	$-17 \le h \le 17$, $-31 \le k \le 31$, $-35 \le l \le 34$
Reflections collected	107207
Independent reflections	34364 [R_{int} = 0.1351, R_{sigma} = 0.1830]
Data/restraints/parameters	34364/60/1680
Goodness-of-fit on F^2	1.404
Final R indexes [I>=2σ (I)]	R_1 = 0.1762, wR_2 = 0.4158
Final R indexes [all data]	R_1 = 0.1963, wR_2 = 0.4386
Largest diff. peak/hole / e Å^{-3}	4.27/-2.51

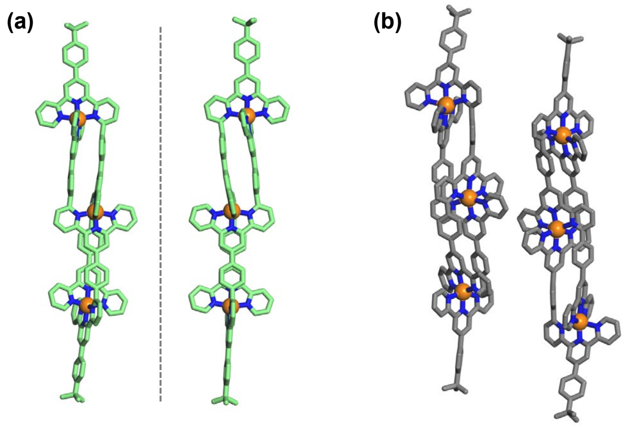

Supplementary Figure 33. (a) Mirror symmetry structure of **$Zn_3(MB)_2$** which were simulated by Material Studio. (b) X-ray crystal structure of **$Zn_3(MB)_2$**. H atoms, non-coordinated anions and solvent are omitted for clarity (C, gray or yellow; N, blue; Zn, orange).

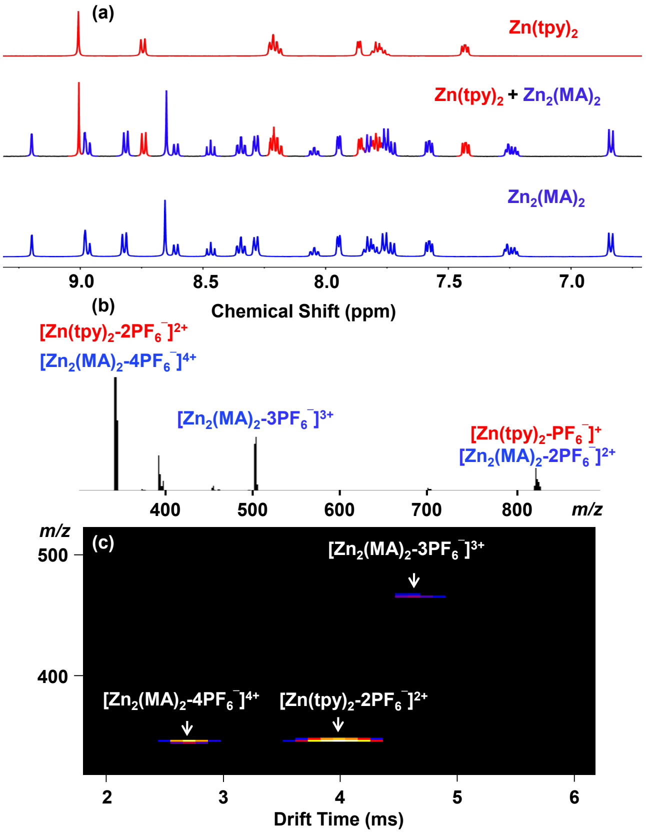

Supplementary Figure 34. (A) ^{1}H NMR spectra (500 MHz, CD_3CN, 300 K) of **$Zn(tpy)_2$** (top), **$Zn_2(MA)_2$** (bottom) and mixture of both (middle) (3 mg/mL); (B) ESI-MS and (C) TWIM-MS plots (*m/z* vs drift time) of the binary mixture.

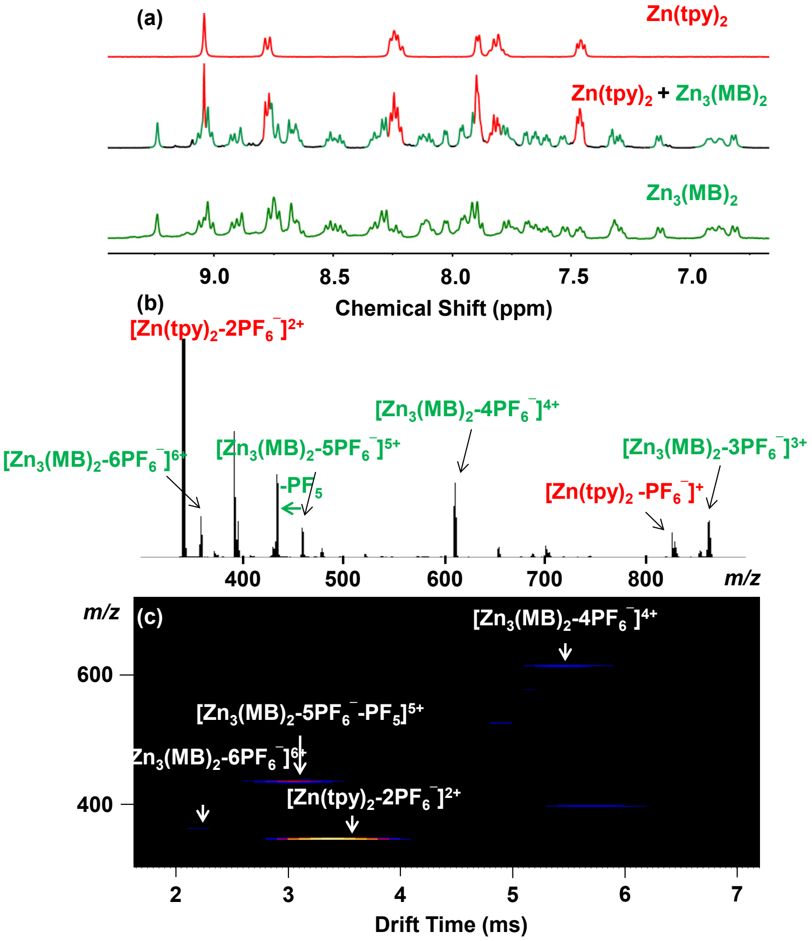

Supplementary Figure 35. (A) ^{1}H NMR spectra (500 MHz, CD_3CN, 300 K) of **Zn(tpy)$_2$** (top), **Zn$_3$(MB)$_2$** (bottom) and mixture of both (middle) (3 mg/mL); (B) ESI-MS and (C) TWIM-MS plots (*m/z* vs drift time) of the binary mixture.

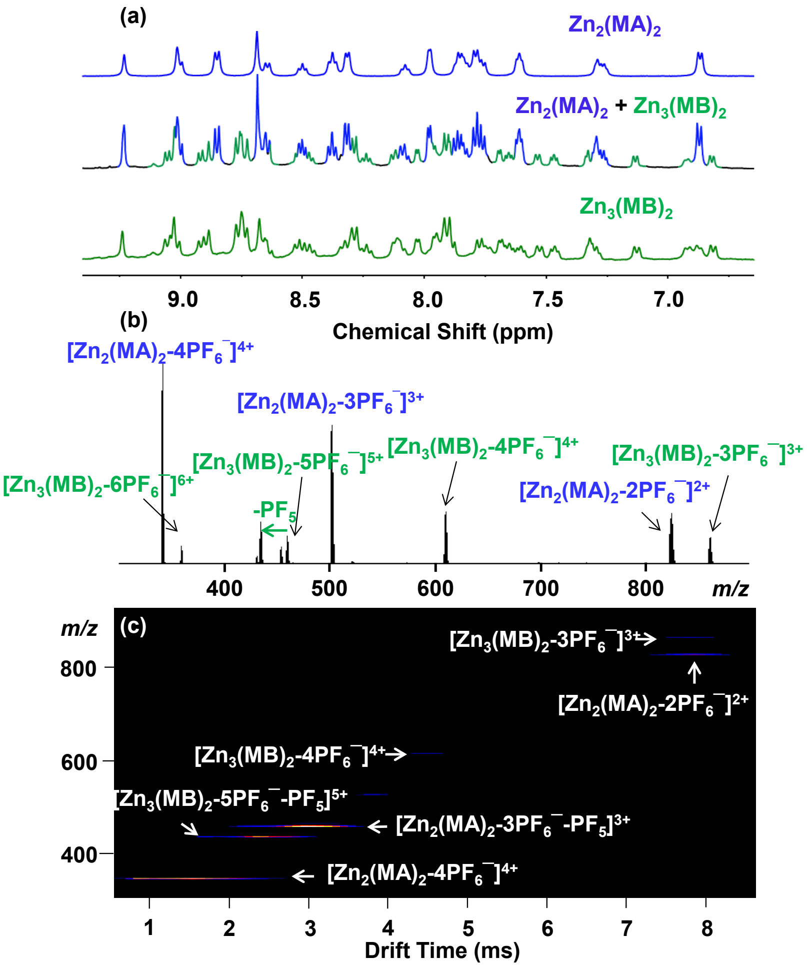

Supplementary Figure 36. (A) ^{1}H NMR spectra (500 MHz, CD_3CN, 300 K) of **$Zn_2(MA)_2$** (top), **$Zn_3(MB)_2$** (bottom) and mixture of both (middle) (3 mg/mL); (B) ESI-MS and (C) TWIM-MS plots (*m/z* vs drift time) of the binary mixture.

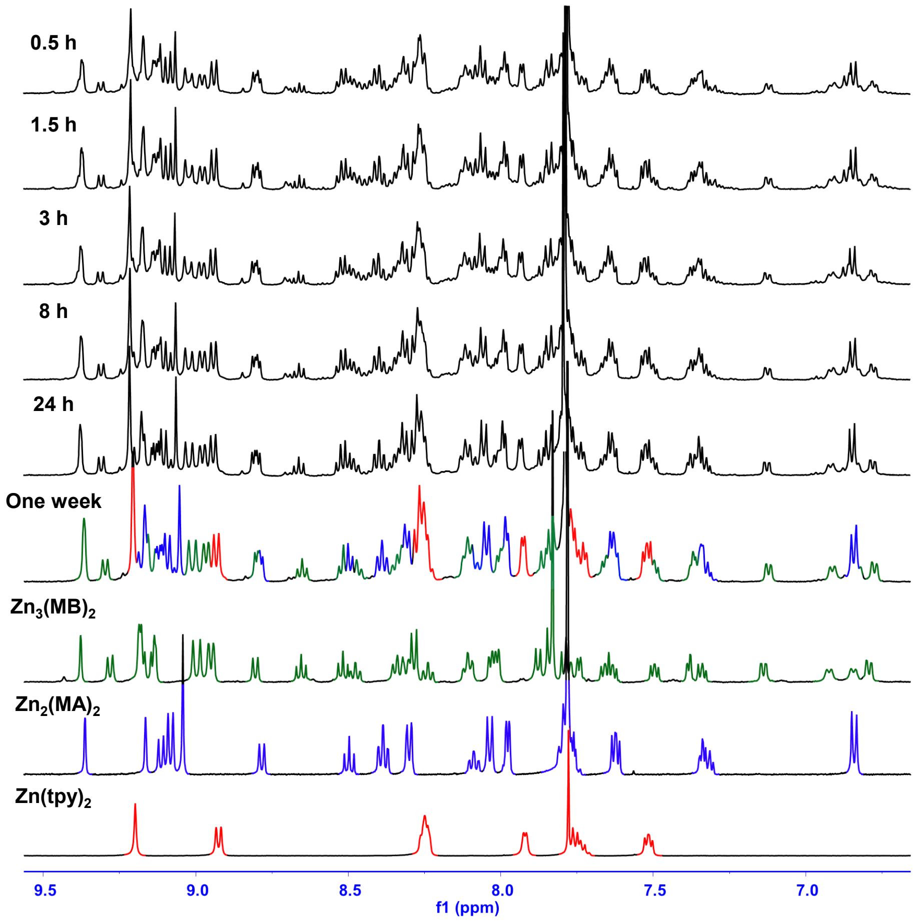

Supplementary Figure 37. ^{1}H NMR spectra (500 MHz, 300 K) of dynamic self-sorting process of **MA**, **MB**, and **tpy** in MeOD/$CDCl_3$ (3 mg/mL).

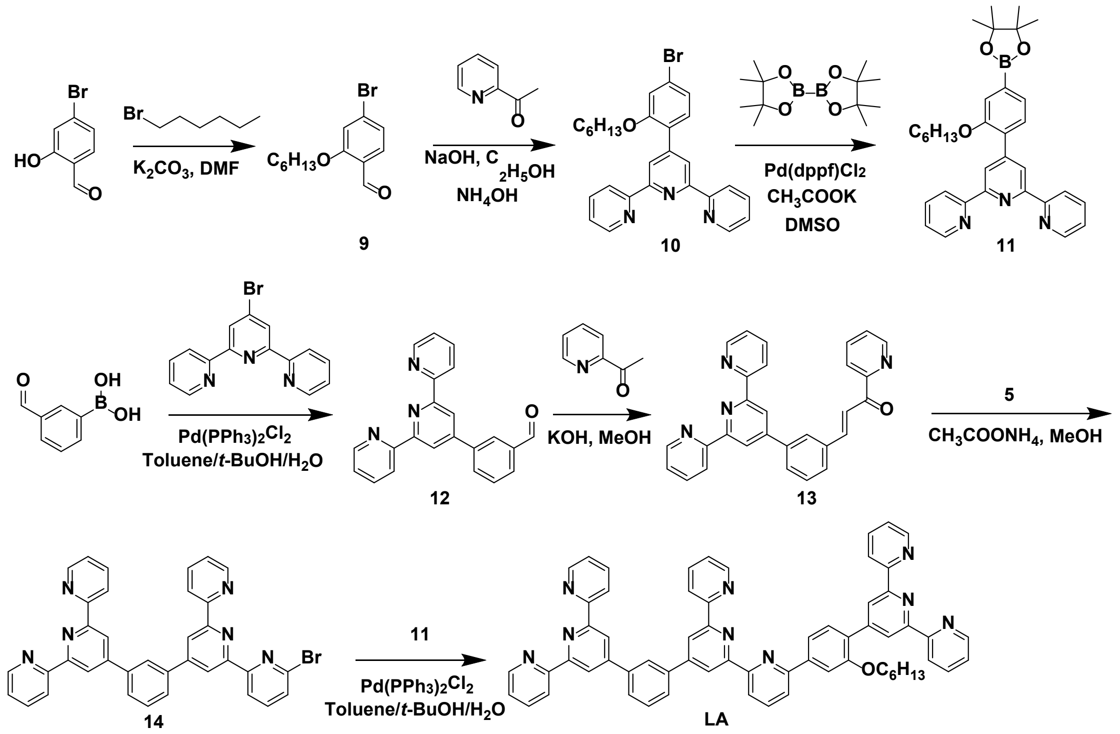

Supplementary Figure 38. Synthesis of ligand **LA.**

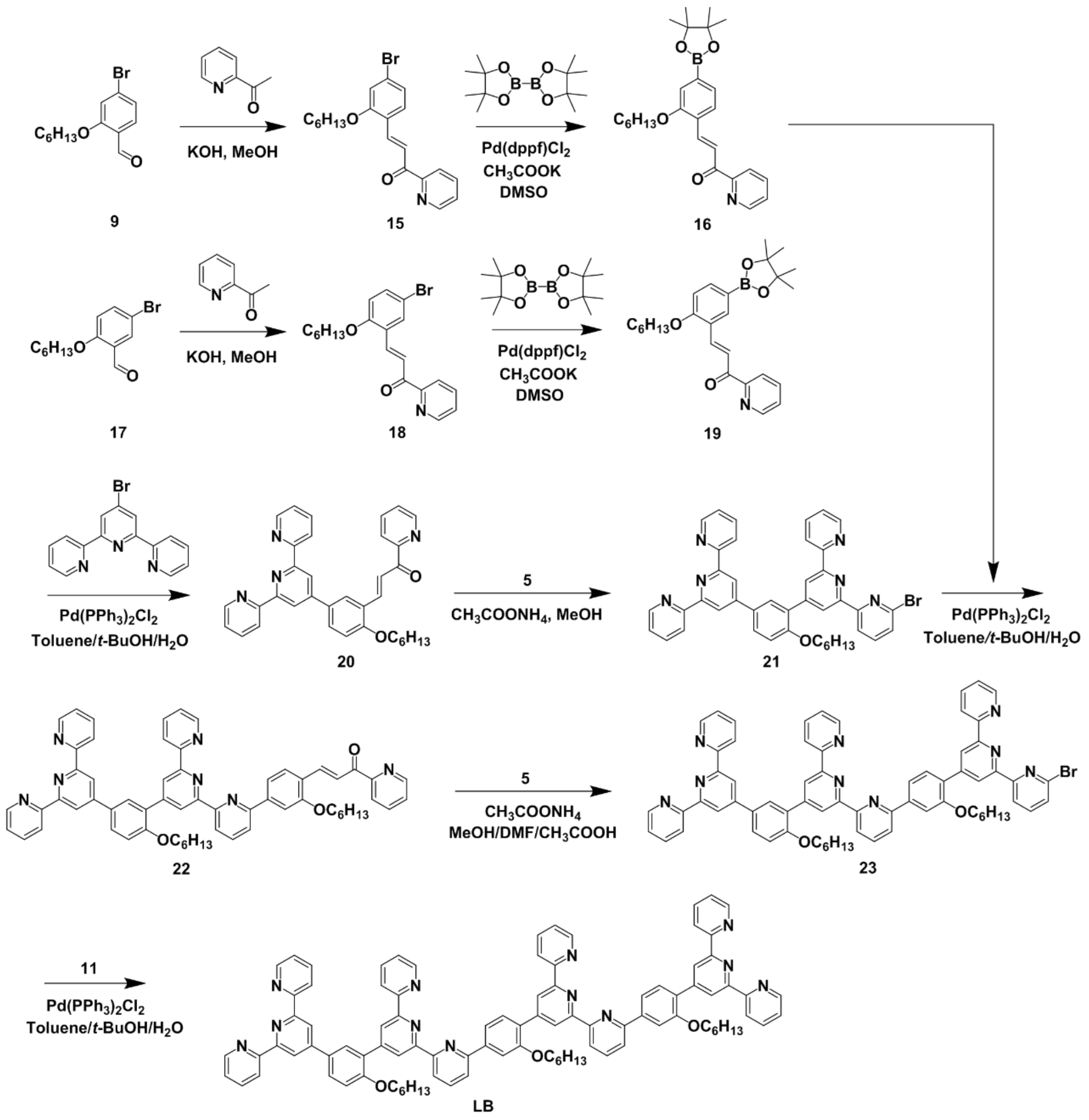

Supplementary Figure 39. Synthesis of ligand **LB**.

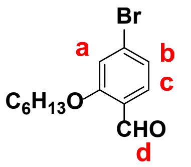

Compound 9: To a solution of 4-bromo-2-hydroxybenzaldehyde (10.0 g, 50 mmol) in DMF (120 mL), 1-bromohexane (16.4 g, 100 mmol) and K_2CO_3 (14 g, 100 mmol) were added and the mixture was heated at 90 °C for 24 h. The reaction mixture was cooled to room temperature, extracted with CH_2Cl_2 and further purified by silica gel column chromatography with CH_2Cl_2: hexane (1:4) to afford the product as white solid (11.2 g, 78%). ^{1}H NMR (400 MHz, $CDCl_3$, 300 K) δ 10.45 (s, 1H, Ph-H^d), 7.71 (d, J = 8.1 Hz, 1H, Ph-H^c), 7.20 – 7.15 (m, 2H, Ph-H^a and Ph-H^b), 4.09 (t, J = 6.4 Hz,

2H), 1.87 (dq, J = 7.9, 6.5 Hz, 2H), 1.51 (dq, J = 11.9, 7.1 Hz, 2H), 1.45 – 1.30 (m, 4H), 1.00 – 0.88 (t, J = 7.0 Hz, 3H). ^{13}C NMR (100 MHz, $CDCl_3$, 300 K) δ 189.13, 161.94, 130.82, 129.66, 124.24, 124.00, 116.40, 69.30, 31.75, 29.19, 25.96, 22.86, 14.32.

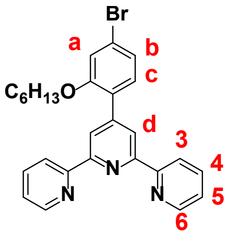

Compound 10: To a solution of NaOH powder (4.2 g, 105 mmol) in 100 mL EtOH, compound **9** (5.0 g, 17.6 mmol) and 2-acetylpyridine (5.3 g, 44 mmol) were added. After stirring at room temperature overnight, aqueous $NH_3 \cdot H_2O$ (75 mL) was added and the mixture was refluxed for 24 h. After cooling down to room temperature, the aqueous phase was extracted using $CHCl_3$ and the organic layer was washed with water for three times. After removing the solvent under reduced pressure, the crude product was purified by column chromatography on silica gel (DCM/EtOH=200/1) to give compound **10** as dark yellow oil (4.5 g, 52%). ^{1}H NMR (400 MHz, $CDCl_3$, 300 K) δ 8.73 – 8.62 (m, 6H, tpy-H^3, tpy-H^6 and tpy-H^d), 7.82 (m, 2H, tpy-H^4), 7.43 (dd, J = 8.1, 1.5 Hz, 1H, Ph-H^c), 7.31 – 7.24 (m, 2H, tpy-H^5), 7.19 (dt, J = 8.1, 1.9 Hz, 1H, Ph-H^b), 7.13 (m, 1H, Ph-H^a), 3.97 (dt, J = 9.4, 4.6 Hz, 2H), 1.75 – 1.63 (m, 2H), 1.44 – 1.36 (m, 2H), 1.21 – 1.06 (m, 4H), 0.74 (t, J = 6.4 Hz, 3H). ^{13}C NMR (100 MHz, $CDCl_3$, 300 K) δ 156.91, 156.28, 155.14, 149.03, 147.29, 136.65, 131.55, 127.23, 123.72, 123.57, 123.31, 121.52, 121.12, 115.59, 68.78, 31.48, 28.98, 25.74, 22.35, 13.95.

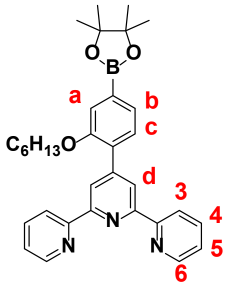

Compound 11: A mixture of compound **10** (974 mg, 2 mmol), *bis*-(pinacolato)diboron (560 mg, 2.2 mmol) and Pd(dppf)Cl_2 (44 mg, 0.06 mmol) was degassed three times in a 50 mL Schlenk flask. After that, 8 mL anhydrous DMSO was added under nitrogen atmosphere and then the mixture was stirred at 85 °C for 12 h. After cooling down to room temperature, the aqueous phase was extracted using $CHCl_3$ and the organic layer was washed with water for three times. After removing the solvent under reduced pressure, the crude product was purified by column chromatography on silica gel (DCM/EtOH=100/1) to give compound **11** as red oil (0.8 g, 75%). ^{1}H NMR (400 MHz, $CDCl_3$, 300 K) δ 8.73 (s, 2H, tpy-H^d), 8.72 – 8.69 (m, 2H, tpy-H^6), 8.69 – 8.64 (m, 2H, tpy-H^3), 7.87 (td, J = 7.7, 1.8 Hz, 2H, tpy-H^4), 7.61 (d, J = 7.5 Hz, 1H, Ph-H^c), 7.53 (d, J = 7.4 Hz, 1H, Ph-H^b), 7.43 (s, 1H, Ph-H^a), 7.33 (dd, J = 7.5, 4.8 Hz, 2H, tpy-H^5), 4.10 (t, J = 6.2 Hz, 2H), 1.77 – 1.67 (m, 2H), 1.40 (m, 14H), 1.22 – 1.08 (m, 4H), 0.75 (t, J = 7.0 Hz, 3H). ^{13}C NMR (100 MHz, $CDCl_3$, 300 K) δ 156.69, 155.98, 155.20, 149.20, 148.50, 136.80, 131.19, 130.13, 127.42, 123.62, 121.97, 121.33, 118.00, 84.06, 68.64, 31.66, 29.33, 25.94, 25.14, 25.03, 22.50, 14.08.

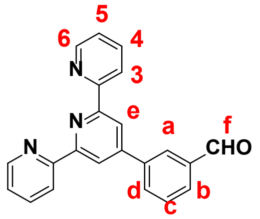

Compound 12: 3-Formyphenylboronic acid (1.4 g, 6 mmol), 4'-bromo-2,2':6',2"-terpyridine (1.56 g, 5 mmol) and $Pd(PPh_3)_2Cl_2$ (210 mg, 0.3 mmol) were added into 100 mL Schlenk flask and degassed three times. Then toluene (30 mL), 1 M Na_2CO_3 (15 mL) and *tert*-butyl alcohol (7 mL) were added under N_2. The resultant mixture was kept at 85 °C for 18 h. After cooling down to the room temperature, the solution was extracted three times with $CHCl_3$ and the solvent was removed under reduced pressure. The crude product was purified by column chromatography on silica gel (DCM/EtOH=150/1) to give compound **12** as white solid (1.23 g, 73%). ^{1}H NMR (400 MHz, $CDCl_3$, 300 K) δ 10.18 (s, 1H, H^f), 8.80 (s,

2H, tpy-H^e), 8.78 – 8.75 (m, 2H, tpy-H^6), 8.71 (d, J = 8.0, 2H, tpy-H^3), 8.43 (s, 1H, Ph-H^a), 8.20 (d, J = 7.6 Hz, 1H, Ph-H^d), 8.01 (d, J = 7.6 Hz, 1H, Ph-H^b), 7.95 (td, J = 7.8, 1.8 Hz, 2H, tpy-H^4), 7.75 (t, J = 7.6 Hz, 1H, Ph-H^c), 7.46 – 7.39 (m, 2H, tpy-H^5). ^{13}C NMR (100 MHz, $CDCl_3$, 300 K) δ 192.25, 156.34, 156.04, 149.31, 148.99, 139.73, 137.17, 137.13, 133.38, 130.05, 129.89, 128.91, 124.20, 121.55, 118.96.

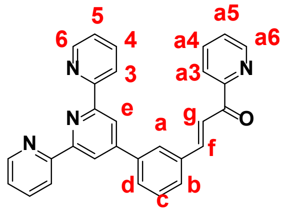

Compound 13: To a solution of compound **12** (3.0 g, 8.8 mmol) and KOH powder (1.0 g, 18 mmol) in 40 mL MeOH, 2-acetylpyridine (1.6 g, 13.2 mmol) was added dropwise. The reaction was kept at 0 °C for 1 h and then removed the ice water bath. and stirred at room temperature overnight. The formed precipitate was filtered and washed by MeOH. After dried under vacuum, compound **13** was obtained as white solid (1.79 g, 46%). ^{1}H NMR (400 MHz, $CDCl_3$, 300 K) δ 8.80 – 8.75 (m, 5H, tpy-H^e, tpy-H^6 and Ph-H^{a6}), 8.72 (dt, J = 8.0, 1.1 Hz, 2H, tpy-H^3), 8.42 (d, J = 16.1 Hz, 1H, H^g), 8.26 – 8.21 (m, 2H, Ph-H^{a3} and Ph-H^a), 8.07 (d, J = 16.0 Hz, 1H, H^f), 7.98 – 7.88 (m, 4H, Ph-H^d, tpy-H^4 and Ph-H^{a4}), 7.85 (d, J = 7.8 Hz, 1H, Ph-H^b), 7.60 (t, J = 7.7 Hz, 1H, Ph-H^c), 7.52 (ddd, J = 7.6, 4.7, 1.3 Hz, 1H, Ph-H^{a5}), 7.39 (ddd, J = 7.5, 4.8, 1.2 Hz, 2H, tpy-H^5). ^{13}C NMR (100 MHz, $CDCl_3$, 300 K) δ 189.57, 156.25, 156.18, 154.30, 149.89, 149.30, 149.01, 144.48, 139.57, 137.16, 137.03, 136.06, 129.68, 129.61, 129.34, 127.79, 127.06, 124.04, 123.10, 121.78, 121.52, 119.14.

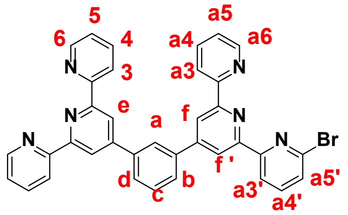

Compound 14: To a 30 mL anhydrous MeOH solution of compound **5** (584 mg, 1.3 mmol), pyridinium salt **5** (787 mg, 1.95 mmol) and NH_4OAc (2.0 g, 26 mmol) were

added. The solution was refluxed for 24 h. After cooling to room temperature, the formed precipitate was filtered and washed by MeOH. After dried under vacuum, compound **14** was obtained as gray solid (0.8 g, 36%). ^{1}H NMR (400 MHz, $CDCl_3$, 300 K) δ 8.82 (m, 3H, tpy-H^{e} and tpy-$H^{f'}$), 8.77 – 8.73 (m, 4H, tpy-H^{f}, tpy-H^{6} and tpy-H^{a6}), 8.71 (d, J = 8.0 Hz, 2H, tpy-H^{3}), 8.66 (dq, J = 8.0, 1.0 Hz, 2H, tpy-H^{a3} and tpy-$H^{a3'}$), 8.32 (t, J = 1.8 Hz, 1H, Ph-H^{a}), 8.00 (d, J = 7.6 Hz, 1H, Ph-H^{d}), 7.96 (d, J = 7.6 Hz, 1H, Ph-H^{b}), 7.90 (m, 3H, tpy-H^{4} and tpy-H^{a4}), 7.74 (t, J = 7.8 Hz, 1H, tpy-$H^{a4'}$), 7.69 (t, J = 7.7 Hz, 1H, Ph-H^{c}), 7.55 (dd, J = 7.8, 0.9 Hz, 1H, tpy-$H^{a5'}$), 7.37 (ddd, J = 7.5, 4.7, 1.1 Hz, 3H, tpy-H^{5} and tpy-H^{a5}). ^{13}C NMR (100 MHz, $CDCl_3$, 300 K) δ 157.76, 156.50, 156.41, 156.36, 156.26, 154.69, 150.73, 150.42, 149.51, 149.49, 149.46, 141.96, 139.91, 139.74, 139.46, 137.20, 129.93, 128.54, 128.49, 128.46, 126.56, 124.28, 124.19, 121.72, 121.67, 120.43, 120.15, 119.95, 119.54.

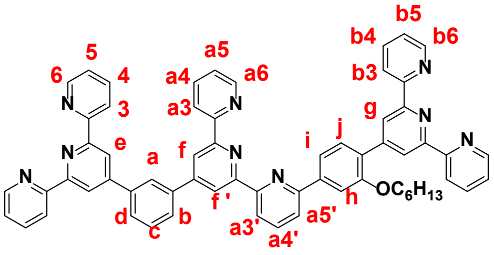

Ligand LA: A mixture of compound **14** (291 mg, 0.5 mmol), compound **11** (385 mg, 0.7 mmol) and $Pd(PPh_3)_2Cl_2$ (39 mg, 0.06 mmol) in 100 mL Schlenk flask was degassed three times. Then toluene (30 mL), 1 M Na_2CO_3 (15 mL) and *tert*-butyl alcohol (7 mL) were added under N_2. The resultant mixture was kept at 85 °C for 24 h. After cooling to the room temperature, the solution was extracted by $CHCl_3$ three times and the solvent was removed under reduced pressure. The crude product was purified by column chromatography on silica gel ($CHCl_3$/EtOH=75/1) to give compound **LA** as white solid (338 mg, 76%). ^{1}H NMR (600 MHz, $CDCl_3$, 300 K) δ 9.07 (d, J = 1.6 Hz, 1H, tpy-$H^{f'}$), 8.86 (d, J = 1.6 Hz, 1H, tpy-H^{f}), 8.86 (s, 2H, tpy-H^{e}), 8.78 – 8.75 (m, 4H, tpy-H^{g}, tpy-H^{a6} and tpy-H^{a3}), 8.73 – 8.67 (m, 9H, tpy-H^{6}, tpy-$H^{a3'}$, tpy-H^{b6}, tpy-H^{b3} and tpy-H^{3}), 8.41 (s, 1H, Ph-H^{a}), 8.02 (m, 4H, Ph-H^{d}, Ph-H^{b}, tpy-$H^{a4'}$ and Ph-H^{h}), 7.95 – 7.90 (m, 2H, tpy-H^{a4} and tpy-$H^{a5'}$), 7.89 (td, J = 7.8, 1.8 Hz, 2H,

tpy-H^{4}), 7.86 – 7.83 (m, 3H, tpy-H^{b4} and Ph-H^{i}), 7.73 – 7.70 (m, 2H, Ph-H^{c} and Ph-H^{j}), 7.39 (ddd, J = 7.4, 4.7, 1.1 Hz, 1H, tpy-H^{a5}), 7.34 (ddd, J = 7.4, 4.7, 1.0 Hz, 2H, tpy-H^{5}), 7.32 (ddd, J = 7.4, 4.8, 1.0 Hz, 2H, tpy-H^{b5}), 4.16 (t, J = 6.2 Hz, 2H), 1.65 – 1.58 (m, 2H), 1.35 – 1.27 (m, 2H), 1.11 – 1.00 (m, 4H), 0.66 (t, J = 7.1 Hz, 3H). ^{13}C NMR (150 MHz, $CDCl_3$, 300 K) δ 157.00, 156.74, 156.41, 156.30, 156.24, 156.17, 155.99, 155.91, 155.29, 150.18, 150.13, 149.30, 149.26, 148.25, 141.11, 139.96, 139.76, 137.84, 137.04, 136.95, 136.83, 131.03, 129.79, 129.04, 128.27, 128.15, 126.25, 124.01, 123.65, 122.00, 121.61, 121.46, 121.35, 120.73, 120.11, 119.55, 119.41, 119.30, 119.22, 110.96, 68.68, 31.67, 29.25, 25.96, 22.48, 14.05. MALDI-TOF MS (*m/z*): Calcd. for $[C_{63}H_{49}N_9O+H]^+$ 948.41. Found: 948.40.

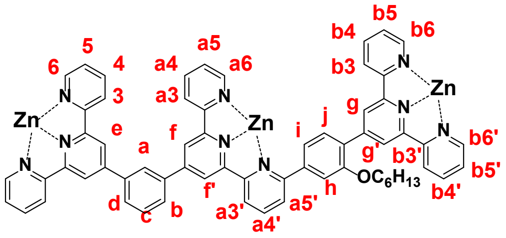

Complex $Zn_9(LA)_6$: To a solution of ligand **LA** (3.4 mg, 3.4 μmol) in $CHCl_3$ (1.0 mL), a solution of $Zn(NO_3)_2$•$6H_2O$ (1.5 mg, 5.1 μmol) in MeOH (3.0 mL) was added, then the mixture was kept in a 50 °C for 12 h. After cooling to room temperature, excess NH_4PF_6 (around 100 mg) was added to generate a white precipitate (4.3 mg, 86%). ^{1}H NMR (600 MHz, CD_3CN, 300 K) δ 9.43 (s, 1H, tpy-$H^{f'}$), 9.27 (d, J = 9.0 Hz, 2H, tpy-H^{e}), 9.21 (d, J = 5.9 Hz, 1H, tpy-H^{f}), 9.11 (d, J = 7.9 Hz, 1H, tpy-$H^{a3'}$), 9.02-8.95 (m, 2H, Ph-H^{a} and tpy-$H^{g'}$), 8.90 (t, J = 7.9 Hz, 2H, tpy-H^{3}), 8.74-8.73 (m, 3H, tpy-H^{a3}, tpy-H^{b3} and tpy-H^{g}), 8.65 – 8.55 (m, 3H, tpy-$H^{b3'}$, Ph-H^{b} and Ph-H^{d}), 8.52 – 8.46 (m, 2H, tpy-$H^{a4'}$ and tpy-$H^{b4'}$), 8.28 (m, J = 8.0 Hz, 3H, tpy-H^{b4} and tpy-H^{4}), 8.19-8.16 (m, J = 15.7, 11.6 Hz, 2H, Ph-H^{c} and tpy-$H^{b6'}$), 8.10 (d, J = 8.3 Hz, 1H, tpy-H^{a4}), 8.01 (d, J = 5.1 Hz, 2H, tpy-H^{6}), 7.95 (s, 1H, tpy-H^{b6}), 7.75 (s, 1H, tpy-$H^{a5'}$), 7.66 (s, 1H, tpy-$H^{b5'}$), 7.59 (s, 1H, tpy-H^{b5}), 7.53 (t, J = 6.2 Hz, 2H, tpy-H^{5}), 7.38 (d, J = 9.6 Hz, 1H, Ph-H^{j}), 7.28 (s, 2H, tpy-H^{a6} and tpy-H^{a5}), 6.41 (s, 2H, Ph-H^{h}

and Ph-H^i), 3.72-3.55 (m, 2H), 1.90 (s, 2H), 1.46 (s, 2H), 1.30 (s, 2H), 1.16 (s, 2H), 0.77 (s, 3H). ^{13}C NMR (150 MHz, CD_3CN, 300 K) δ 160.81, 156.61, 156.48, 152.99, 152.22, 151.44, 151.07, 150.20, 149.99, 149.60, 149.18, 148.91, 148.70, 148.11, 147.85, 142.83, 142.51, 142.38, 141.98, 138.90, 138.82, 138.60, 138.45, 131.96, 131.66, 131.47, 129.44, 129.27, 129.08, 128.75, 128.52, 126.17, 124.37, 124.19, 123.90, 123.52, 123.41, 123.25, 123.10, 121.43, 113.25, 70.29, 32.29, 29.61, 26.63, 23.29, 14.23. ESI MS (*m/z*): 1124.5 $[M-7PF_6^-]^{7+}$ (calcd *m/z*: 1124.5), 965.7 $[M-8PF_6^-]^{8+}$ (calcd *m/z*: 965.7), 842.4 $[M-9PF_6^-]^{9+}$ (calcd *m/z*: 842.4), 743.6 $[M-10PF_6^-]^{10+}$ (calcd *m/z*: 743.6), 662.9 $[M-11PF_6^-]^{11+}$ (calcd *m/z*: 662.9), 595.6 $[M-12PF_6^-]^{12+}$ (calcd *m/z*: 595.6), 538.6 $[M-13PF_6^-]^{13+}$ (calcd *m/z*: 538.6), 489.7 $[M-14PF_6^-]^{14+}$ (calcd *m/z*: 489.7).

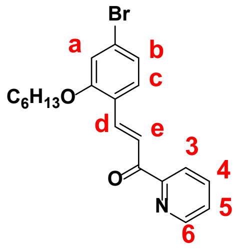

Compound 15: To a solution of compound **9** (6.0 g, 21 mmol) and KOH powder (2.0 g, 36 mmol) in 60 mL MeOH at 0 °C, 2-acetylpyridine (3.1 g, 26 mmol) was added dropwise. The reaction was kept at 0 ºC for 1 h and then removed the ice water bath. After 12 h, the reaction mixture was extracted with $CHCl_3$ three times. After removing the solvent under reduced pressure, the crude product was purified by column chromatography on silica gel (DCM) to give compound **15** as dark green oil (6.0 g, 73%). ^{1}H NMR (400 MHz, $CDCl_3$, 300 K) δ 8.73 (d, J = 4.4 Hz, 1H, Ph-H^6), 8.40 (d, J = 16.4 Hz, 1H, H^e), 8.23 – 8.16 (m, 2H, H^d and Ph-H^3), 7.88 (td, J = 7.8, 1.5 Hz, 1H, Ph-H^4), 7.62 (d, J = 8.2 Hz, 1H, Ph-H^c), 7.48 (ddt, J = 7.1, 4.8, 1.1 Hz, 1H, Ph-H^5), 7.12 (dd, J = 8.3, 1.8 Hz, 1H, Ph-H^b), 7.07 (d, J = 1.7 Hz, 1H, Ph-H^a), 4.05 (t, J = 6.4 Hz, 2H), 1.97 – 1.83 (m, 2H), 1.54 (m, 2H), 1.38 (m, 4H), 0.91 (t, J = 6.9 Hz, 3H). ^{13}C NMR (100 MHz, $CDCl_3$, 300 K) δ 189.84, 158.95, 154.49, 148.91, 139.17, 137.07, 130.36, 126.88, 125.56, 123.78, 123.35, 122.98, 121.71, 115.71, 69.08, 31.67, 29.14, 25.90, 22.69, 14.18.

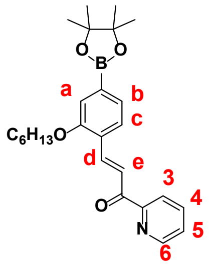

Compound 16: A Schlenk flask was charged with compound **15** (3.5 g, 9 mmol), *bis*-(pinacolato)diboron (2.6 g, 10 mmol), Pd(dppf)Cl_2 (200 mg, 0.3 mmol) and potassium acetate (2.7 g, 27 mmol). After that, the flask was degassed three times and added 32 mL anhydrous DMSO. The mixture was stirred at 85 °C for 14 h and then cooled to room temperature. The solution was extracted three times with $CHCl_3$ and then the solvent was removed under reduced pressure. The crude product was purified by column chromatography on silica gel (DCM) to give compound **16** as dark green oil (3.1 g, 77%). ^{1}H NMR (400 MHz, $CDCl_3$, 300 K) δ 8.76 (ddd, J = 4.7, 1.8, 0.9 Hz, 1H, Ph-H^6), 8.42 (d, J = 16.2 Hz, 1H, H^e), 8.33 (d, J = 16.2 Hz, 1H, H^d), 8.20 (dt, J = 7.9, 1.1 Hz, 1H, Ph-H^3), 7.89 (td, J = 7.7, 1.8 Hz, 1H, Ph-H^4), 7.78 (d, J = 7.6 Hz, 1H, Ph-H^c), 7.49 (ddd, J = 7.6, 4.8, 1.3 Hz, 1H, Ph-H^5), 7.45 – 7.40 (d, J = 7.6 Hz, 1H, Ph-H^b), 7.35 (s, Ph-H^a), 4.14 (t, J = 6.5 Hz, 2H), 1.92 (m, 2H), 1.58 (m, 2H), 1.38 (m, 16H), 0.92 (t, J = 7.2 Hz, 3H). ^{13}C NMR (100 MHz, $CDCl_3$, 300 K) δ 190.24, 158.24, 154.89, 149.15, 140.50, 137.27, 128.79, 127.12, 127.02, 123.22, 122.32, 118.01, 84.38, 83.54, 68.92, 53.79, 31.96, 29.61, 26.21, 25.22, 22.95, 14.42.

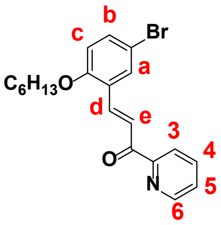

Compound 18: To a solution of compound **17**[5] (5.4 g, 19 mmol) and KOH powder (2.1 g, 36 mmol) in 60 mL MeOH at 0 °C, 2-acetylpyridine (3.45 g, 29 mmol) was added dropwise. The reaction was kept at 0 ºC for 1 h and then removed the ice water bath. The reaction was stopped after 12 h and extracted with $CHCl_3$ three times. After

removing the solvent under reduced pressure, the crude product was purified by column chromatography on silica gel (DCM) to give compound **18** as green oil (4.4 g, 60%). ^{1}H NMR (400 MHz, $CDCl_3$, 300 K) δ 8.79 – 8.71 (m, 1H, Ph-H^6), 8.34 (d, J = 16.2 Hz, 1H, H^e), 8.22 (d, J = 10.3 Hz, 1H, H^d), 8.19 (d, J = 1.8 Hz, 1H, Ph-H^3), 7.92 – 7.85 (m, 2H, Ph-H^4 and Ph-H^a), 7.50 (ddd, J = 7.5, 4.8, 1.1 Hz, 1H, Ph-H^5), 7.43 (dd, J = 8.8, 2.5 Hz, 1H, Ph-H^b), 6.81 (d, J = 8.8 Hz, 1H, Ph-H^c), 4.03 (t, J = 6.5 Hz, 2H), 1.95 – 1.81 (m, 2H), 1.57 – 1.44 (m, 2H), 1.41 – 1.30 (m, 4H), 0.91 (t, J = 7.0 Hz, 3H). ^{13}C NMR (100 MHz, $CDCl_3$, 300 K) δ 189.86, 157.73, 154.58, 149.17, 138.71, 137.30, 134.37, 131.57, 127.17, 126.53, 123.21, 122.44, 114.13, 113.09, 69.25, 31.89, 29.40, 26.11, 22.90, 14.38.

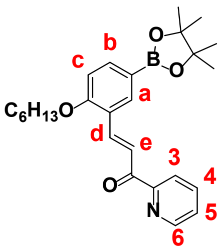

Compound 19: Compound **18** (5.3 g, 13.6 mmol), *bis*-(pinacolato)diboron (3.8 g, 15 mmol), Pd(dppf)Cl_2 (400 mg, 0.6 mmol) and potassium acetate (5.9 g, 60 mmol) were added into 200 mL Schlenk flask. After that, the flask was degassed three times and added 50 mL anhydrous DMSO. The mixture was stirred at 85 ^{o}C for 14 h and then cooled to room temperature. The solution was extracted three times with $CHCl_3$ and then the solvent was removed under reduced pressure. The crude product was purified by column chromatography on silica gel (DCM) to give compound **19** as dark green oil (4.8 g, 81%). ^{1}H NMR (400 MHz, $CDCl_3$, 300 K) δ 8.76 (d, J = 4.6 Hz, 1H, Ph-H^6), 8.40 (d, J = 16.2 Hz, 1H, H^e), 8.34 (d, J = 16.2 Hz, 1H, H^d), 8.23 – 8.17 (m, 2H, Ph-H^a and Ph-H^3), 7.88 (ddd, J = 7.7, 3.2, 1.4 Hz, 1H, Ph-H^4), 7.82 (dd, J = 8.3, 1.3 Hz, 1H, Ph-H^b), 7.50 – 7.46 (m, 1H, Ph-H^5), 6.93 (d, J = 8.4, 1H, Ph-H^c), 4.10 (t, J = 6.5 Hz, 2H), 1.98 – 1.85 (m, 2H), 1.61 – 1.48 (m, 2H), 1.34 (d, J = 19.1 Hz, 16H), 0.91 (d, J = 6.4 Hz, 3H). ^{13}C NMR (100 MHz, $CDCl_3$, 300 K) δ 190.07, 161.04, 154.77, 148.87, 140.48, 138.86, 137.01, 136.33, 126.72, 123.78, 122.99, 121.45,

111.38, 83.89, 68.64, 31.71, 29.23, 25.93, 24.99, 24.66, 22.69, 14.17.

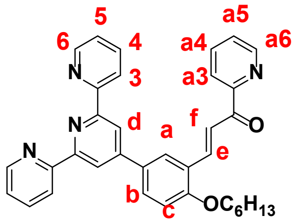

Compound 20: A mixture of 4'-bromo-2,2':6',2''-terpyridine (936 mg, 3 mmol), compound **19** (1.82 g, 4.2 mmol) and $Pd(PPh_3)_2Cl_2$ (235 mg, 0.3 mmol) was degassed three times. Then toluene (40 mL), 1 M Na_2CO_3 (20 mL) and *tert*-butyl alcohol (10 mL) were added under N_2. The resultant mixture was kept at 85 °C for 48 h. After cooling down to the room temperature, the solution was extracted three times with $CHCl_3$, and then the solvent was removed under reduced pressure. The crude product was purified by column chromatography on silica gel (DCM/EtOH=150/1) to give compound **20** as green solid (0.8 g, 51%). ^1H NMR (400 MHz, $CDCl_3$, 300 K) δ 8.75 – 8.69 (m, 5H, tpy-H^6, tpy-H^d and Ph-H^{a6}), 8.67 – 8.63 (m, 2H, tpy-H^3), 8.49 (d, J = 16.2 Hz, 1H, H^f), 8.35 (d, J = 16.2 Hz, 1H, H^e), 8.23 (d, J = 1.9 Hz, 1H, Ph-H^a), 8.20 (d, J = 7.8 Hz, 1H, Ph-H^{a3}), 7.88 – 1.81 (m, 4H, Ph-H^b, tpy-H^4 and Ph-H^{a4}), 7.47 – 7.42 (m, 1H, Ph-H^{a5}), 7.35 – 7.28 (m, 2H, tpy-H^5), 7.04 – 6.98 (m, 1H, Ph-H^c), 4.09 (t, J = 6.3 Hz, 2H), 1.95 – 1.87 (m, 2H), 1.50 – 1.52 (m, 2H), 1.41 – 1.31 (m, 4H), 0.97 – 0.85 (m, 3H). ^{13}C NMR (100 MHz, $CDCl_3$, 300 K) δ 190.14, 159.52, 156.57, 156.08, 154.74, 149.54, 149.31, 149.05, 140.25, 137.12, 137.06, 130.95, 130.92, 128.49, 126.90, 124.86, 124.03, 123.08, 122.43, 121.59, 118.56, 112.61, 69.06, 31.89, 29.43, 26.11, 22.85, 14.34.

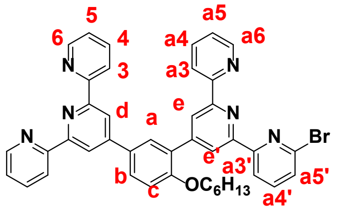

Compound 21: To a 30 mL anhydrous MeOH solution of compound **20** (820 mg, 1.5 mmol), pyridinium salt **5** (888 mg, 2.2 mmol) and NH_4OAc (2.3 g, 30 mmol) were

added. The solution was refluxed for 24 h, and then cooled to the room temperature. After that, the solution was extracted three times with $CHCl_3$ and the solvent was removed under reduced pressure. The crude product was purified by column chromatography on silica gel (DCM/EtOH=100/1) to give compound **21** as green solid (0.55 g, 51%). ^{1}H NMR (400 MHz, $CDCl_3$, 300 K) δ 8.76 – 8.74 (m, 3H, tpy-H^{d} and tpy-$H^{e'}$), 8.74 – 8.69 (m, 4H, tpy-H^{6}, tpy-H^{e} and tpy-H^{a6}), 8.69 – 8.62 (m, 4H, tpy-H^{3}, tpy-H^{a3} and tpy-$H^{a3'}$), 8.06 (d, J = 2.3 Hz, 1H, Ph-H^{a}), 7.96 (dd, J = 8.6, 2.3 Hz, 1H, Ph-H^{b}), 7.91 – 7.84 (m, 3H, tpy-H^{4} and tpy-H^{a4}), 7.72 (t, J = 7.8 Hz, 1H, tpy-$H^{a4'}$), 7.51 (d, J = 7.8 Hz, 1H, tpy-$H^{a5'}$), 7.36 – 7.31 (m, 3H, tpy-H^{a5} and tpy-H^{5}), 7.13 (d, J = 8.6 Hz, 1H, Ph-H^{c}), 4.10 (t, J = 6.3 Hz, 2H), 1.83 – 1.73 (m, 2H), 1.48 – 1.38 (m, 2H), 1.28 – 1.09 (m, 4H), 0.74 (t, J = 7.2 Hz, 3H). ^{13}C NMR (100 MHz, $CDCl_3$, 300 K) δ 158.03, 157.56, 156.61, 156.54, 156.18, 155.66, 153.79, 149.99, 149.47, 149.42, 148.60, 141.92, 139.36, 137.22, 137.11, 131.23, 129.57, 129.45, 129.08, 128.16, 124.11, 124.02, 122.85, 122.78, 121.75, 121.52, 120.24, 118.85, 112.82, 69.05, 31.85, 29.41, 26.10, 22.70, 14.30.

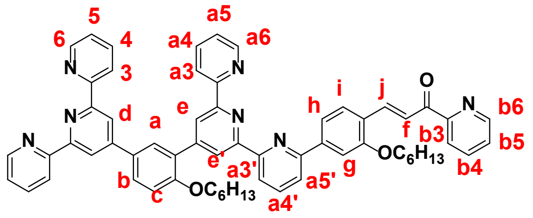

Compound 22: Compound **21** (1.76 g, 2.45 mmol), compound **16** (2.1 g, 4.9 mmol) and $Pd(PPh_3)_2Cl_2$ (140 mg, 0.2 mmol) were added into 100 mL Schlenk flask and was degassed three times. Then toluene (30 mL), 1 M Na_2CO_3 (15 mL) and *tert*-butyl alcohol (7 mL) were added under N_2. The resultant mixture was kept at 85 ^{o}C for 18 h. After cooling down to the room temperature, the solution was extracted three times with $CHCl_3$, and the solvent was removed under reduced pressure. The crude product was purified by column chromatography on silica gel ($CHCl_3$/EtOH=100/1) to give compound **22** as dark green solid (1.38 g, 60%). ^{1}H NMR (400 MHz, $CDCl_3$, 300 K) δ 8.96 (s, 1H, tpy-$H^{e'}$), 8.81 (s, 1H, tpy-H^{e}), 8.79 (s, 2H, tpy-H^{d}), 8.72 – 8.62 (m, 8H, tpy-H^{3}, tpy-H^{6}, tpy-H^{a3}, tpy-H^{a6}, tpy-$H^{a3'}$ and Ph-H^{b6}), 8.40 (d, J = 16.2 Hz, 1H, H^{f}),

8.31 (d, J = 16.2 Hz, 1H, H^{j}), 8.20 – 8.14 (m, 2H, Ph-H^{b3} and Ph-H^{a}), 7.99 (dd, J = 8.5, 1.9 Hz, 1H, Ph-H^{b}), 7.92 (t, J = 7.8 Hz, 1H, tpy-$H^{a4'}$), 7.88 – 7.77 (m, 7H, tpy-H^{4}, tpy-H^{a4}, Ph-H^{b4}, tpy-$H^{a5'}$, Ph-H^{h} and Ph-H^{g}), 7.73 (d, J = 8.0 Hz, 1H, Ph-H^{i}), 7.46 – 7.40 (m, 1H, tpy-H^{a5}), 7.34 – 7.26 (m, 3H, tpy-H^{5} and Ph-H^{b5}), 7.17 (d, J = 8.6 Hz, 1H, Ph-H^{c}), 4.16 – 4.04 (m, 4H), 1.78 – 1.66 (m, 4H), 1.43 – 1.33 (m, 4H), 1.28 – 1.14 (m, 4H), 1.14 – 0.96 (m, 4H), 0.79 (t, J = 6.5 Hz, 3H), 0.65 (t, J = 7.0 Hz, 3H). ^{13}C NMR (100 MHz, $CDCl_3$, 300 K) δ 189.88, 158.96, 157.34, 156.47, 156.23, 156.06, 155.97, 155.34, 155.31, 155.27, 155.21, 155.16, 154.64, 149.47, 149.14, 148.84, 147.94, 142.75, 139.94, 137.61, 136.90, 136.85, 136.77, 131.01, 129.65, 129.26, 129.18, 129.08, 126.66, 124.72, 123.85, 123.65, 122.83, 122.24, 122.12, 121.47, 121.37, 121.29, 120.42, 120.14, 119.03, 118.33, 112.71, 110.50, 68.90, 68.60, 31.64, 31.56, 29.26, 29.19, 25.90, 22.61, 22.35, 14.11, 13.99.

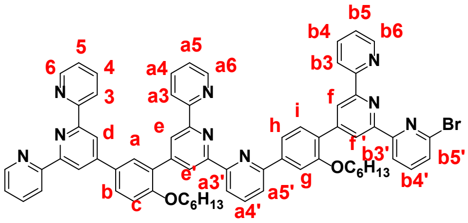

Compound 23: A mixture of NH_4OAc (2.2 g, 29 mmol), pyridinium salt **5** (1.2 g, 2.9 mmol) and compound **22** (1.38 g, 1.45 mmol) was added into 100 mL round flask. Then 20 mL anhydrous MeOH, 40 mL anhydrous DMF and 10 mL AcOH were added. The solution was refluxed for 24 h, and then cooled to the room temperature. The solution was extracted three times with $CHCl_3$, and then removed the solvent under reduced pressure. The crude product was purified by column chromatography on silica gel ($CHCl_3$/EtOH=75/1) and washed by MeOH to afford the product as light green solid (574 mg, 35%). ^{1}H NMR (400 MHz, $CDCl_3$, 300 K) δ 9.02 (d, J = 1.5 Hz, 1H, tpy-$H^{e'}$), 8.82 (d, J = 1.5 Hz, 1H, tpy-H^{e}), 8.81 (s, 2H, tpy-H^{d}), 8.79 – 8.62 (m, 12H, tpy-H^{6}, tpy-H^{3}, tpy-H^{a6}, tpy-H^{a3}, tpy-H^{b6}, tpy-H^{b3}, tpy-$H^{a3'}$, tpy-$H^{b3'}$, tpy-H^{f} and tpy-$H^{f'}$), 8.17 (d, J = 2.3 Hz, 1H, Ph-H^{a}), 8.04 – 7.98 (m, 3H, Ph-H^{b}, Ph-H^{g} and tpy-$H^{a4'}$), 7.96 – 7.84 (m, 6H, tpy-H^{4}, tpy-H^{a4}, tpy-H^{b4} and tpy-$H^{a5'}$ and Ph-H^{h}), 7.76 – 7.68 (m, 2H, Ph-H^{i} and tpy-$H^{b4'}$), 7.52 (d, J = 7.8 Hz, 1H, tpy-$H^{b5'}$), 7.40 – 7.31 (m,

4H, tpy-H^5, tpy-H^{a5} and tpy-H^{b5}), 7.20 (d, J = 8.7 Hz, 1H, Ph-H^c), 4.15 (t, J = 6.2 Hz, 4H), 1.83 – 1.72 (m, 2H), 1.71 – 1.60 (m, 2H), 1.49 – 1.37 (m, 2H), 1.36 – 1.25 (m, 2H), 1.21 – 0.97 (m, 8H), 0.73 – 0.63 (m, 6H). ^{13}C NMR (100 MHz, $CDCl_3$, 300 K) δ 158.04, 157.56, 157.06, 156.81, 156.60, 156.52, 156.31, 156.18, 155.97, 155.67, 155.54, 155.51, 155.41, 153.68, 141.83, 141.55, 139.25, 137.83, 137.05, 137.01, 136.96, 133.31, 133.29, 131.26, 130.96, 129.75, 129.59, 129.48, 129.25, 129.15, 129.05, 128.67, 128.01, 124.03, 123.87, 123.83, 122.52, 122.50, 122.48, 122.39, 121.59, 121.53, 121.39, 120.58, 120.14, 120.08, 119.99, 119.50, 118.63, 112.90, 111.07, 69.13, 68.79, 31.78, 31.72, 29.48, 29.30, 26.11, 26.01, 22.56, 22.55, 14.19.

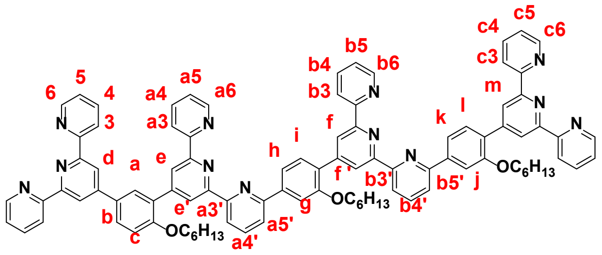

Ligand LB: Compound **23** (574 mg, 0.5 mmol), compound **11** (535 mg, 1 mmol) and $Pd(PPh_3)_2Cl_2$ (56 mg, 0.08 mmol) were added into 100 mL Schlenk flask and was degassed three times. Then toluene (30 mL), 1 M Na_2CO_3 (15 mL) and *tert*-butyl alcohol (7 mL) were added under N_2. The resultant mixture was kept at 85 °C for 24 h. The reaction mixture was cooled to the room temperature, extracted with $CHCl_3$, and removed the solvent under reduced pressure. The crude product was purified by column chromatography on silica gel ($CHCl_3$/EtOH=60/1) to give compound **LB** as white solid (269 mg, 37%). ^{1}H NMR (600 MHz, $CDCl_3$, 300 K) δ 9.02 (d, J = 1.6 Hz, 1H, tpy-$H^{e'}$), 8.98 (d, J = 1.6 Hz, 1H, tpy-$H^{f'}$), 8.81 (d, J = 1.6 Hz, 2H, tpy-H^e and tpy-H^f), 8.79 (s, 2H, tpy-H^m), 8.77 – 8.74 (m, 3H, tpy-H^d and tpy-H^{a3}), 8.72 (m, 4H, tpy-$H^{a3'}$, tpy-H^{b3}, tpy-H^{b6} and tpy-H^{a6}), 8.71 – 8.66 (m, 7H, tpy-H^6, tpy-H^3, tpy-H^{c6} and tpy-$H^{b3'}$), 8.64 (d, J = 7.9 Hz, 2H, tpy-H^{c3}), 8.14 (d, J = 2.4 Hz, 1H, Ph-H^a), 7.99 (dt, J = 11.4, 7.7 Hz, 3H, Ph-H^j, tpy-$H^{a4'}$ and tpy-$H^{b4'}$), 7.93 (d, J = 1.4 Hz, 1H, Ph-H^g), 7.93 – 7.85 (m, 8H, tpy-H^{a4}, tpy-H^{b4}, tpy-H^4, tpy-$H^{a5'}$, tpy-$H^{b5'}$, Ph-H^b and Ph-H^h), 7.84 (dd, J = 7.9, 1.5 Hz, 1H, Ph-H^k), 7.81 (td, J = 7.7, 1.8 Hz, 2H, tpy-H^{c4}), 7.73 (d, J = 7.9 Hz, 1H, Ph-H^i), 7.71 (d, J = 7.9 Hz, 1H, Ph-H^l), 7.37 – 7.33 (m, 2H, tpy-H^{a5} and

tpy-H^{b5}), 7.32 (ddd, J = 7.4, 4.7, 1.2 Hz, 2H, tpy-H^{5}), 7.30 – 7.27 (m, 2H, tpy-H^{c5}), 7.13 (d, J = 8.7 Hz, 1H, Ph-H^{c}), 4.15 (dd, J = 14.4, 6.3 Hz, 4H), 4.11 (t, J = 6.3 Hz, 2H), 1.78 – 1.70 (m, 4H), 1.65 – 1.58 (m, 2H), 1.44 – 1.36 (m, 4H), 1.30 – 1.24 (m, 2H), 1.17 – 1.09 (m, 4H), 1.09 – 1.02 (m, 4H), 1.01 – 0.95 (m, 2H), 0.95 – 0.90 (m, 2H), 0.67 (td, J = 7.2, 1.5 Hz, 6H), 0.58 (t, J = 7.2 Hz, 3H). ^{13}C NMR (150 MHz, $CDCl_3$, 300 K) δ 157.29, 156.89, 156.82, 156.66, 156.59, 156.25, 156.15, 156.11, 155.90, 155.80, 155.75, 155.26, 155.16, 155.11, 149.57, 149.10, 148.12, 148.05, 147.98, 141.27, 141.14, 137.53, 136.73, 136.67, 131.06, 130.80, 130.71, 129.28, 129.22, 129.07, 129.00, 128.86, 123.72, 123.50, 122.20, 122.14, 122.06, 121.98, 121.80, 121.29, 121.20, 120.30, 120.22, 119.86, 119.79, 119.43, 119.29, 118.39, 112.70, 111.10, 110.93, 68.88, 68.74, 31.49, 29.19, 25.83, 22.23, 13.90. MALDI-TOF MS (*m/z*): Calcd. for $[C_{96}H_{86}N_{12}O_3+H]^+$ 1455.70. Found: 1455.70.

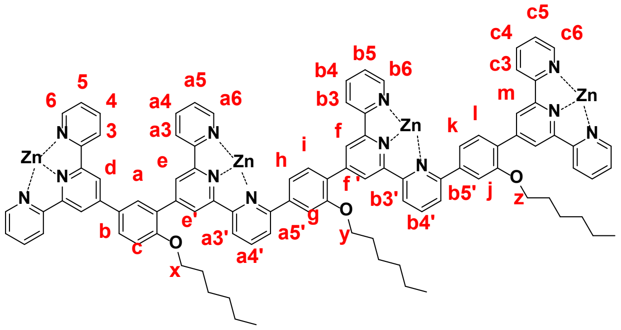

Complex $Zn_{12}(LB)_6$: To a solution of ligand **LB** (4.6 mg, 3.2 μmol) in $CHCl_3$ (1.0 mL), a solution of $Zn(NO_3)_2$•$6H_2O$ (1.9 mg, 6.4 μmol) in MeOH (3.0 mL) was added, then the mixture was kept in a 50 °C for 12 h. After cooling to room temperature, excess NH_4PF_6 (around 100 mg) was added to generate a white precipitate (5.8 mg, 84%). ^{1}H NMR (600 MHz, CD_3CN, 300 K) δ 9.41 (s, 1H, tpy-$H^{e'}$), 9.23 (s, 4H, tpy-H^{d}, tpy-H^{e} and tpy-H^{f}), 9.03 – 8.81 (m, 8H, tpy-$H^{m'}$, tpy-H^{m}, tpy-$H^{a3'}$, tpy-H^{3}, tpy-$H^{b3'}$, tpy-$H^{b3}$and Ph-H^{a}), 8.73 – 8.49 (m, 7H, tpy-H^{c3}, tpy-H^{a3}, tpy-H^{f}, Ph-H^{b}, tpy-$H^{b4'}$ and tpy-$H^{a4'}$), 8.28 (d, 4H, tpy-H^{4} and tpy-H^{c4}), 8.17 – 8.13 (m, J = 23.0 Hz, 3H, tpy-H^{b4} tpy-H^{b6} and tpy-H^{a4}), 7.99 – 7.91 (m, J = 24.3 Hz, 5H, tpy-H^{c6}, tpy-H^{6} and Ph-H^{l}), 7.77-7.73 (m, J = 22.6 Hz, 3H, tpy-$H^{a5'}$, tpy-$H^{b5'}$ and Ph-H^{c}), 7.61 (s, 1H, tpy-H^{i}), 7.53 (d, 4H, tpy-H^{5}, tpy-H^{c5}), 7.42 (s, 1H, Ph-H^{b5}), 7.31 (s, 2H, tpy-H^{a6} and tpy-H^{a5}), 6.78 (s, 1H, Ph-H^{h}), 6.46 (s, 1H, Ph-H^{k}), 6.38 – 6.35 (d, J = 44.1 Hz, 2H,

Ph-H^g and Ph-H^j), 4.49 (s, 2H, H^x), 4.08 (d, J = 47.4 Hz, 2H, H^z), 3.72 (d, J = 78.9 Hz, 2H, H^y), 1.18-0.70 (m, 33H). ^{13}C NMR (150 MHz, CD_3CN, 300 K) δ 160.76, 159.99, 159.32, 156.55, 151.54, 150.89, 150.42, 149.86, 148.99, 147.90, 142.43, 142.25, 142.03, 128.56, 127.54, 126.84, 125.89, 125.79, 124.80, 124.54, 124.20, 123.91, 123.64, 123.01, 122.11, 121.82, 70.36, 32.33, 29.99, 29.73, 29.56, 26.78, 26.48, 23.23, 14.17. ESI-MS (*m/z*): 1480.0 $[M-8PF_6^-]^{8+}$ (calcd *m/z*: 1480.0), 1299.2 $[M-9PF_6^-]^{9+}$ (calcd *m/z*: 1299.2), 1154.7 $[M-10PF_6^-]^{10+}$ (calcd *m/z*: 1154.7), 1036.7 $[M-11PF_6^-]^{11+}$ (calcd *m/z*: 1036.7), 938.3 $[M-12PF_6^-]^{12+}$ (calcd *m/z*: 938.3), 854.9 $[M-13PF_6^-]^{13+}$ (calcd *m/z*: 854.9), 845.1 $[M-13PF_6^--PF_5]^{13+}$ (calcd *m/z*: 845.1), 783.5 $[M-14PF_6^-]^{14+}$ (calcd *m/z*: 783.5), 765.5 $[M-14PF_6^--2PF_5]^{14+}$ (calcd *m/z*: 765.5).

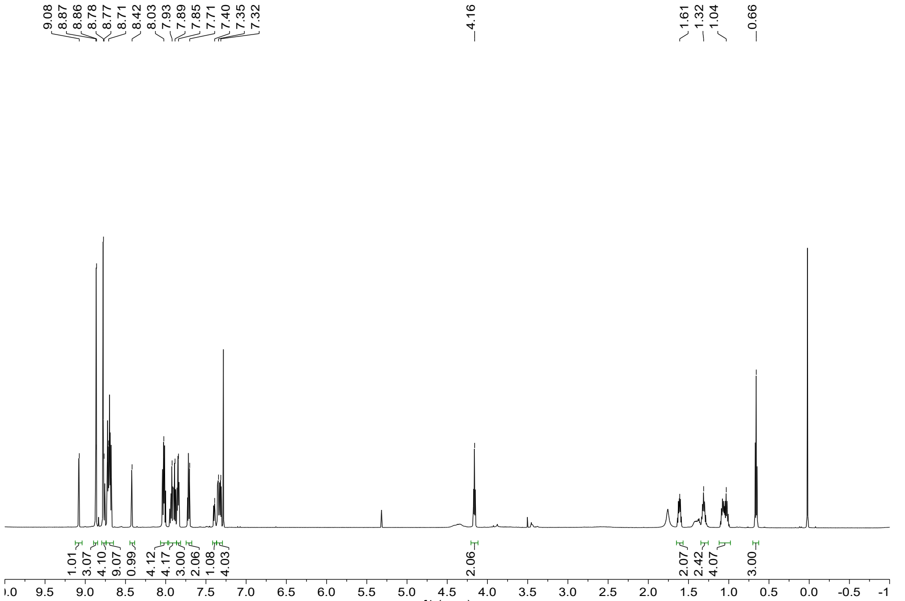

Supplementary Figure 40. ^{1}H NMR (600 MHz, $CDCl_3$, 300 K) spectrum of ligand **LA**.

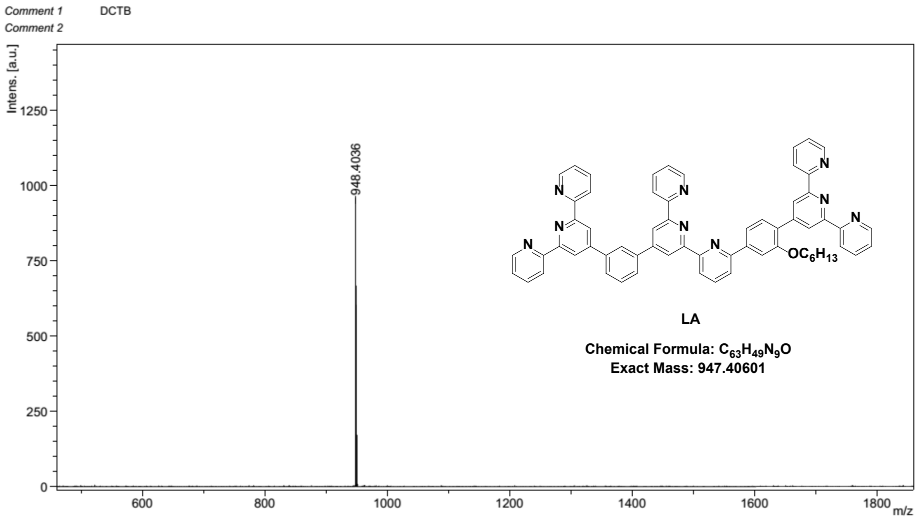

Supplementary Figure 41. MALDI-TOF plot of ligand **LA**.

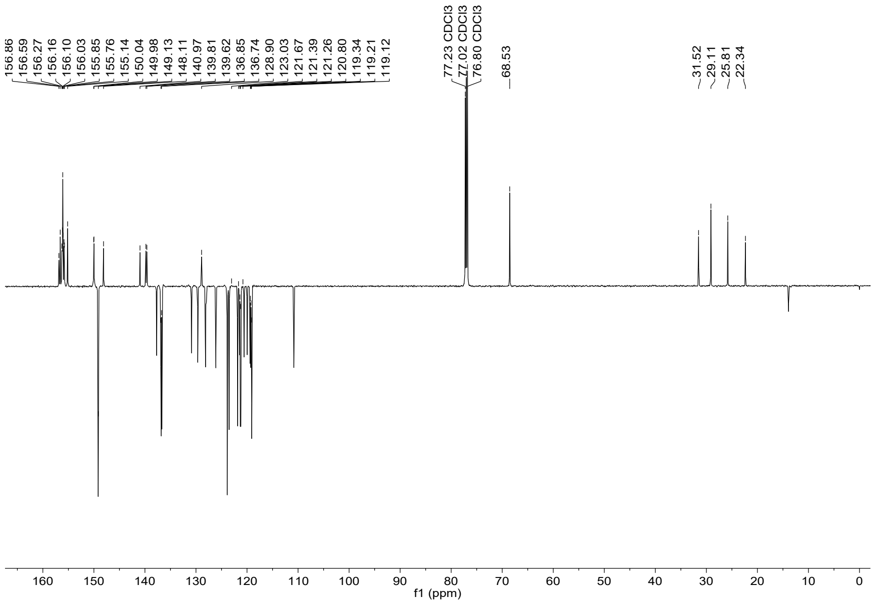

Supplementary Figure 42. DEPTQ ^{13}C NMR (150 MHz, $CDCl_3$, 300 K) spectrum of ligand **LA**.

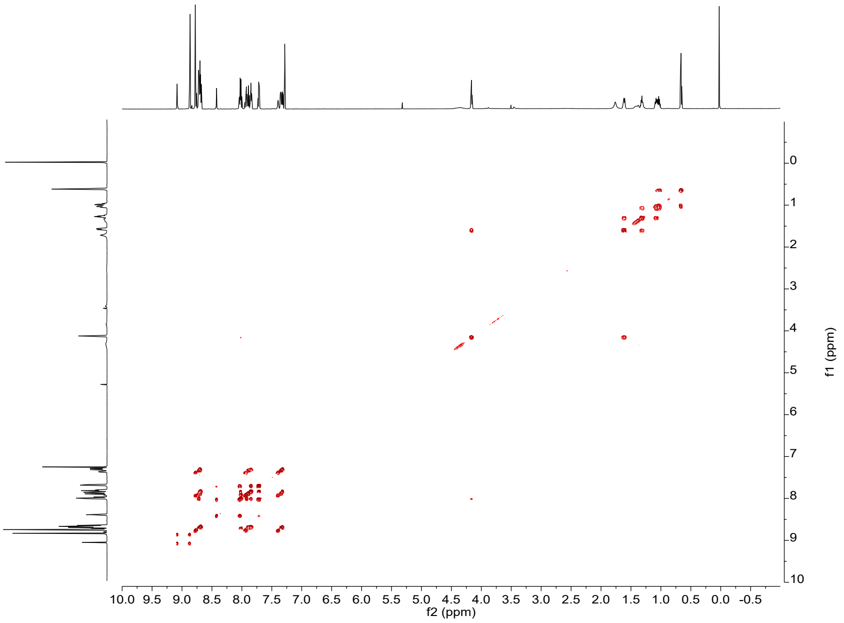

Supplementary Figure 43. 2D COSY NMR (600 MHz, $CDCl_3$, 300 K) spectrum of ligand **LA**.

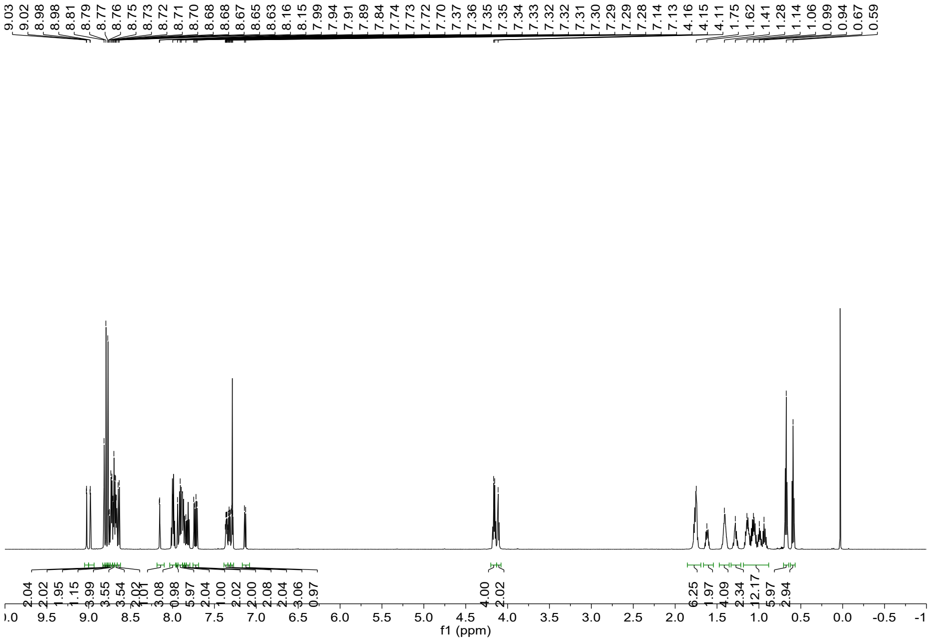

Supplementary Figure 44. ^{1}H NMR (600 MHz, $CDCl_3$, 300 K) spectrum of ligand **LB**.

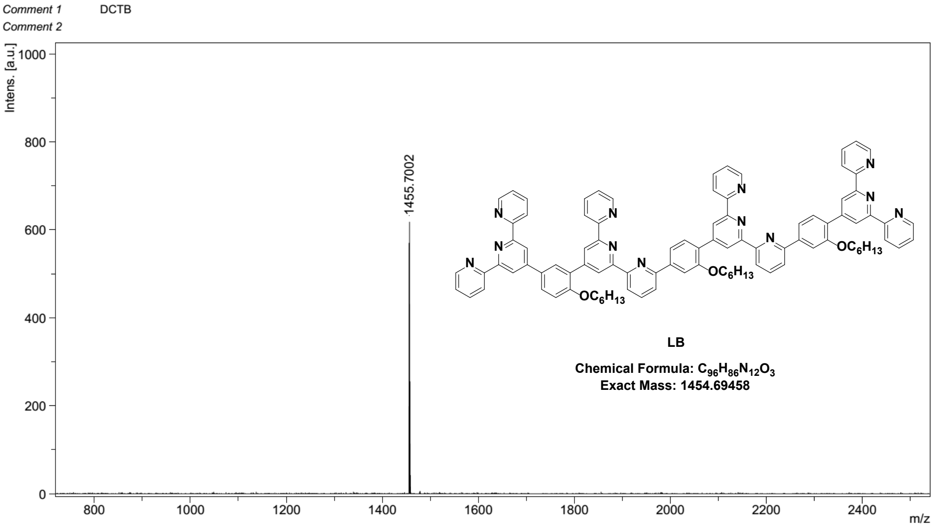

Supplementary Figure 45. MALDI-TOF plot of ligand **LB**.

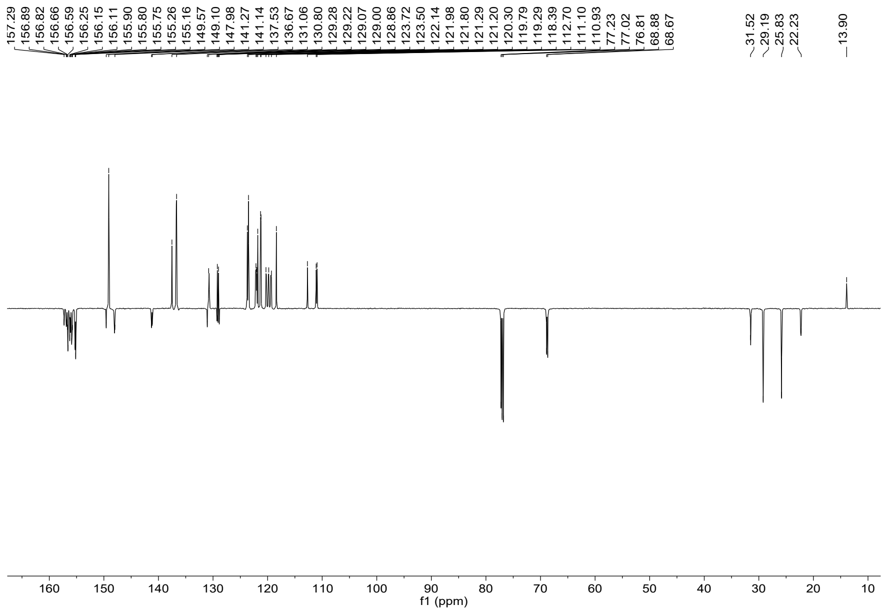

Supplementary Figure 46. DEPTQ ^{13}C NMR (150 MHz, $CDCl_3$, 300 K) spectrum of ligand **LB**.

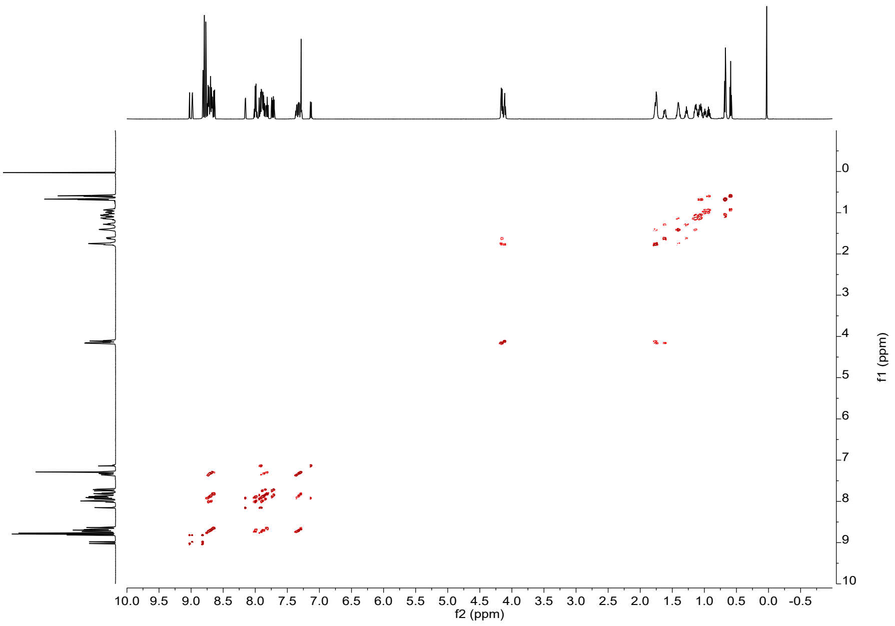

Supplementary Figure 47. 2D COSY NMR (600 MHz, $CDCl_3$, 300 K) spectrum of ligand **LB**.

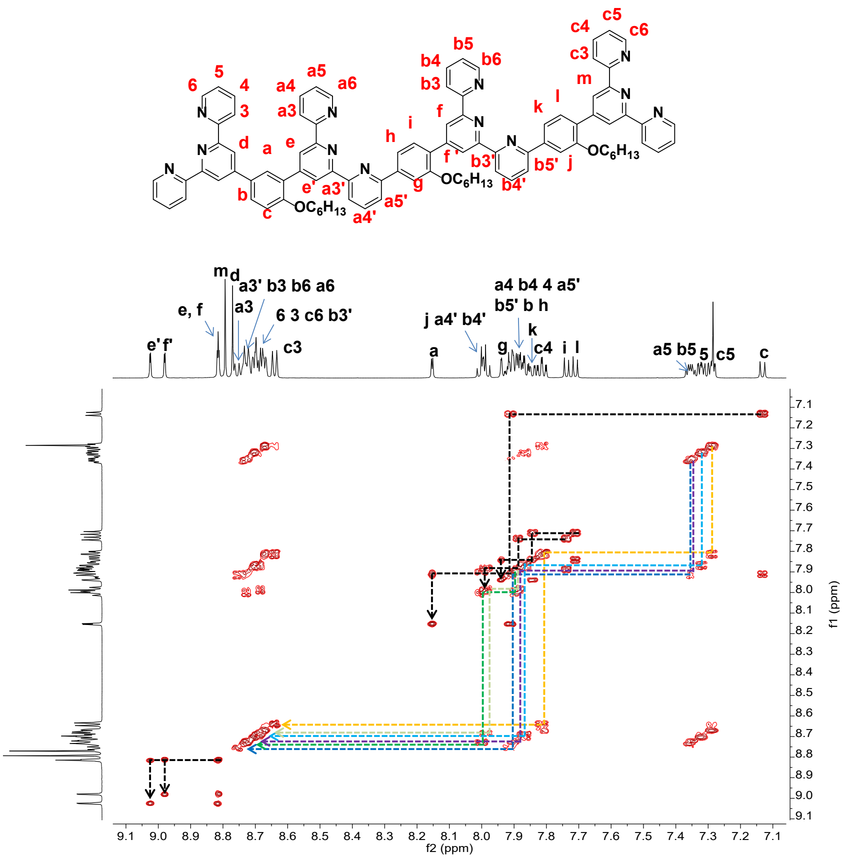

Supplementary Figure 48. 2D COSY NMR (600 MHz, $CDCl_3$, 300 K) spectrum of ligand **LB** (aromatic region).

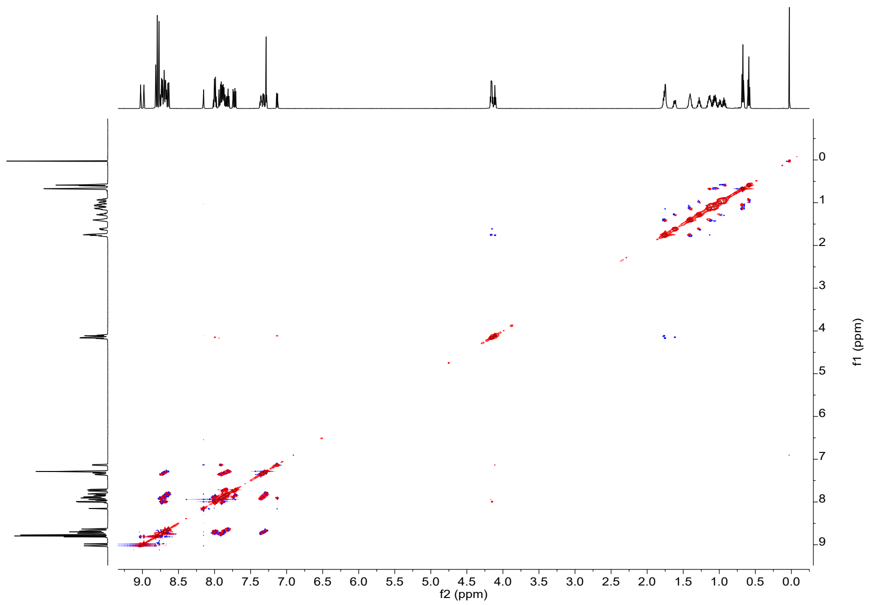

Supplementary Figure 49. 2D NOESY NMR (600 MHz, $CDCl_3$, 300 K) spectrum of ligand **LB**.

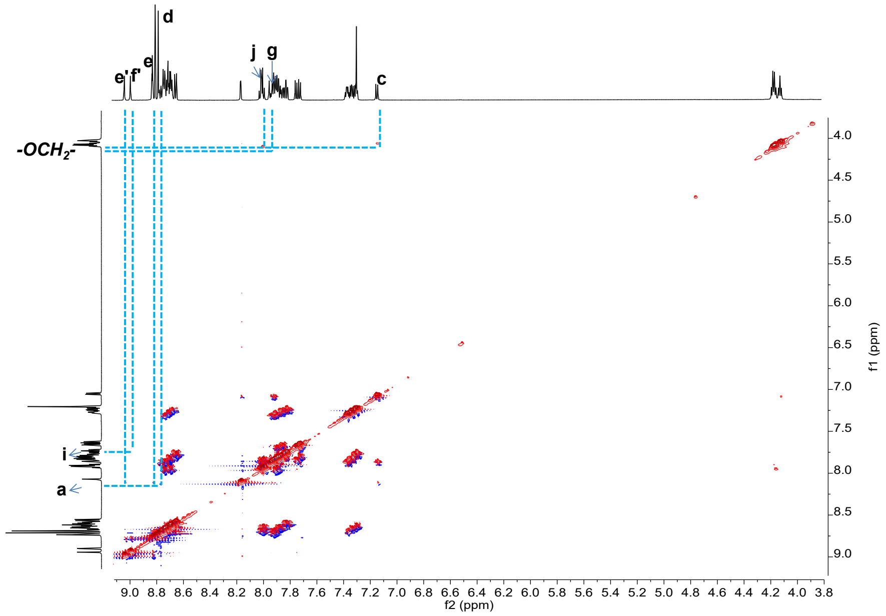

Supplementary Figure 50. 2D NOESY NMR (600 MHz, $CDCl_3$, 300 K) spectrum of ligand **LB** (aromatic region).

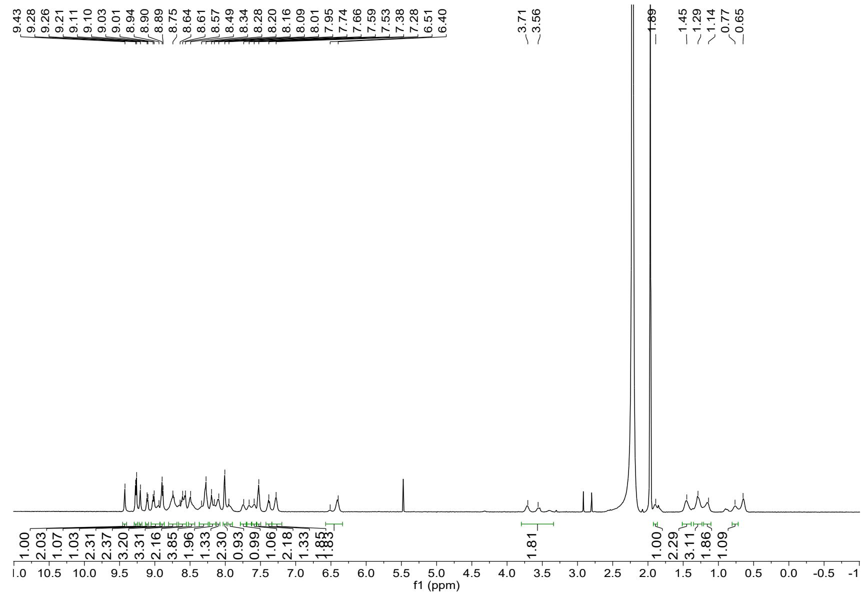

Supplementary Figure 51. ^{1}H NMR (600 MHz, CD_3CN, 300 K) spectrum of complex **$Zn_9(LA)_6$**.

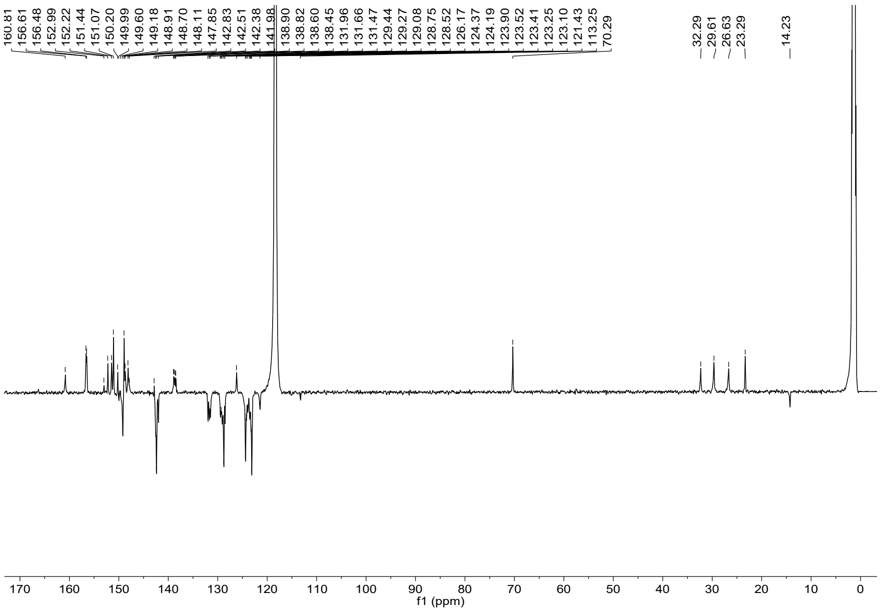

Supplementary Figure 52. DEPTQ ^{13}C NMR (150 MHz, CD_3CN, 300 K) spectrum of complex **$Zn_9(LA)_6$**.

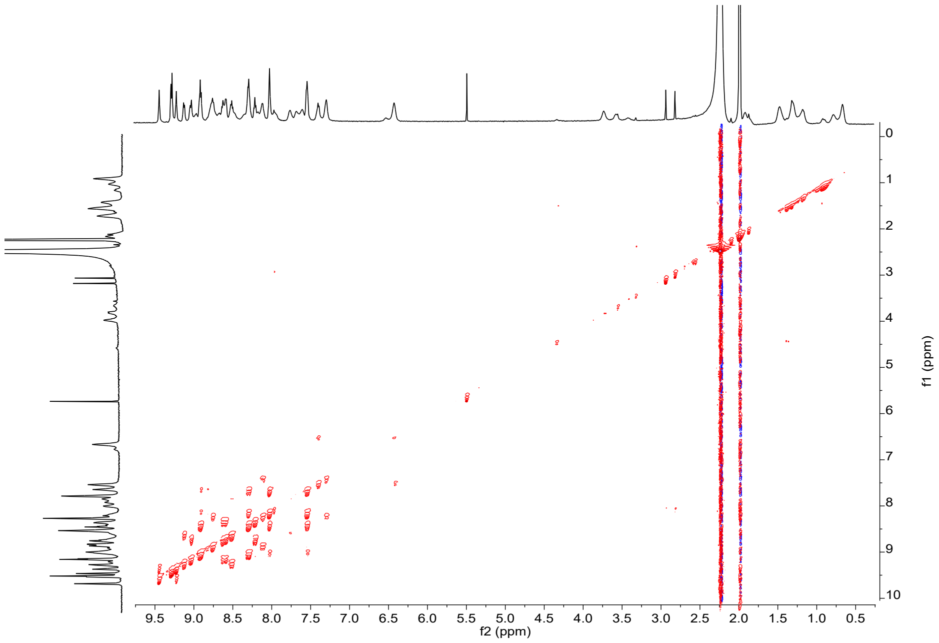

Supplementary Figure 53. 2D COSY NMR (600 MHz, CD_3CN, 300 K) spectrum of complex **$Zn_9(LA)_6$**.

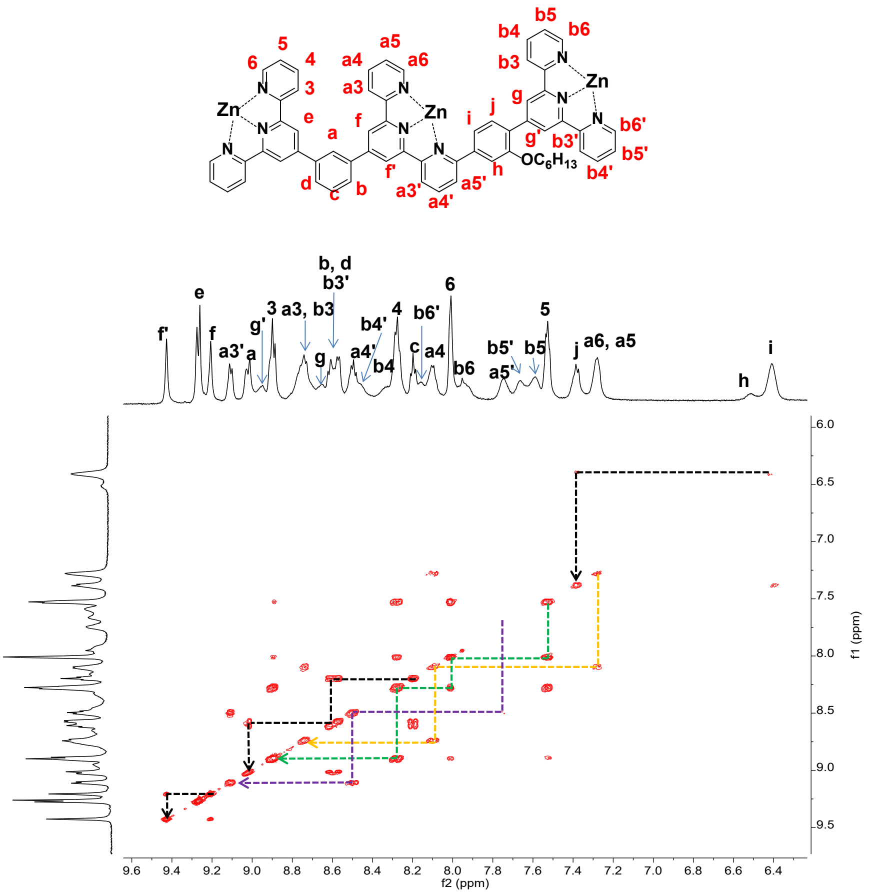

Supplementary Figure 54. 2D COSY NMR (600 MHz, CD_3CN, 300 K) spectrum of complex $\mathbf{Zn_9(LA)_6}$ (aromatic region).

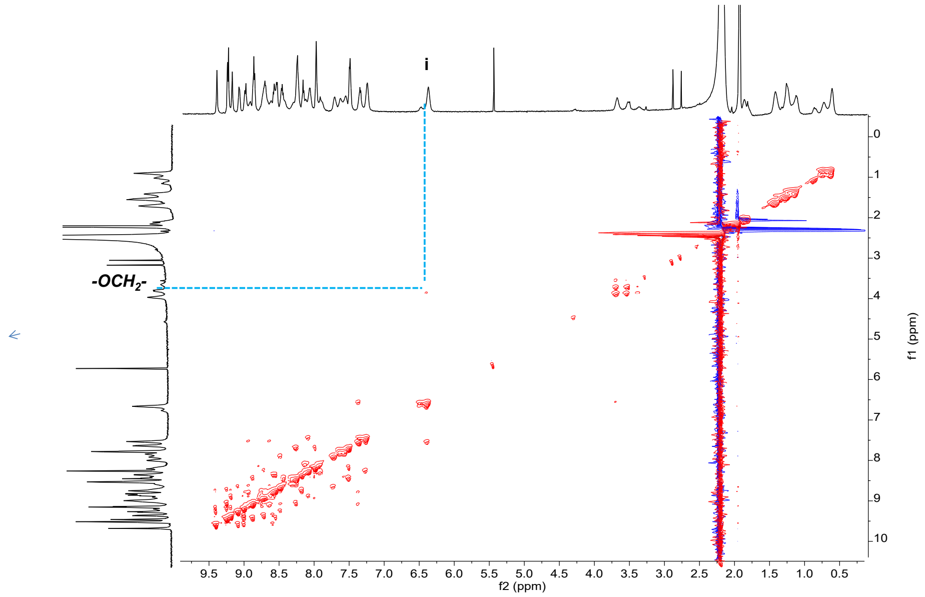

Supplementary Figure 55. 2D NOESY NMR (600 MHz, CD_3CN, 300 K) spectrum of ligand **$Zn_9(LA)_6$**.

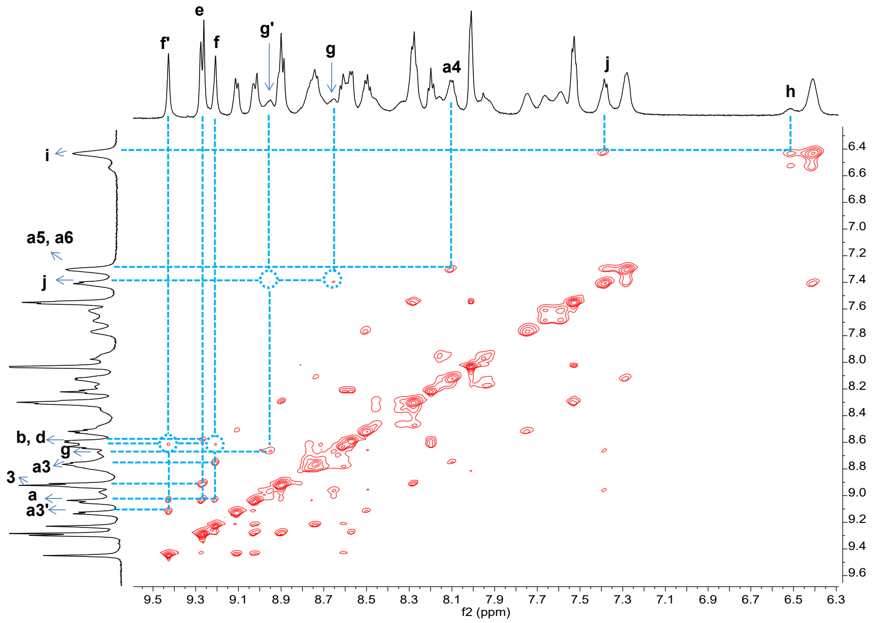

Supplementary Figure 56. 2D NOESY NMR (600 MHz, CD_3CN, 300 K) spectrum of ligand **$Zn_9(LA)_6$** (aromatic region).

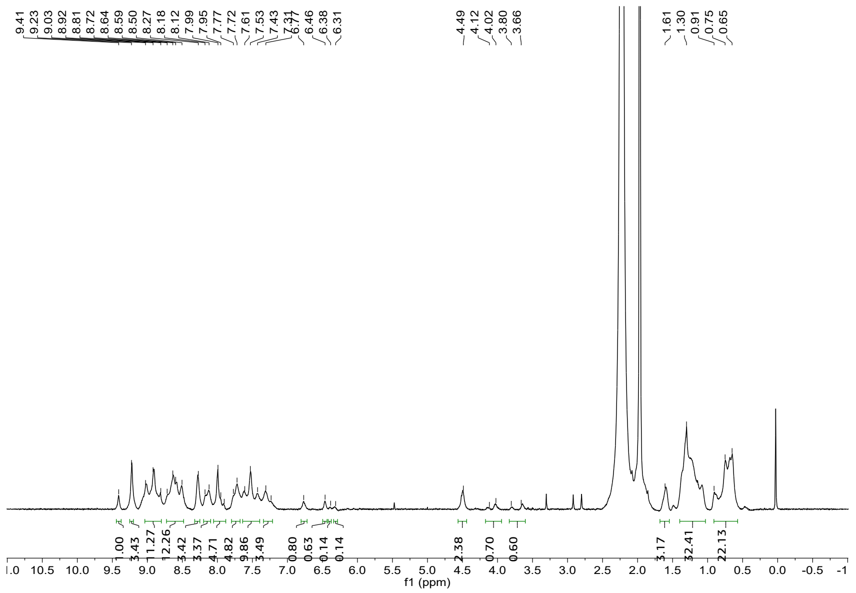

Supplementary Figure 57. ^{1}H NMR (600 MHz, CD_3CN, 300 K) spectrum of complex **$Zn_{12}(LB)_6$**.

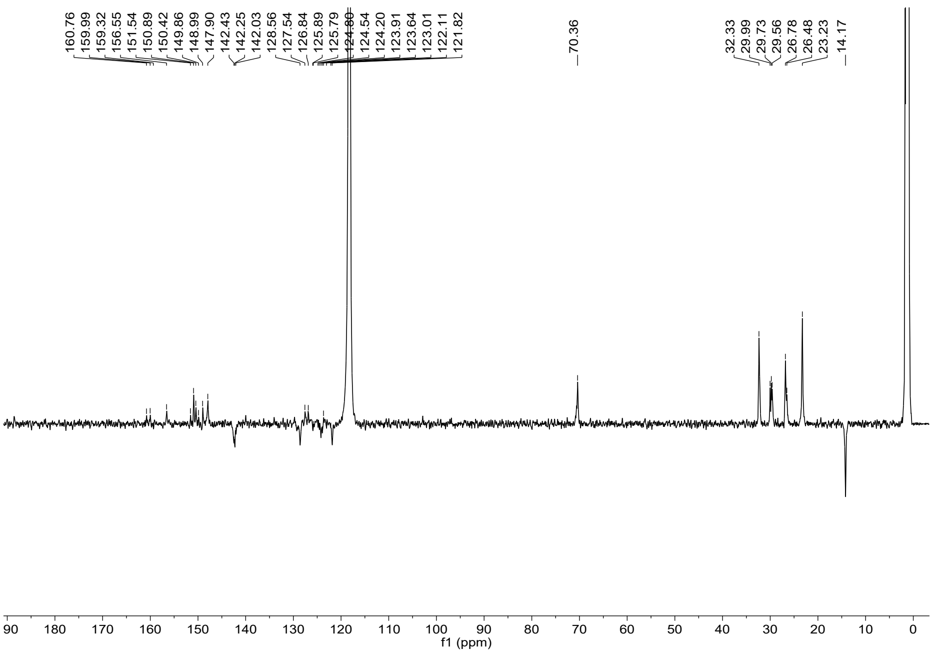

Supplementary Figure 58. DEPTQ ^{13}C NMR (150 MHz, CD_3CN, 300 K) spectrum of complex **$Zn_{12}(LB)_6$**.

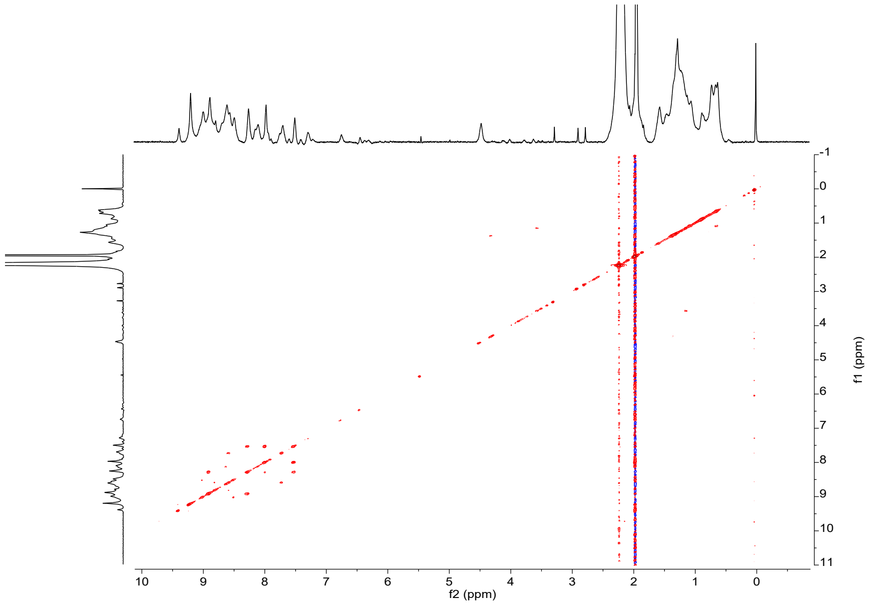

Supplementary Figure 59. 2D COSY NMR (600 MHz, CD_3CN, 300 K) spectrum of complex $\mathbf{Zn_{12}(LB)_6}$.

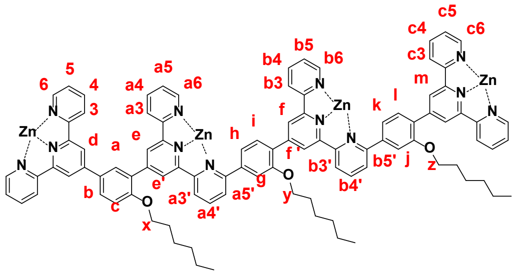

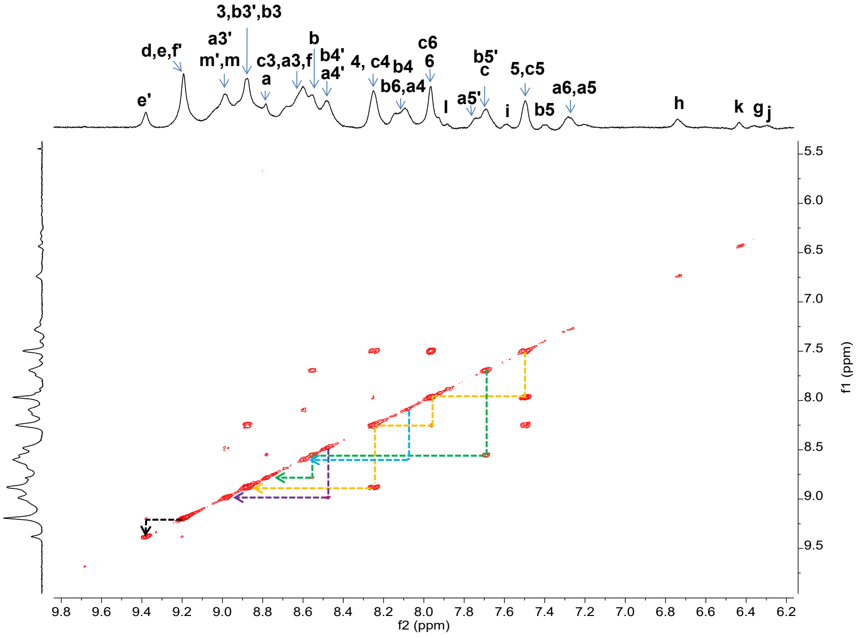

Supplementary Figure 60. 2D COSY NMR (600 MHz, CD_3CN, 300 K) spectrum of ligand $\mathbf{Zn_{12}(LB)_6}$ (aromatic region).

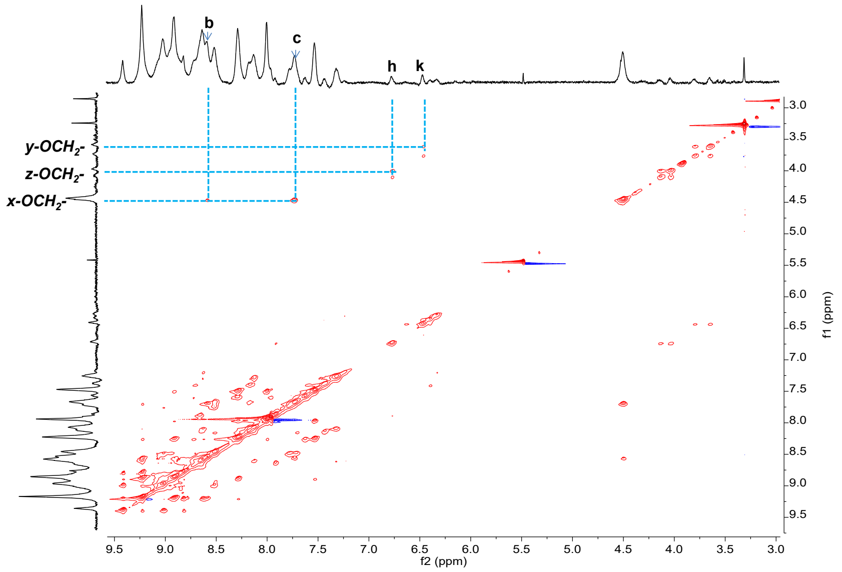

Supplementary Figure 61. 2D NOESY NMR (600 MHz, CD_3CN, 300 K) spectrum of complex $\mathbf{Zn_{12}(LB)_6}$.

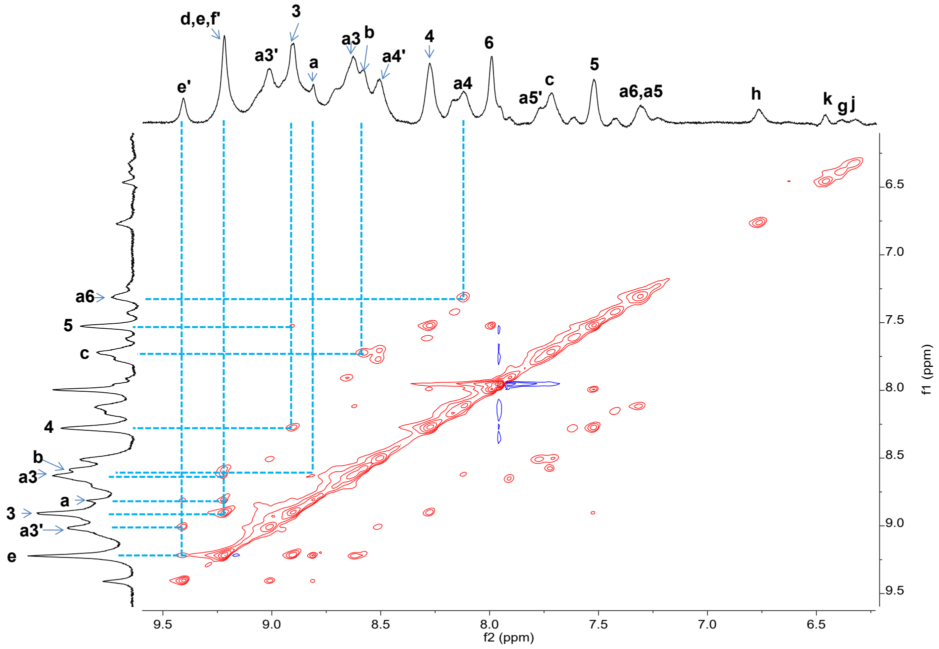

Supplementary Figure 62. 2D NOESY NMR (600 MHz, CD_3CN, 300 K) spectrum of ligand $\mathbf{Zn_{12}(LB)_6}$ (aromatic region).

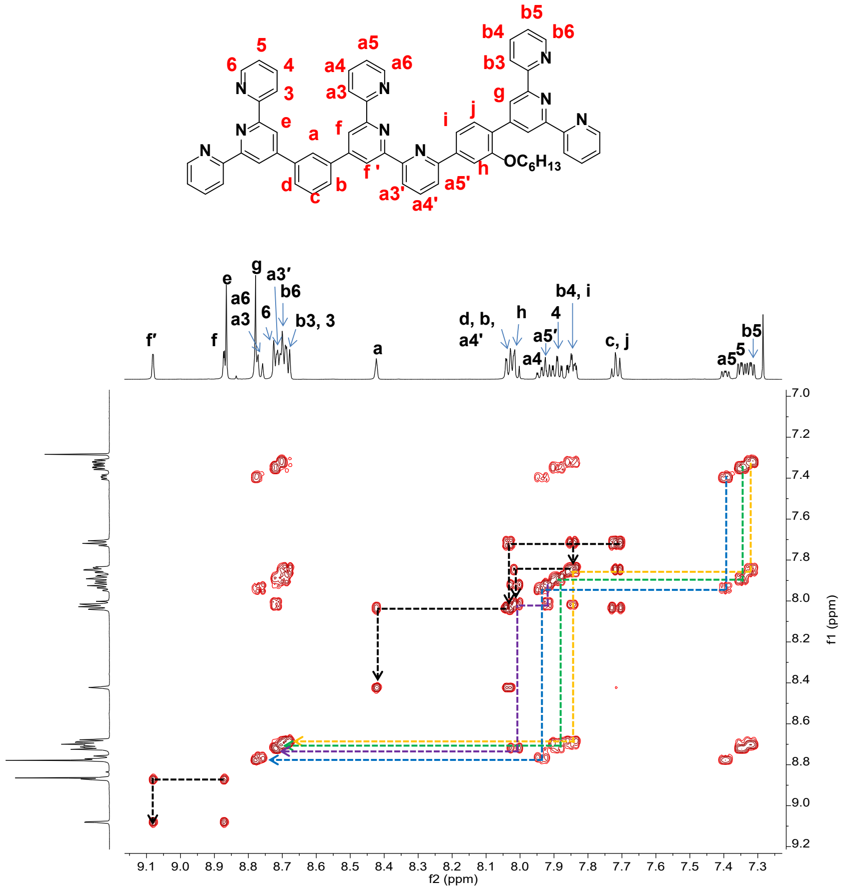

Supplementary Figure 63. 2D COSY NMR (600 MHz, $CDCl_3$, 300 K) spectrum of ligand **LA** (aromatic region).

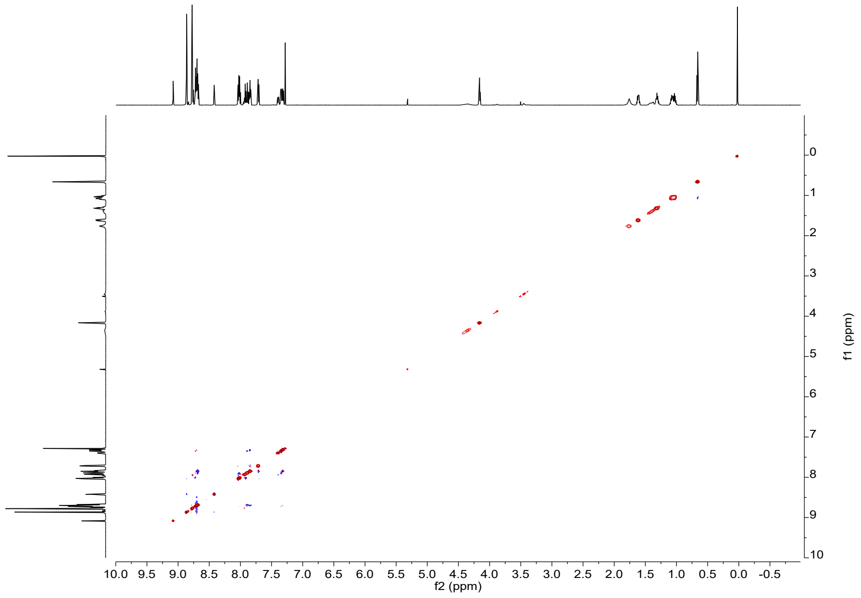

Supplementary Figure 64. 2D NOESY NMR (600 MHz, $CDCl_3$, 300 K) spectrum of ligand **LA**.

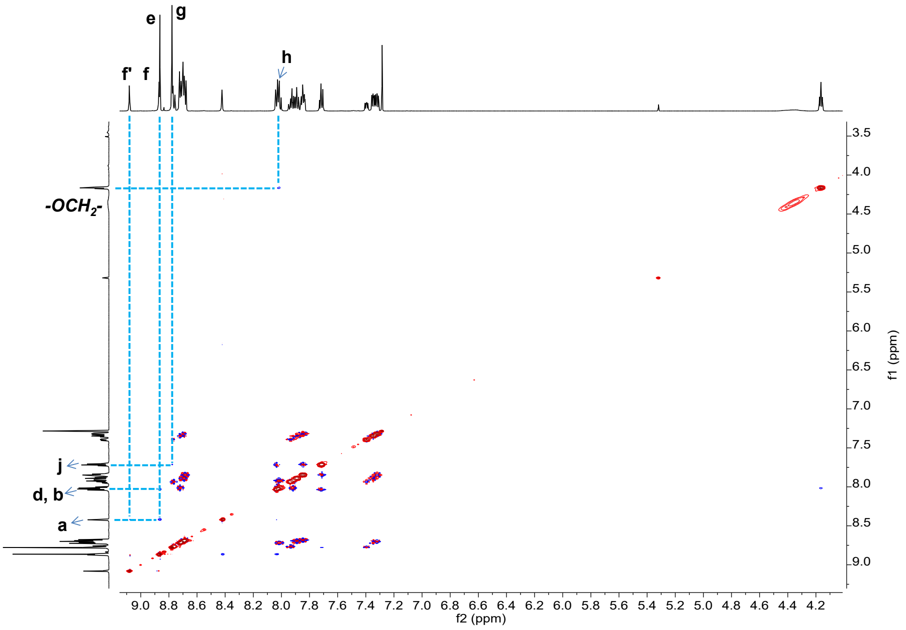

Supplementary Figure 65. 2D NOESY NMR (600 MHz, $CDCl_3$, 300 K) spectrum of ligand **LA**.

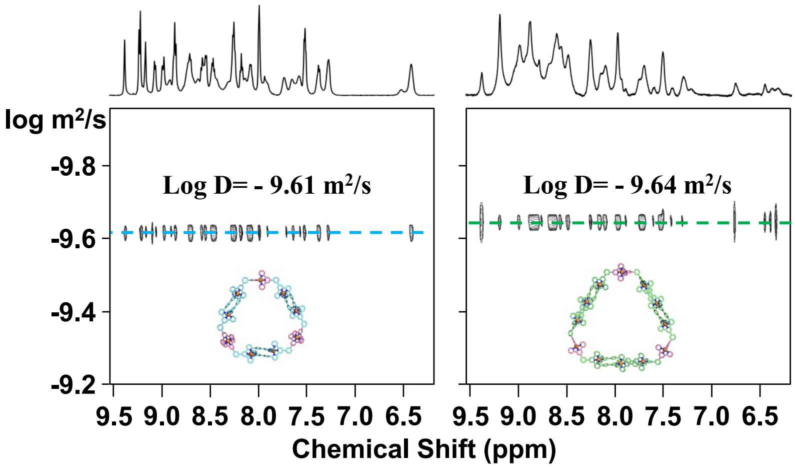

Supplementary Figure 66. 2D DOSY NMR spectra of hexagons $\mathbf{Zn_9(LA)_6}$ (left) and $\mathbf{Zn_{12}(LB)_6}$ (right).

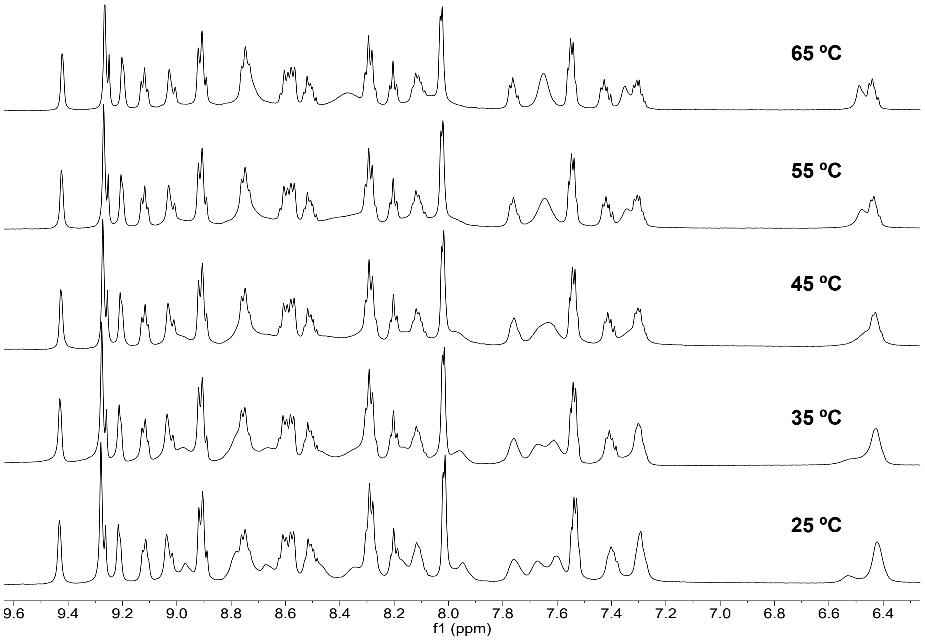

Supplementary Figure 67. Variable-temperature (25 °C - 65 °C) ^{1}H NMR (500 MHz) signals of the aromatic protons for complex **$Zn_9(LA)_6$** in CD_3CN (4 mg/mL).

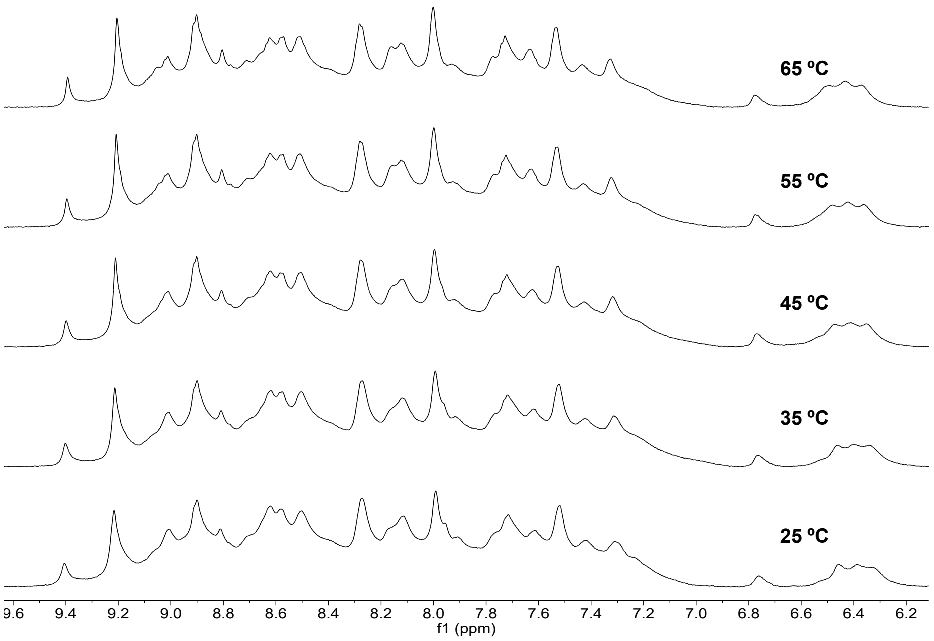

Supplementary Figure 68. Variable-temperature (25 °C - 65 °C) ^{1}H NMR (500 MHz) signals of the aromatic protons for complex **$Zn_{12}(LB)_6$** in CD_3CN (4 mg/mL).

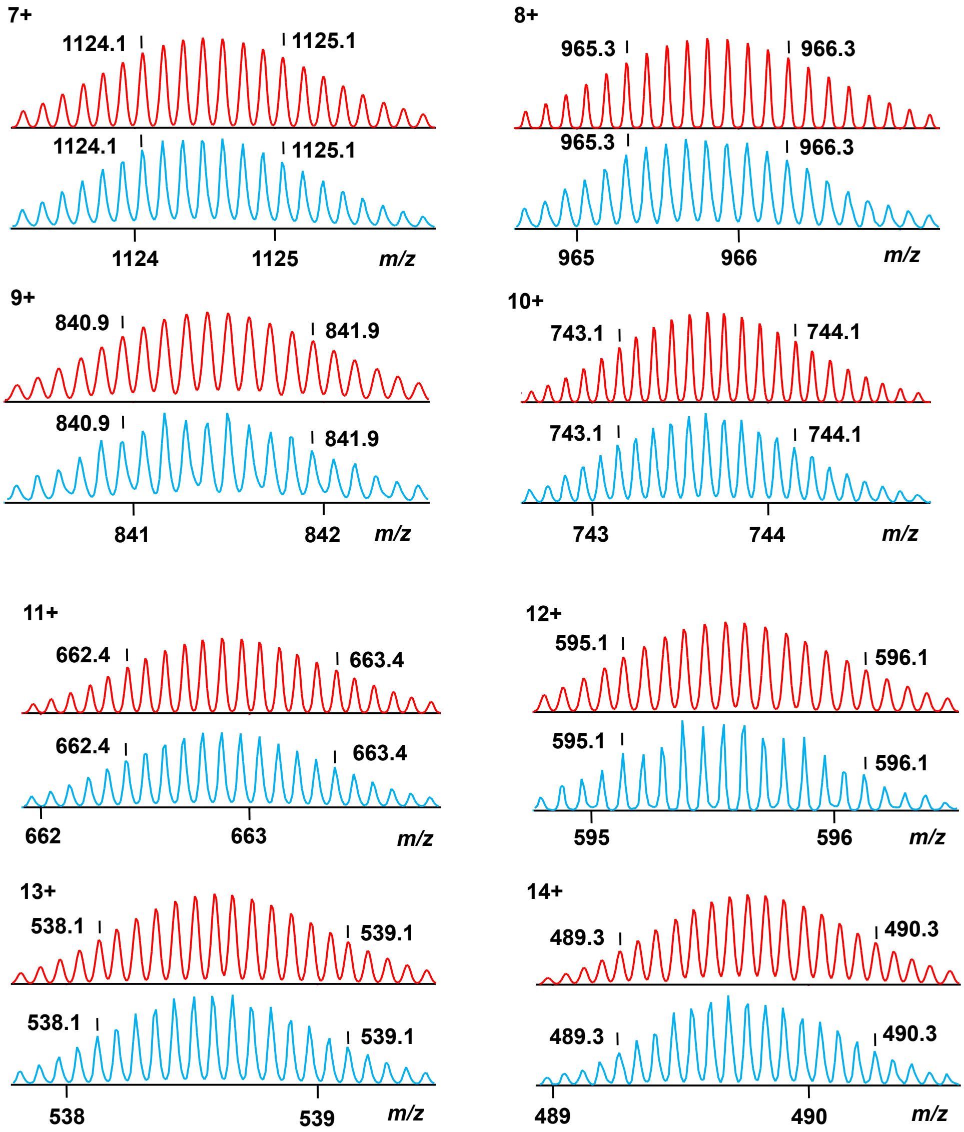

Supplementary Figure 69. Measured (blue) and calculated (red) isotope patterns for different charge states observed from **$Zn_9(LA)_6$** (PF_6^- as counterion).

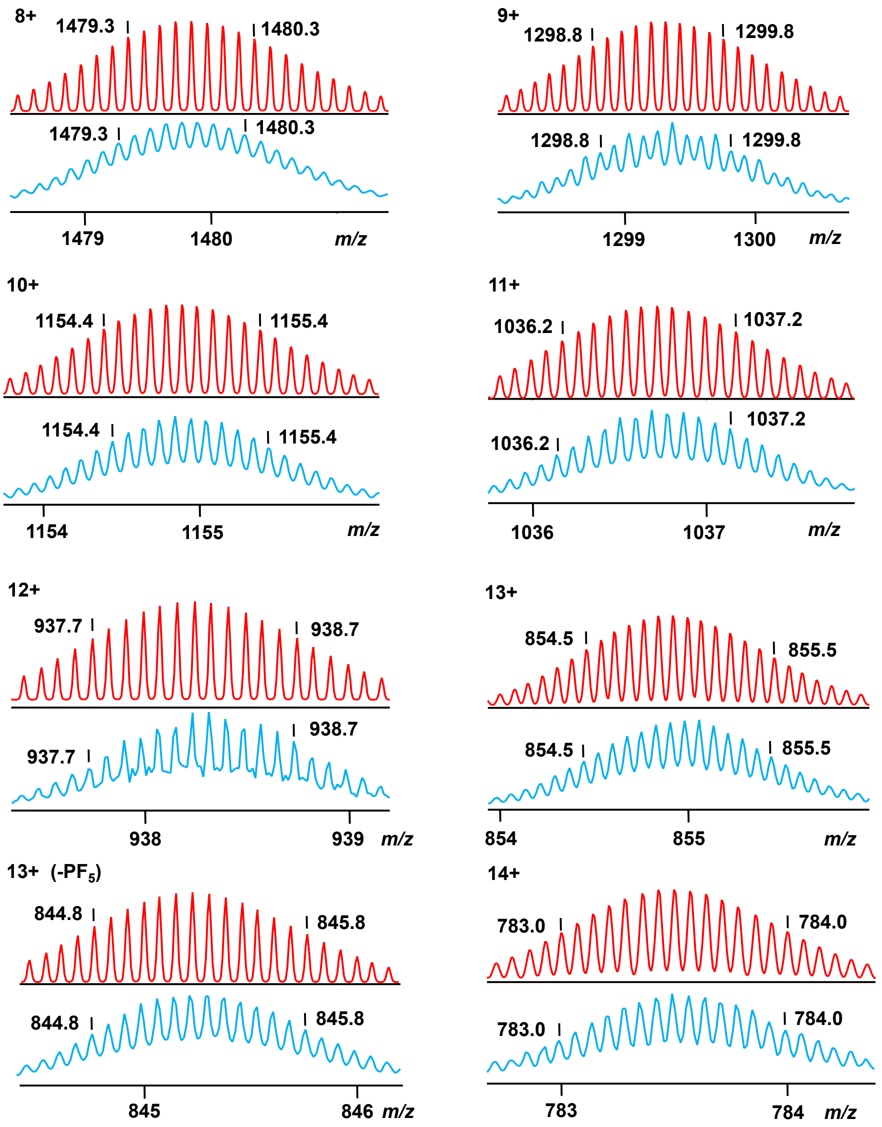

Supplementary Figure 70. Measured (blue) and calculated (red) isotope patterns for different charge states observed from $\mathbf{Zn_{12}(LB)_6}$ (PF_6^- as counterion).

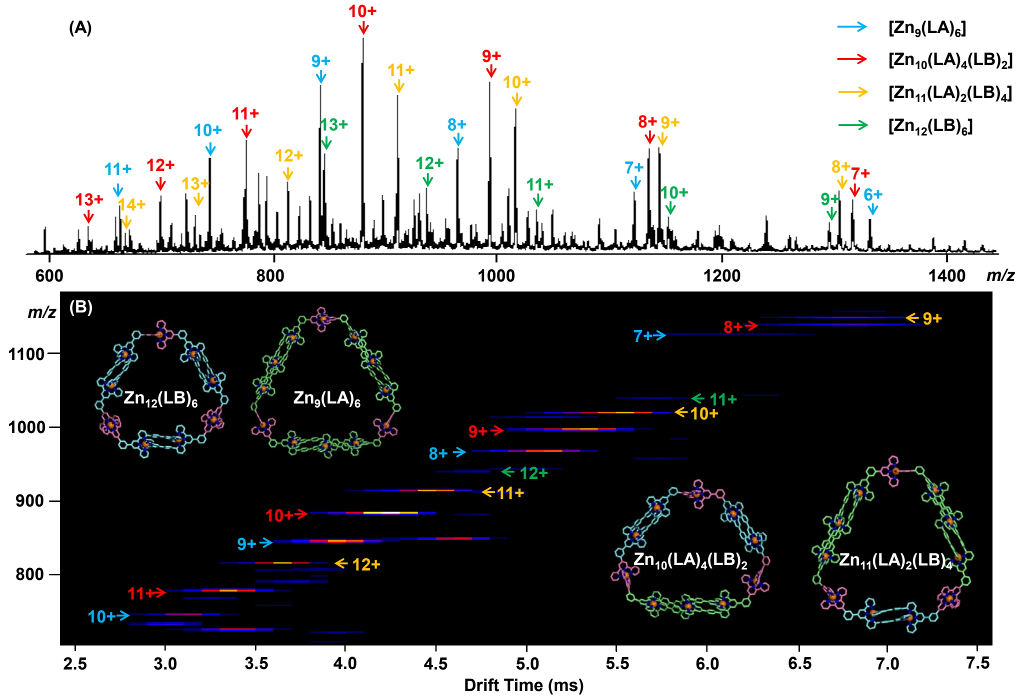

Supplementary Figure 71. (A) ESI-MS and (B) TWIM-MS plots of the binary mixture of **$Zn_9(LA)_6$** and **$Zn_{12}(LB)_6$**. Inset were the molecular modeling of **$Zn_9(LA)_6$**, **$Zn_{12}(LB)_6$**, **$Zn_{10}(LA)_4(LB)_2$** and **$Zn_{11}(LA)_2(LB)_4$**; alkyl chains were omitted for clarity.

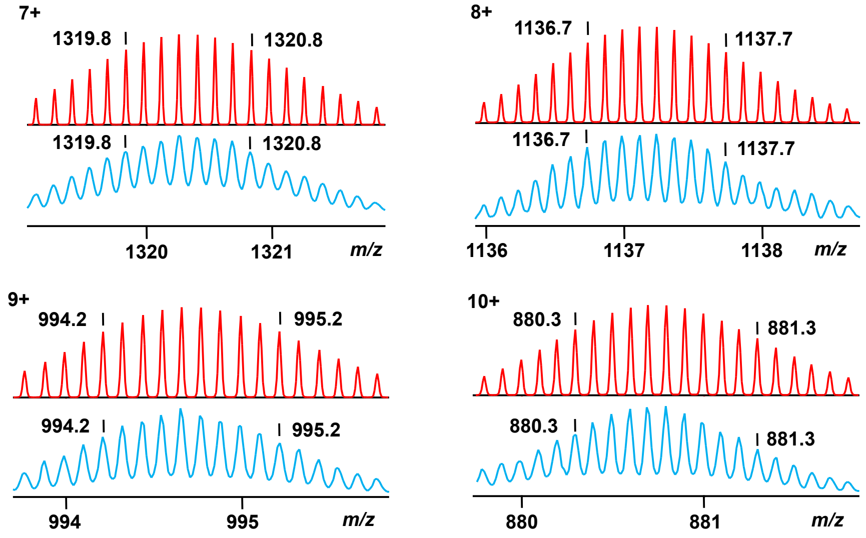

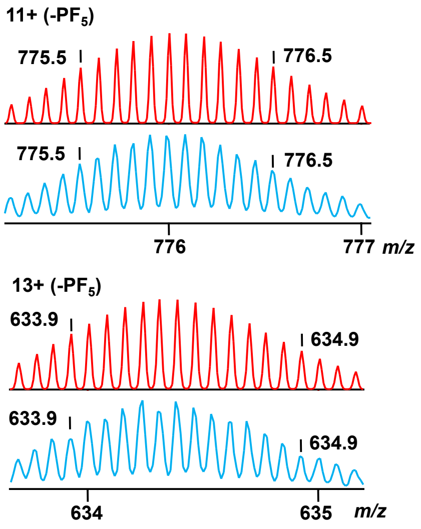

Supplementary Figure 72. Measured (blue) and calculated (red) isotope patterns for different charge states observed from $\mathbf{Zn_{10}(LA)_4(LB)_2}$ (PF_6^- as counterion).

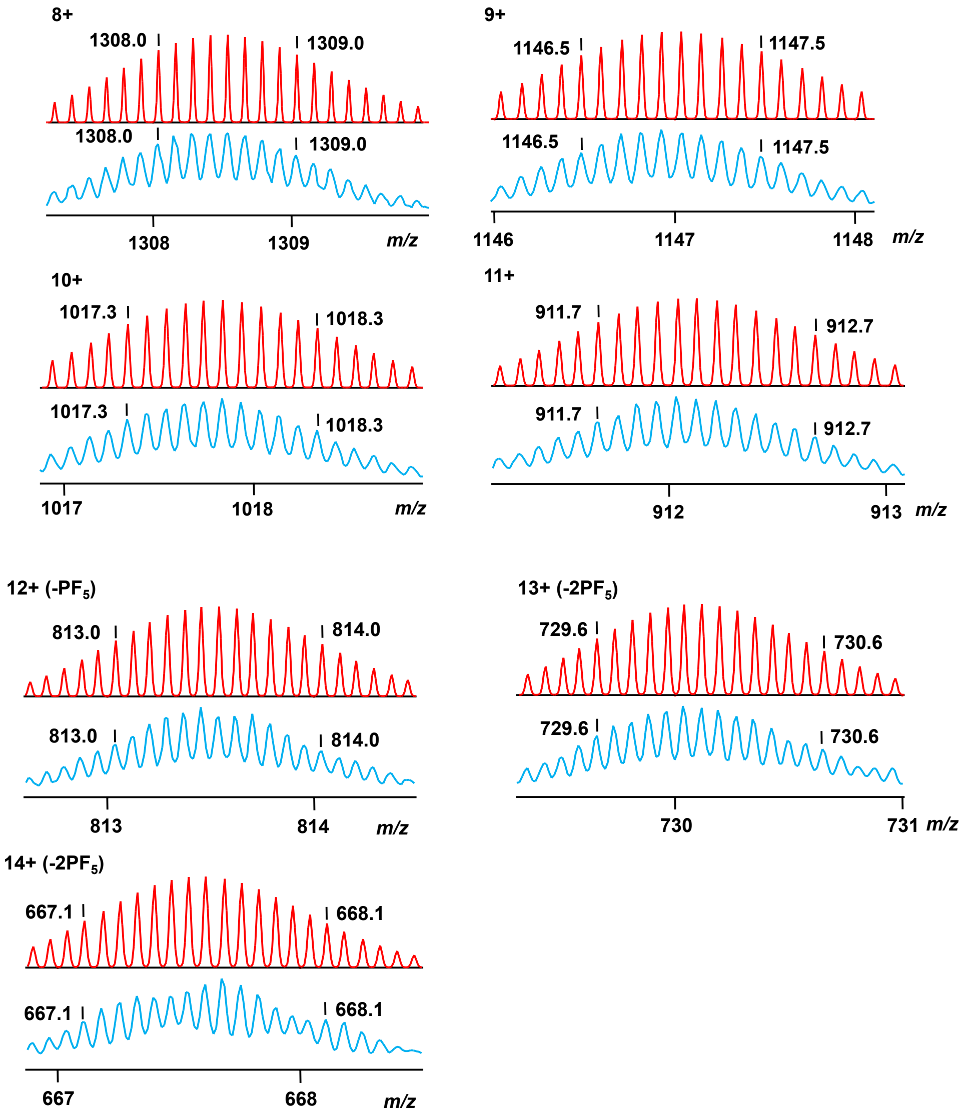

Supplementary Figure 73. Measured (blue) and calculated (red) isotope patterns for different charge states observed from **$Zn_{11}(LA)_2(LB)_4$** (PF_6^- as counterion).

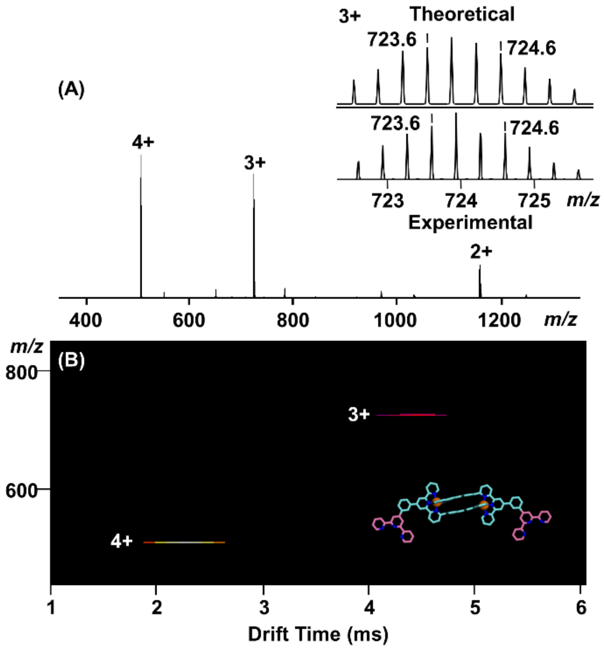

Supplementary Figure 74. (A) ESI-MS and (B) TWIM-MS plots (*m/z* vs drift time) of **$Zn_2(LA)_2$**.

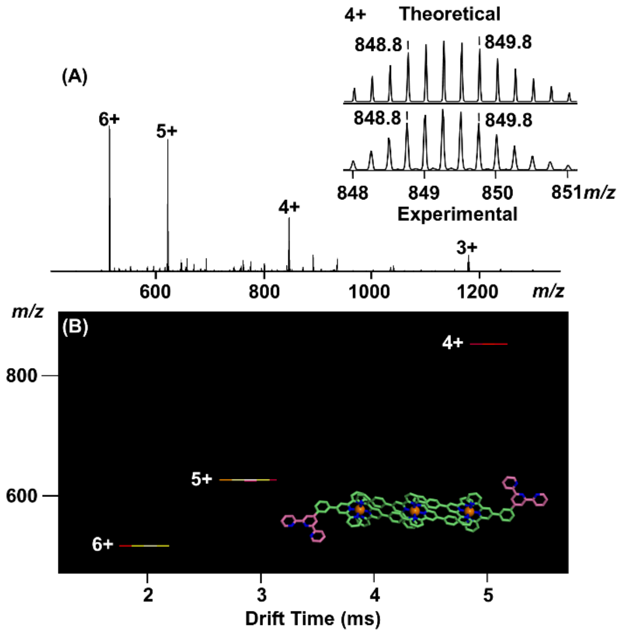

Supplementary Figure 75. (A) ESI-MS and (B) TWIM-MS plots (*m/z* vs drift time) of **$Zn_3(LB)_2$**.

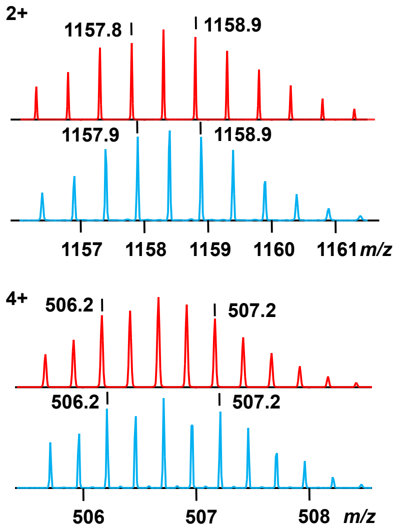

Supplementary Figure 76. Measured (blue) and calculated (red) isotope patterns for different charge states observed from **$Zn_2(LA)_2$** (PF_6^- as counterion).

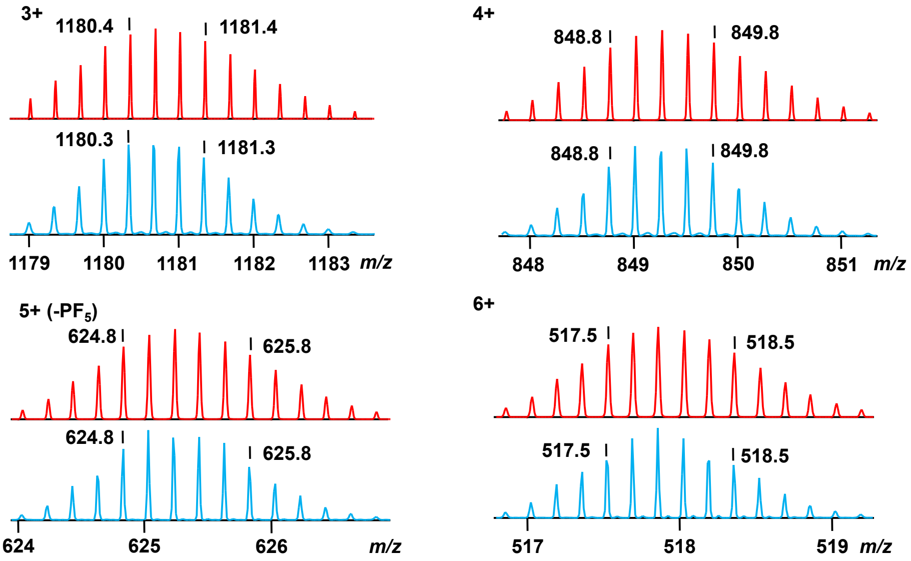

Supplementary Figure 77. Measured (blue) and calculated (red) isotope patterns for different charge states observed from **$Zn_3(LB)_2$** (PF_6^- as counterion).

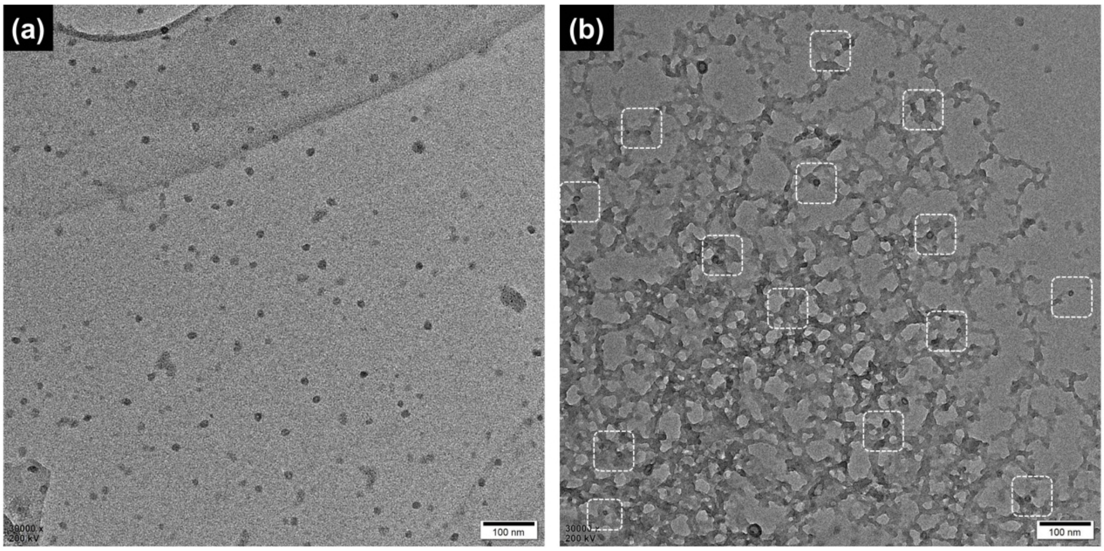

Table S1 Statistical report

Distr./nm	Mean/nm	Amount	Freq.
16-16.9	16.45	3	7.50%
16.9-17.8	17.35	1	2.50%
17.8-18.7	18.25	3	7.50%
18.7-19.6	19.15	7	17.50%
19.6-20.5	20.05	8	20.00%
20.5-21.4	20.95	6	15.00%
21.4-22.3	21.85	2	5.00%
22.3-23.2	22.75	4	10.00%
23.2-24.1	23.65	4	10.00%
24.1-25	24.55	2	5.00%

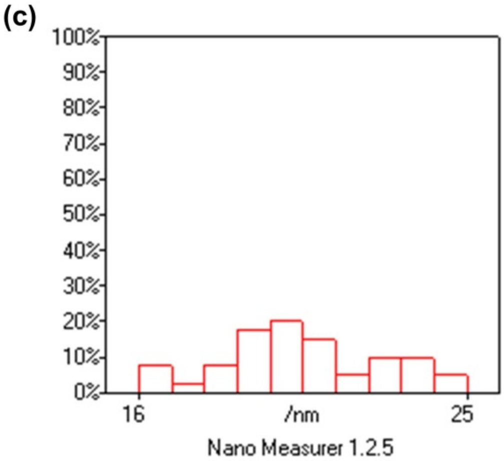

Supplementary Figure 78. TEM images of (a) assembled cyclic nanostructures of **$Zn_9(LA)_6$** at concentration of 10^{-5} M, (b) 3D network structure formed by **$Zn_9(LA)_6$** at concentration of 10^{-4} M and (c) histogram distribution of the diameters of the assembled cyclic nanostructures of **$Zn_9(LA)_6$**.

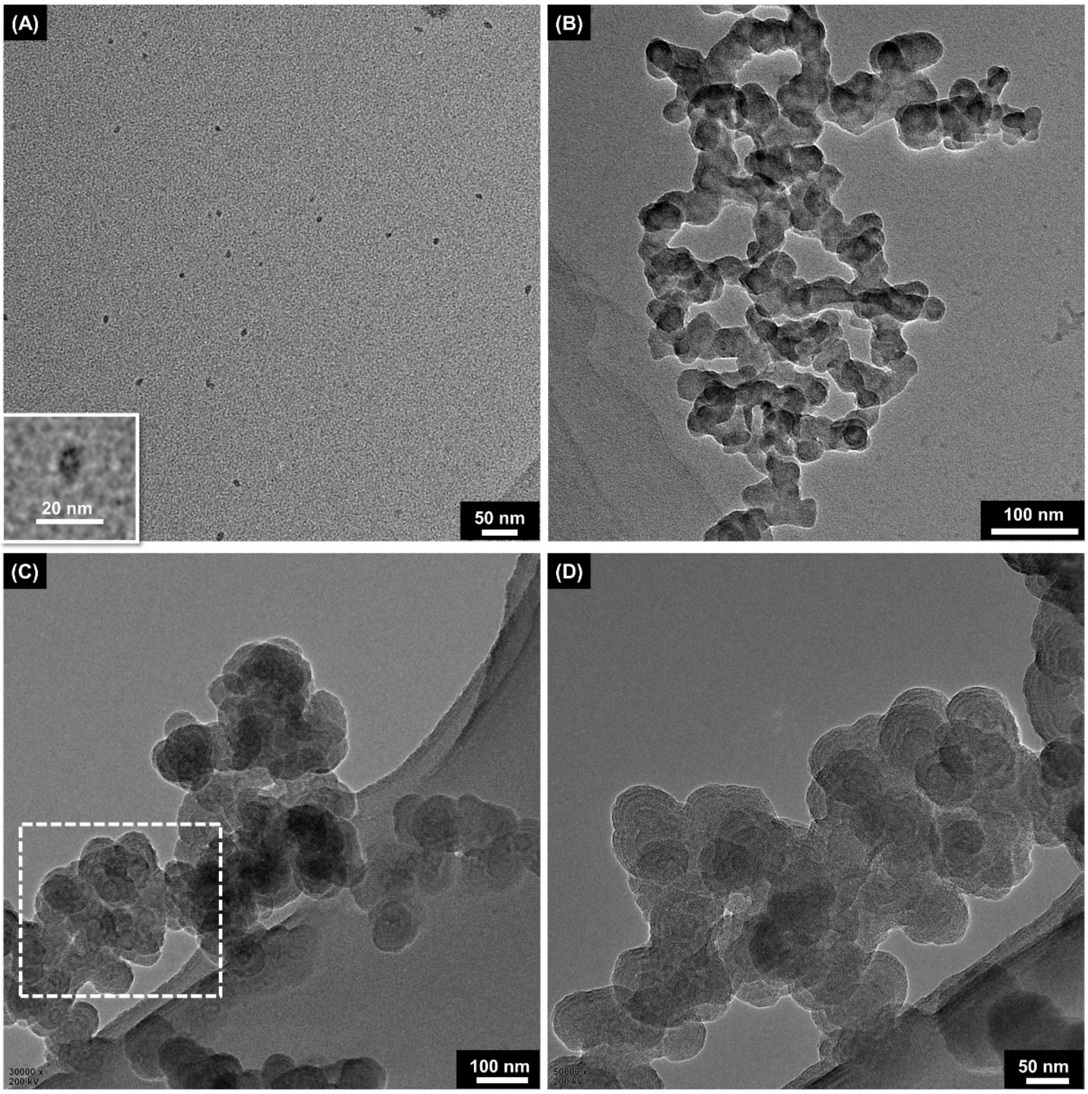

Supplementary Figure 79. TEM images of (A) individual $\mathbf{Zn_{12}(LB)_6}$ at concentration of 10^{-6} M, (B) assembled cyclic nanostructures of $\mathbf{Zn_{12}(LB)_6}$ at concentration of 10^{-5} M, (C) 3D network structure formed by $\mathbf{Zn_{12}(LB)_6}$ at concentration of 10^{-4} M, and (D) zoomed-in image of the white rectangle at image C. The solvent was CH_3CN.

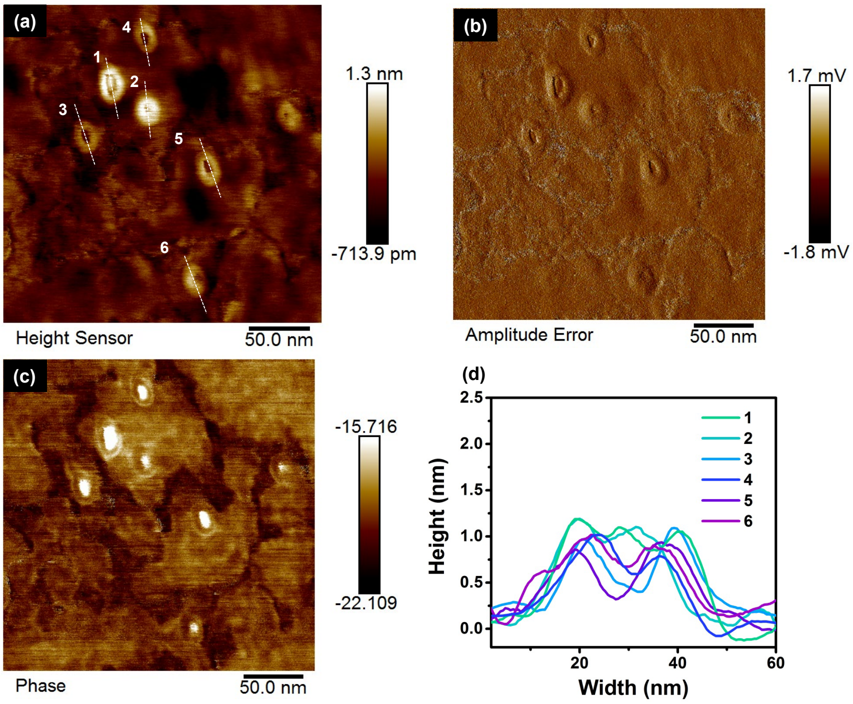

Supplementary Figure 80. The origin data of AFM images of cyclic nanostructures formed by $\mathbf{Zn_9(LA)_6}$, (a) height, (b) amplitude error, (c) phase images of air-dried CH_3CN (10^{-5} M) dispersion on a silicon wafer substrate and (d) the cross-section of the cyclic nanostructure shown in image a.

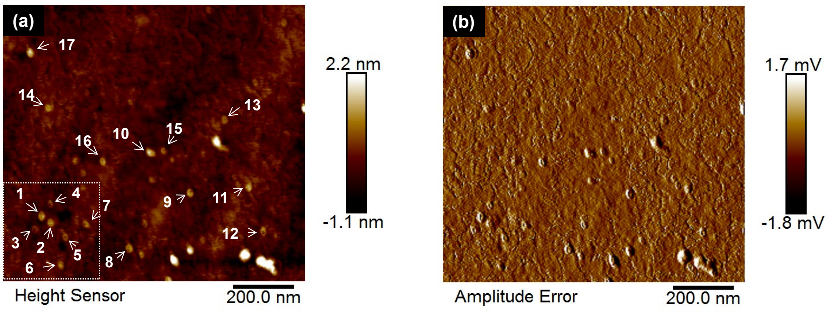

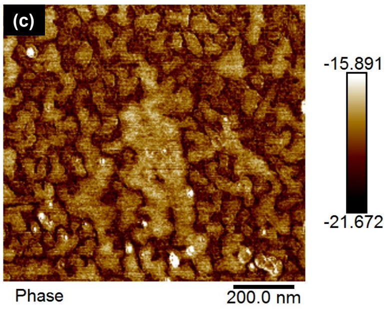

Entry	Diameter (nm)	Height (nm)	Entry	Diameter (nm)	Height (nm)
1	35	1.3	10	35	1.5
2	33	1.3	11	32	1.1
3	33	1.2	12	36	1.1
4	33	0.9	13	33	0.8
5	34	0.8	14	33	1.3
6	36	0.9	15	29	0.8
7	32	0.8	16	34	1.3
8	32	1.2	17	34	1.4
9	32	1.4	AVGE	33	1.2

Supplementary Figure 81. The origin data of AFM images of cyclic nanostructures formed by $\mathbf{Zn_9(LA)_6}$ before zooming, (a) height, (b) amplitude error, (c) phase images of air-dried CH_3CN (10^{-5} M) dispersion on a silicon wafer substrate and (d) the cross-section of the cyclic nanostructure shown in image a.

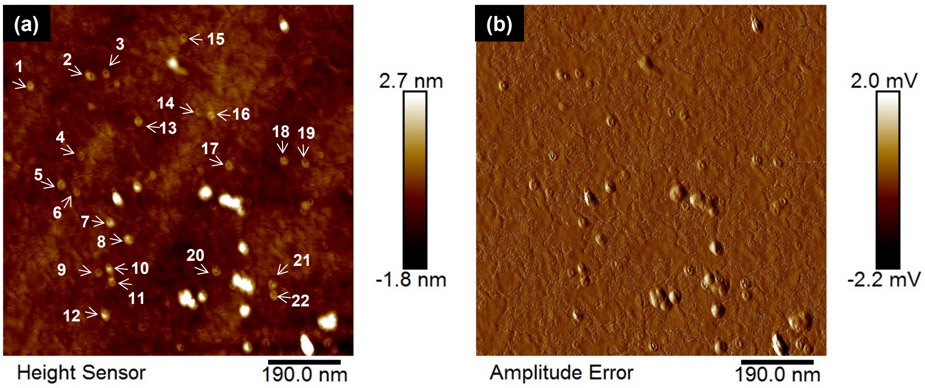

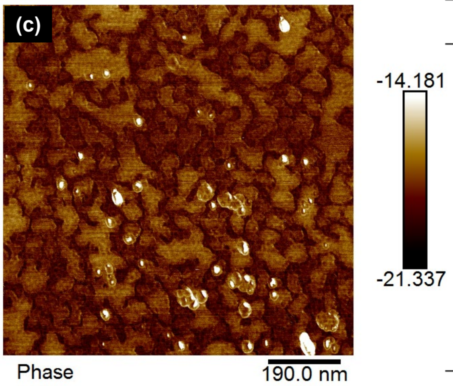

Entry	Diameter (nm)	Height (nm)	Entry	Diameter (nm)	Height (nm)
1	31	1.3	13	30	1.2
2	35	1.3	14	29	0.8
3	30	1.0	15	28	1.0
4	33	0.8	16	30	0.9
5	34	1.1	17	27	1.0
6	29	1.0	18	28	1.2
7	29	1.4	19	30	1.0
8	29	1.6	20	26	1.6
9	28	0.9	21	28	1.2
10	26	1.6	22	31	1.1
11	26	1.5	AVGE	29	1.2
12	28	1.7			

Supplementary Figure 82. The AFM images of cyclic nanostructures formed by **$Zn_9(LA)_6$**, (A) height, (B) amplitude error, (C) phase images of air-dried CH_3CN (10^{-5} M) dispersion on a silicon wafer substrate.

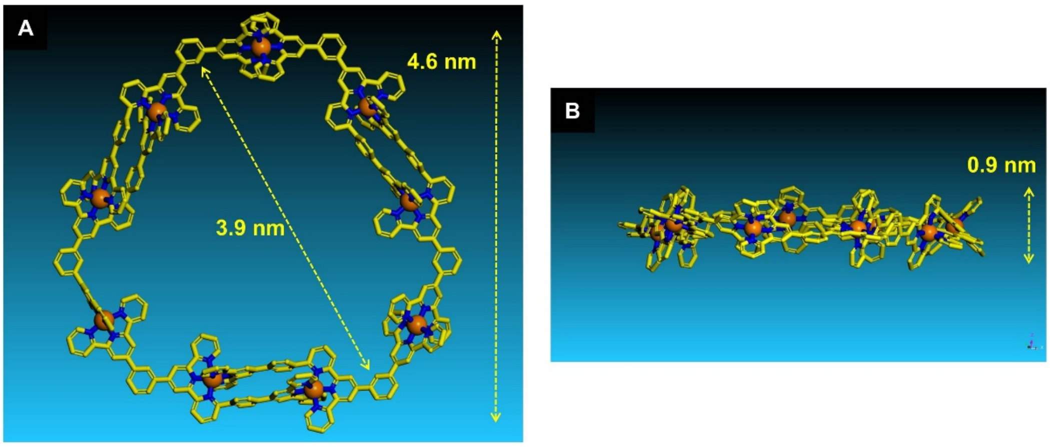

Supplementary Figure 83. Representative energy-minimized structure of **$Zn_9(LA)_6$** from molecular modeling.

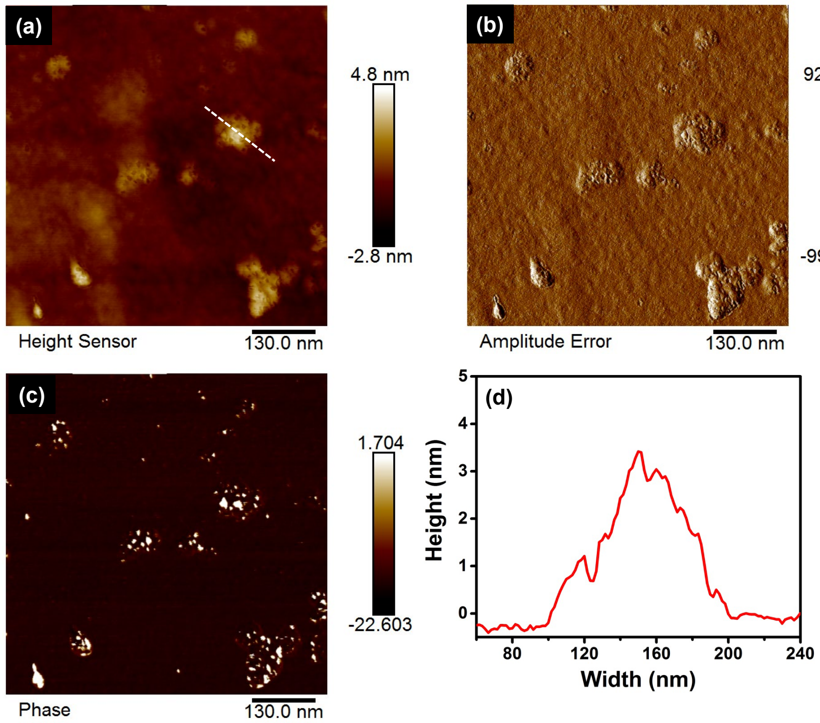

Supplementary Figure 84. The AFM images of aggregates formed by $\mathbf{Zn_{12}(LB)_6}$, (a) height, (b) amplitude error, (c) phase images of air-dried CH_3CN (10^{-5} M) dispersion on a silicon wafer substrate and (d) the cross-section of the cyclic nanostructure shown in image a.

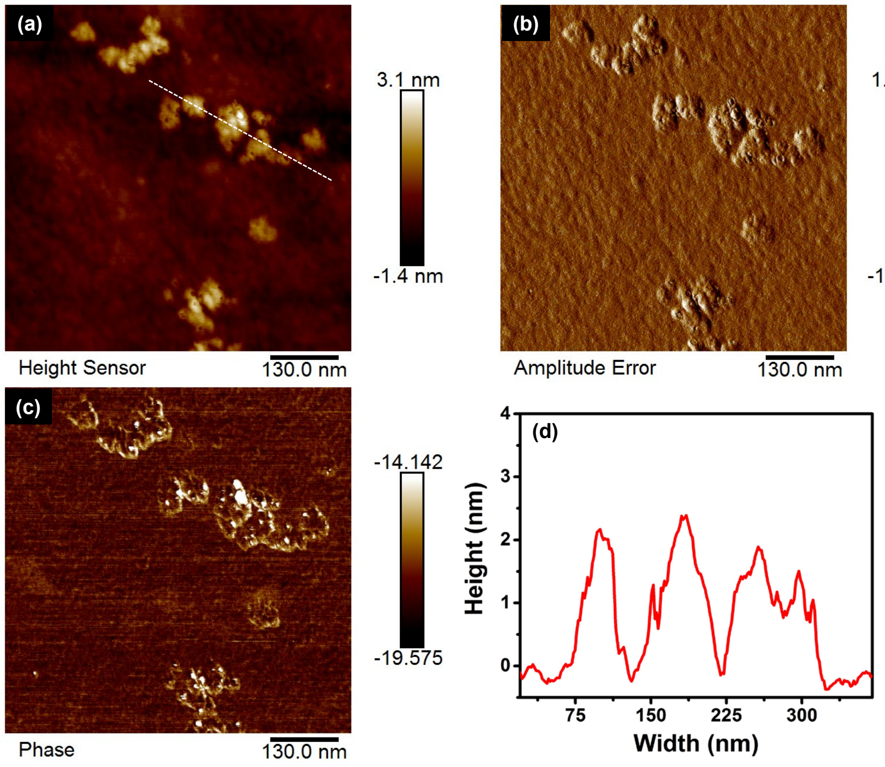

Supplementary Figure 85. The AFM images of $\mathbf{Zn_{12}(LB)_6}$, (a) height, (b) amplitude error, (c) phase images of air-dried CH_3CN (10^{-5} M) dispersion on a silicon wafer substrate. (d) the cross-section of the aggregates shown in image a.

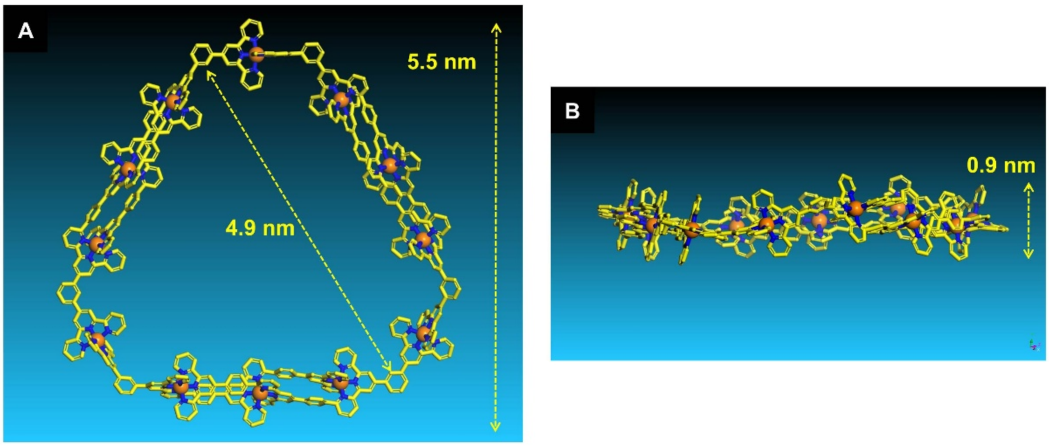

Supplementary Figure 86. Representative energy-minimized structure of **$Zn_{12}(LB)_6$** from molecular modeling.

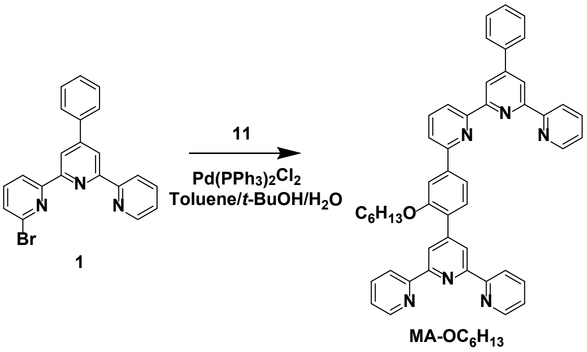

Supplementary Figure 87. Synthesis of ligand **MA-OC_6H_{13}**.

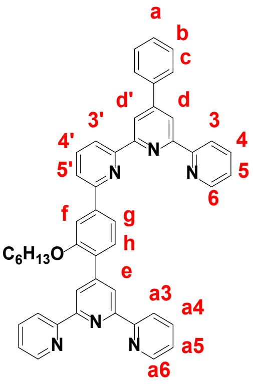

Ligand MA-OC_6H_{13}: A mixture of compound **1**[1] (775 mg, 2 mmol), compound **11** (1.61 g, 3 mmol) and $Pd(PPh_3)_2Cl_2$ (112 mg, 0.16 mmol) in 100 mL Schlenk flask was degassed three times. Then toluene (30 mL), 1 M Na_2CO_3 (15 mL) and *tert*-butyl alcohol (7 mL) were added under N_2. The resultant mixture was kept at 85 °C for 24 h. After cooling down to the room temperature, the solution was extracted three times

with $CHCl_3$, and the solvent was removed under reduced pressure. The crude product was purified by column chromatography on silica gel ($CHCl_3$/EtOH=75/1) to give compound **MA-OC$_6$H$_{13}$** as white solid (1.0 g, 72%). ^{1}H NMR (600 MHz, $CDCl_3$, 300 K) δ 8.99 (d, J = 1.7 Hz, 1H, tpy-$H^{d'}$), 8.80 (s, 2H, tpy-H^e), 8.78 (d, J = 1.7 Hz, 1H, tpy-H^d), 8.75 (ddd, J = 4.7, 1.8, 0.9 Hz, 1H, tpy-H^6), 8.74 – 8.71 (m, 3H, tpy-H^{a6} and tpy-H^3), 8.69 (dt, J = 7.9, 1.1 Hz, 2H, tpy-H^{a3}), 8.67 (dd, J = 7.8, 0.9 Hz, 1H, tpy-$H^{3'}$), 8.00 – 7.96 (m, 2H, Ph-H^f and tpy-$H^{4'}$), 7.96 – 7.93 (m, 2H, Ph-H^c), 7.89 (dtd, J = 15.6, 7.7, 1.8 Hz, 4H, tpy-H^4, tpy-$H^{5'}$ and tpy-H^{a4}), 7.83 (dd, J = 7.9, 1.6 Hz, 1H, Ph-H^g), 7.75 (d, J = 7.8 Hz, 1H, Ph-H^h), 7.54 (t, J = 7.5 Hz, 2H, Ph-H^b), 7.51 – 7.47 (m, 1H, Ph-H^a), 7.37 (ddd, J = 7.5, 4.7, 1.2 Hz, 1H, tpy-H^5), 7.34 (ddd, J = 7.5, 4.7, 1.2 Hz, 2H, tpy-H^{a5}), 4.23 (t, J = 6.3 Hz, 2H, H^{-OCH2-}), 1.47 (p, J = 7.6 Hz, 2H), 1.22 (dq, J = 8.7, 6.9 Hz, 2H), 1.18 – 1.11 (m, 2H), 0.75 (t, J = 7.3 Hz, 3H). ^{13}C NMR (150 MHz, $CDCl_3$, 300 K) δ 157.04, 156.77, 156.50, 156.24, 156.09, 155.94, 155.35, 150.28, 149.29, 148.22, 141.16, 138.88, 137.84, 137.03, 136.86, 131.09, 129.18, 129.16, 127.40, 123.97, 123.69, 121.97, 121.56, 121.38, 120.64, 120.01, 119.36, 119.31, 119.11, 111.07, 68.80, 31.74, 29.42, 26.02, 22.55, 14.10. MALDI-TOF MS (*m/z*): Calcd. for $[C_{48}H_{40}N_6O+H]^+$ 717.33. Found: 717.33.

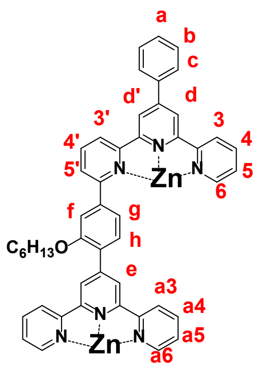

Complex Zn$_2$(MA-OC$_6$H$_{13}$)$_2$: To a solution of **MA-OC$_6$H$_{13}$** (4.8 mg, 6.7 μmol) in $CHCl_3$ (1.0 mL), a solution of $Zn(NO_3)_2$•$6H_2O$ (2.0 mg, 6.7 μmol) in MeOH (3.0 mL) was added, then the mixture was kept in a 50 °C for 12 h. After cooling to room temperature, excess NH_4PF_6 (around 80 mg) was added to generate a white precipitate (6.7 mg, 93%). ^{1}H NMR (600 MHz, CD_3CN, 300 K) δ 9.17 (d, J = 1.4 Hz, 1H, tpy-$H^{d'}$), 9.00 – 8.85 (m, 3H, tpy-H^d, tpy-$H^{3'}$ and tpy-$H^{e'}$), 8.77 – 8.51 (m, 4H, tpy-H^e,

tpy-H^{3} and tpy-H^{a3}), 8.43 (dd, J = 8.2, 7.5 Hz, 2H, tpy-$H^{4'}$ and tpy-$H^{a4'}$), 8.33 – 8.19 (m, 3H, Ph-H^{c} and tpy-H^{a4}), 8.11 – 7.97 (m, 2H, tpy-H^{4} and tpy-$H^{a6'}$), 7.92 – 7.75 (m, 4H, tpy-H^{a6}, Ph-H^{b} and Ph-H^{a}), 7.68 (s, 1H, tpy-$H^{5'}$), 7.56 (d, J = 52.8 Hz, 2H, tpy-H^{a5}), 7.36 – 7.16 (m, 3H, Ph-H^{g}, tpy-H^{6} and tpy-H^{5}), 6.35 (s, 2H, Ph-H^{f} and Ph-H^{h}), 3.54 (qd, J = 7.0, 5.3 Hz, 2H, H^{OCH2-}), 1.85 (s, 2H), 1.41 (d, J = 7.1 Hz, 2H), 1.25 (s, 2H), 0.67 (d, J = 68.3 Hz, 3H). ^{13}C NMR (150 MHz, CD_3CN, 300 K) δ 159.73, 156.46, 155.43, 151.00, 150.24, 149.28, 148.93, 148.31, 147.72, 147.09, 146.78, 141.80, 141.73, 141.42, 140.89, 136.00, 131.36, 130.44, 129.73, 128.29, 128.17, 127.93, 127.37, 125.13, 123.62, 123.17, 123.08, 122.87, 122.12, 121.89, 120.39, 112.20, 69.27, 31.28, 28.60, 25.62, 22.28. ESI-MS (*m/z*): 927.0 $[M\text{-}2PF_6^-]^{2+}$ (calcd *m/z*: 927.0), 569.7 $[M\text{-}3PF_6^-]^{3+}$ (calcd *m/z*: 569.7), 391.1 $[M\text{-}4PF_6^-]^{4+}$ (calcd *m/z*: 391.1).

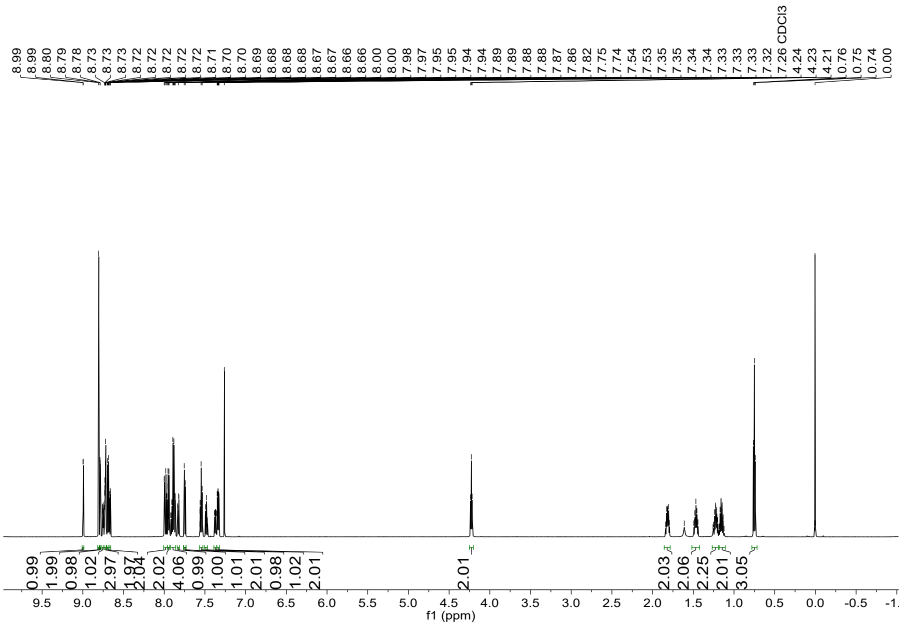

Supplementary Figure 88. ^{1}H NMR (600 MHz, $CDCl_3$, 300 K) spectrum of ligand **MA-OC$_6$H$_{13}$**.

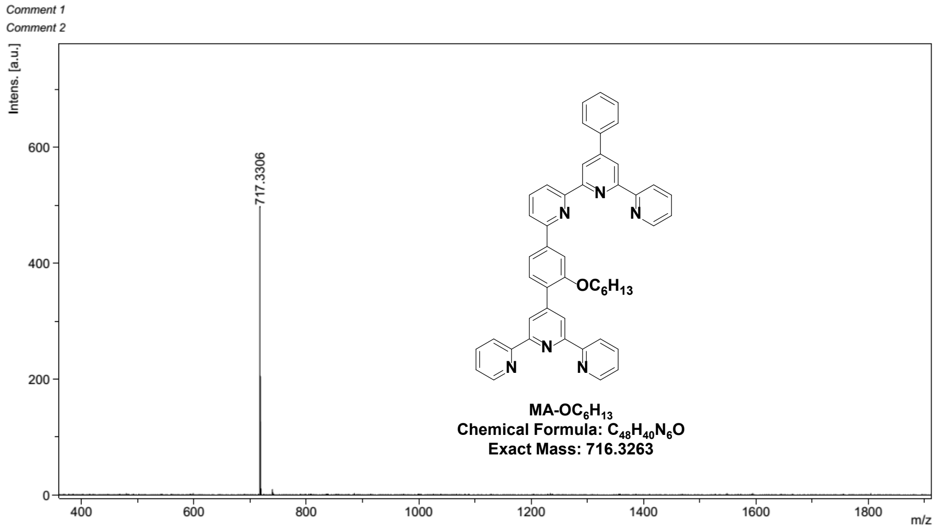

Supplementary Figure 89. MALDI-TOF plot of ligand **MA-OC$_6$H$_{13}$**.

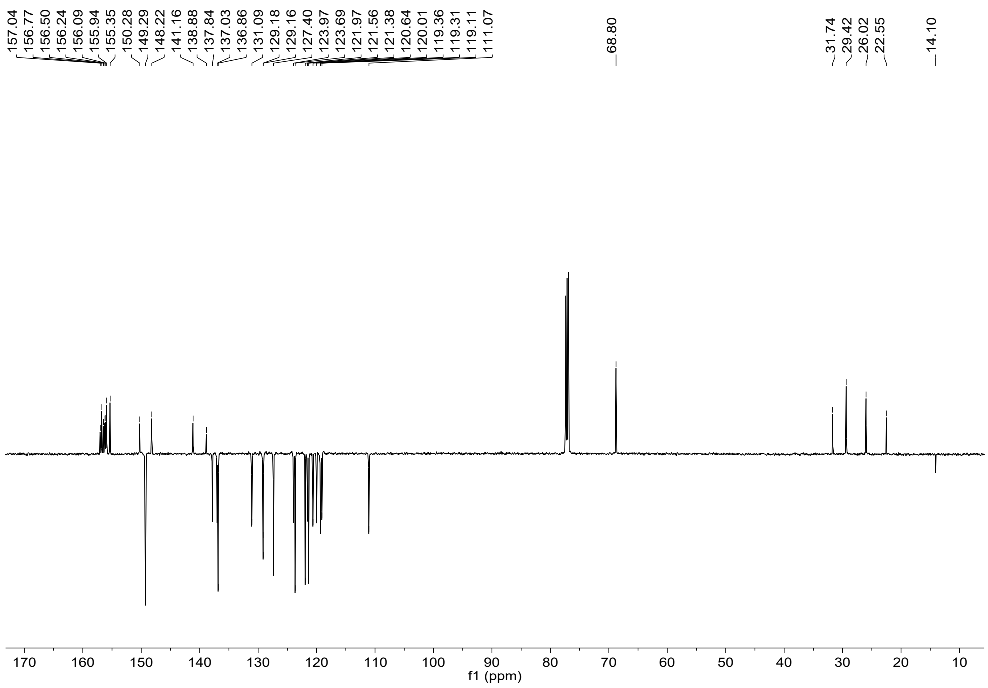

Supplementary Figure 90. DEPTQ ^{13}C NMR (150 MHz, CDCl$_3$, 300 K) spectrum of ligand **MA-OC$_6$H$_{13}$**.

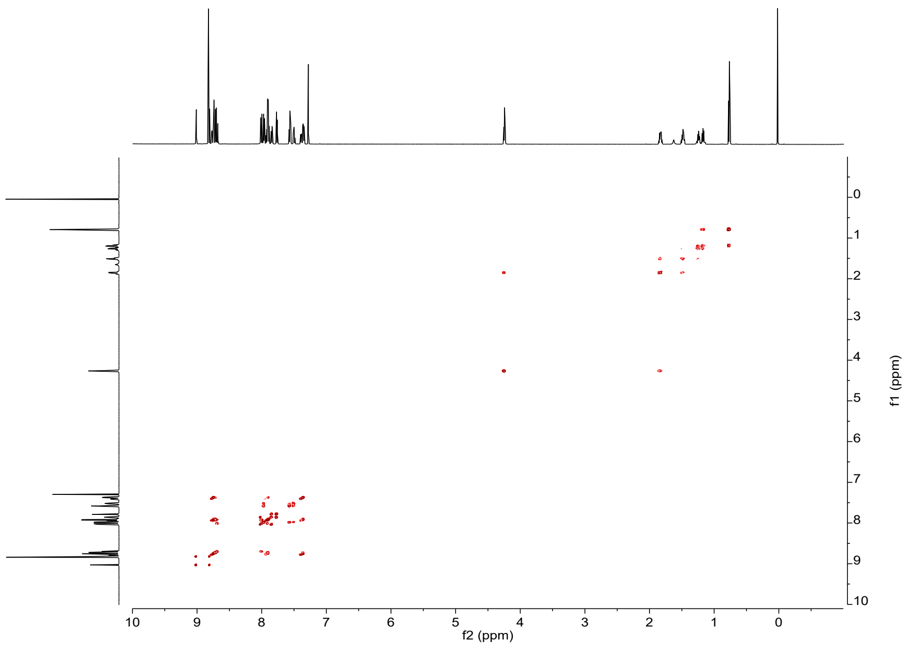

Supplementary Figure 91. 2D COSY NMR (600 MHz, $CDCl_3$, 300 K) spectrum of ligand **MA-OC$_6$H$_{13}$**.

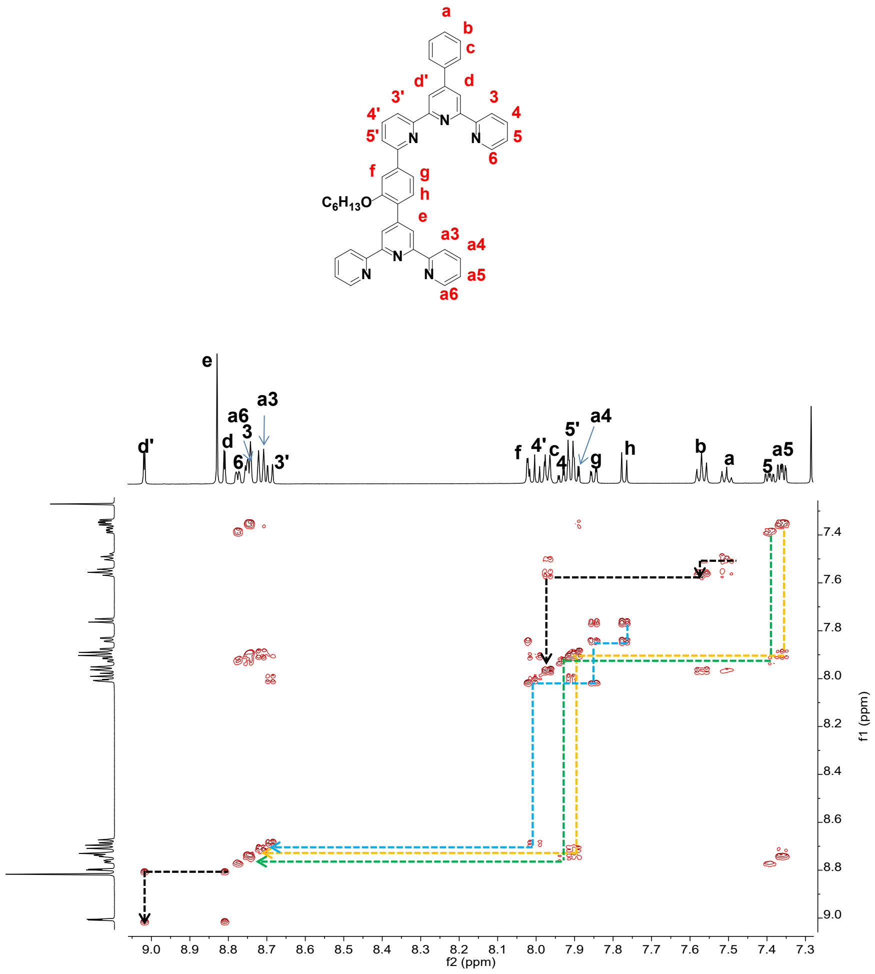

Supplementary Figure 92. 2D COSY NMR (600 MHz, $CDCl_3$, 300 K) spectrum of ligand **MA-OC$_6$H$_{13}$** (aromatic region).

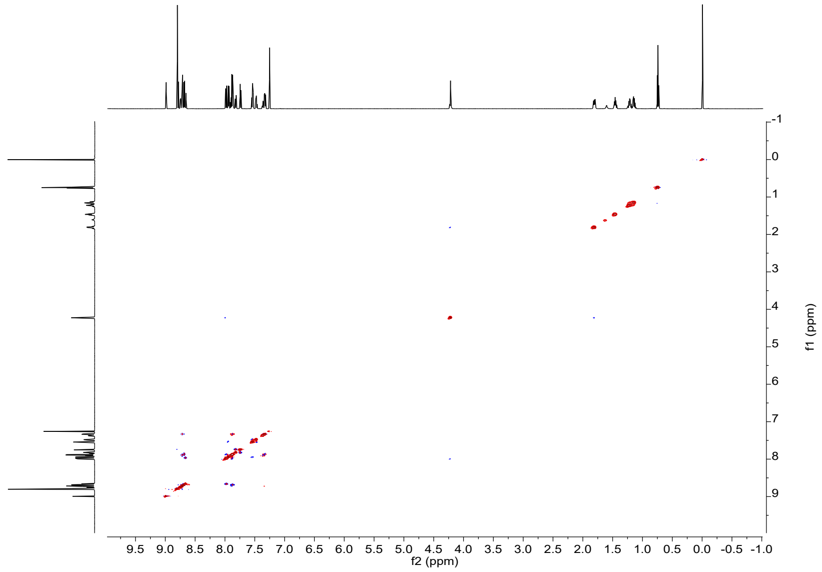

Supplementary Figure 93. 2D NOESY NMR (600 MHz, $CDCl_3$, 300 K) spectrum of ligand **MA-OC$_6$H$_{13}$**.

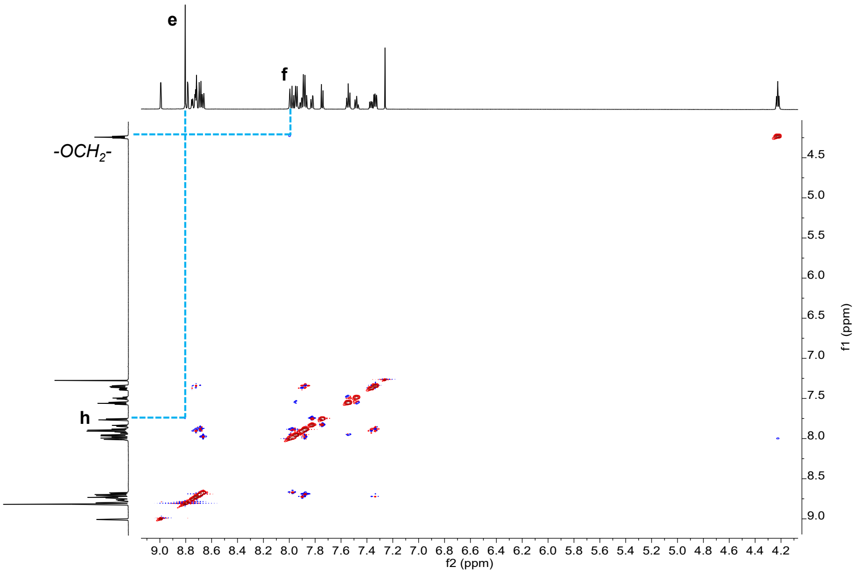

Supplementary Figure 94. 2D NOESY NMR (600 MHz, $CDCl_3$, 300 K) spectrum of ligand **MA-OC$_6$H$_{13}$**.

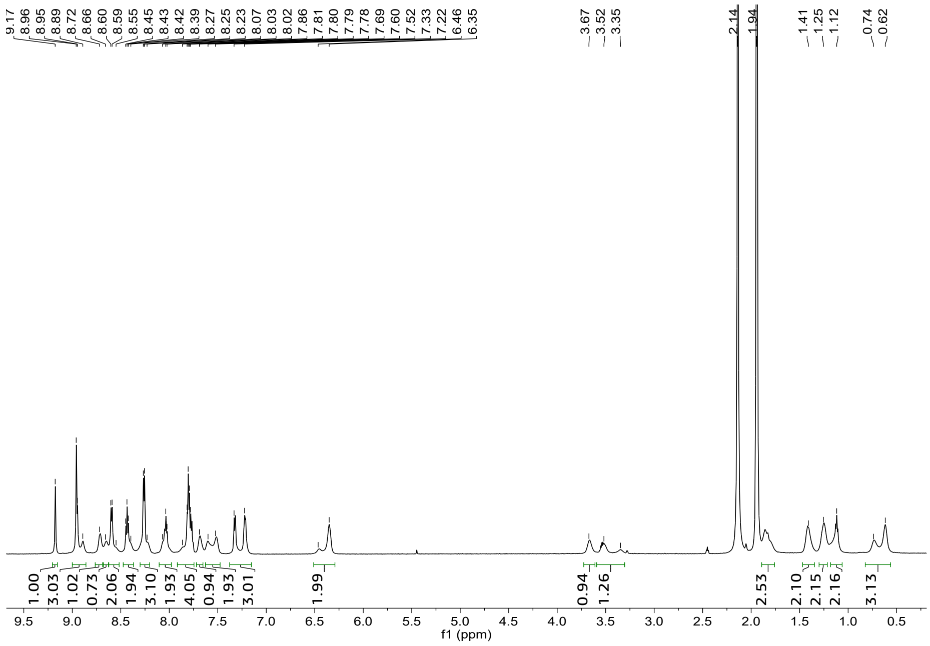

Supplementary Figure 95. ^{1}H NMR (600 MHz, CD_3CN, 300 K) spectrum of complex **Zn$_2$(MA-OC$_6$H$_{13}$)$_2$**.

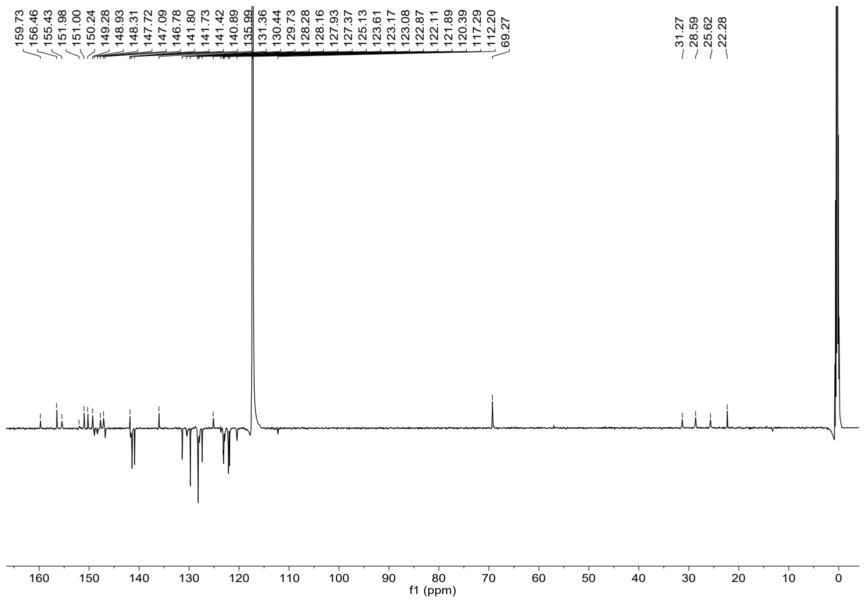

Supplementary Figure 96. DEPTQ ^{13}C NMR (150 MHz, CD_3CN, 300 K) spectrum of complex $\mathbf{Zn_2(MA\text{-}OC_6H_{13})_2}$.

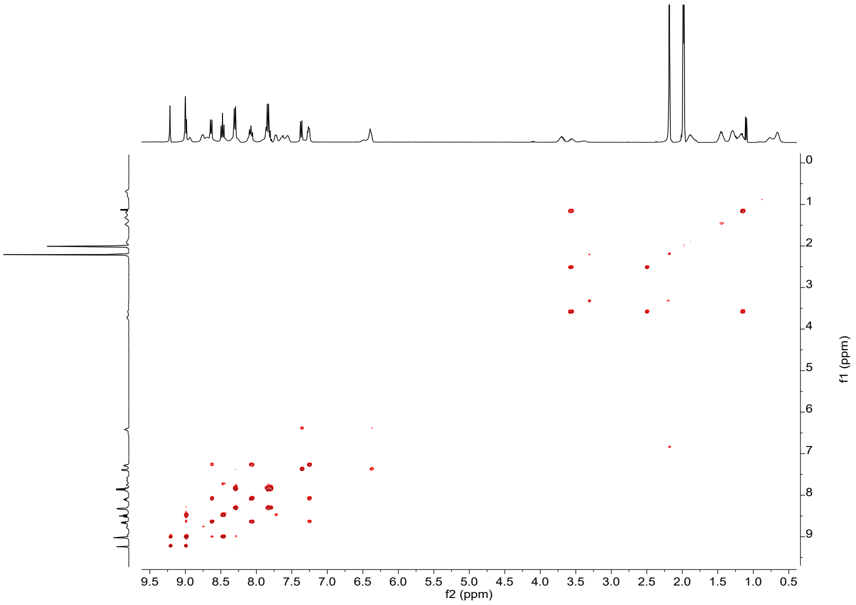

Supplementary Figure 97. 2D COSY NMR (600 MHz, CD_3CN, 300 K) spectrum of complex $\mathbf{Zn_2(MA\text{-}OC_6H_{13})_2}$.

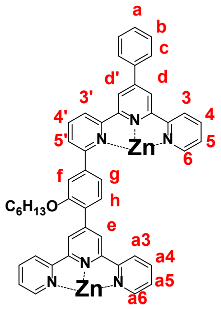

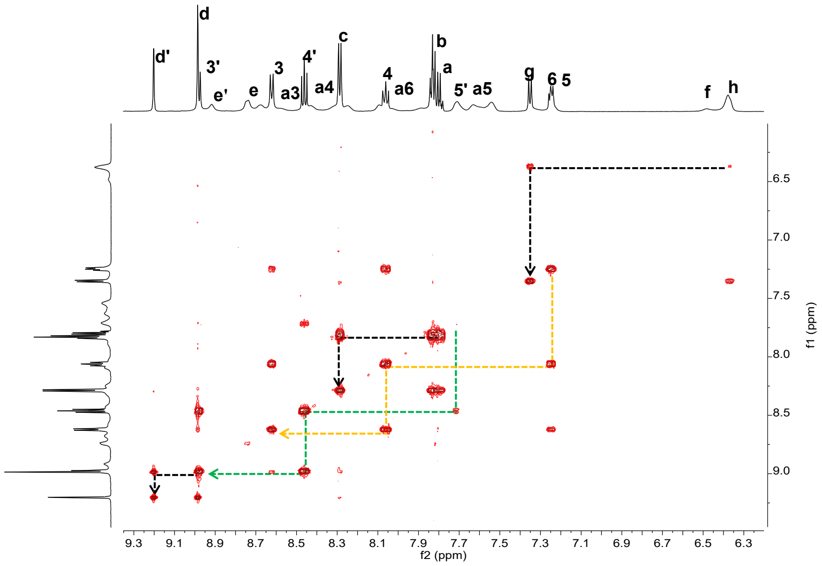

Supplementary Figure 98. 2D COSY NMR (600 MHz, CD_3CN, 300 K) spectrum of ligand **$Zn_2(MA-OC_6H_{13})_2$** (aromatic region).

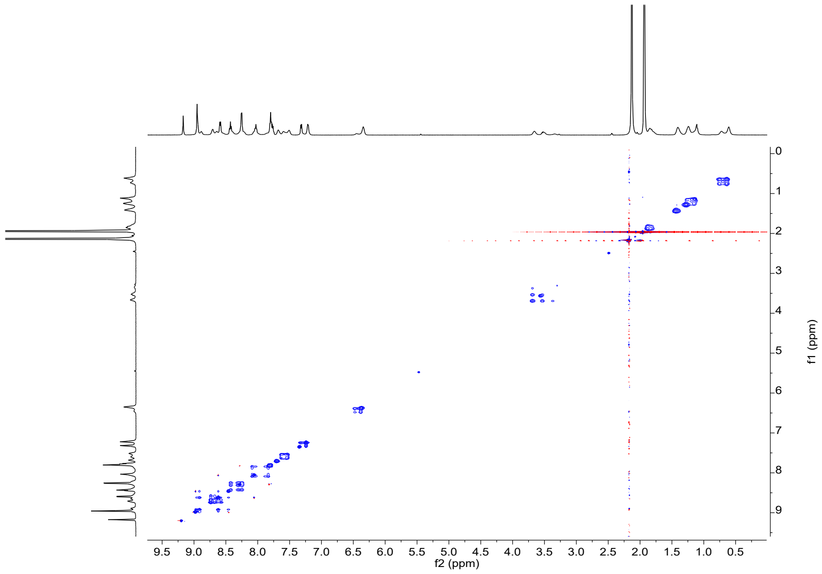

Supplementary Figure 99. 2D NOESY NMR (600 MHz, CD_3CN, 300 K) spectrum of complex **$Zn_2(MA-OC_6H_{13})_2$**.

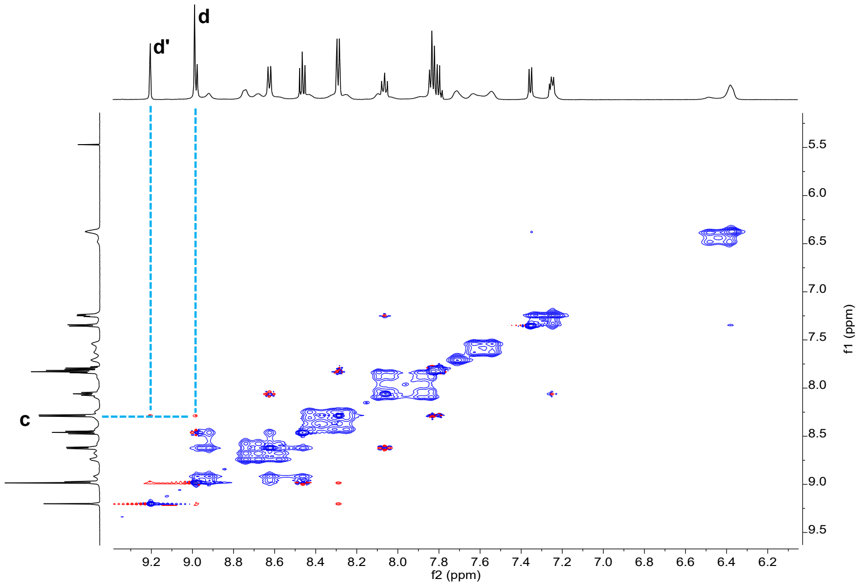

Supplementary Figure 100. 2D NOESY NMR (600 MHz, CD_3CN, 300 K) spectrum of ligand **$Zn_2(MA\text{-}OC_6H_{13})_2$** (aromatic region).

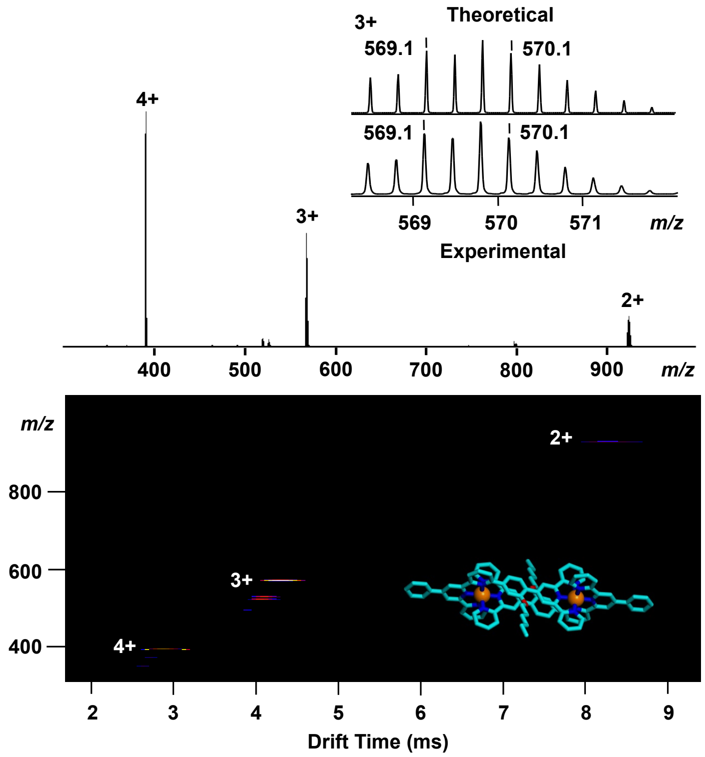

Supplementary Figure 101. (A) ESI-MS and (B) TWIM-MS plots (*m/z* vs drift time) of **$Zn_2(MA\text{-}OC_6H_{13})_2$**.

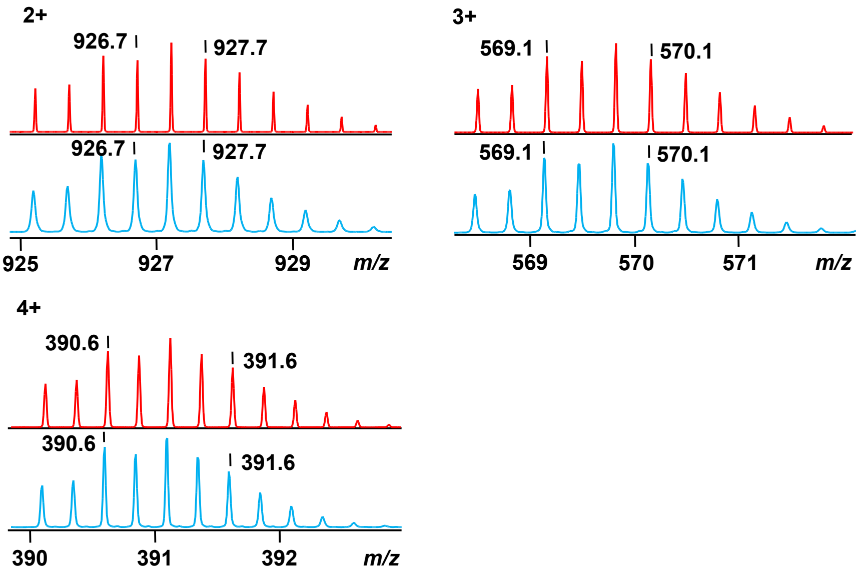

Supplementary Figure 102. Measured (blue) and calculated (red) isotope patterns for different charge states observed from **Zn_2(MA-OC_6H_{13})$_2$** (PF_6^- as counterion).

Supplementary Table 3 Crystal data and structure refinement for **Zn_2(MA-OC_6H_{13})$_2$**.

Identification code	**Zn_2(MA-OC_6H_{13})$_2$**
Empirical formula	$C_{107}H_{94}F_{24}N_{14}O_2P_4Zn_2$
Formula weight	2318.58
Temperature/K	100.0
Crystal system	monoclinic
Space group	$P2_1/c$
a/Å	19.651(7)
b/Å	36.151(8)
c/Å	15.255(5)
α/°	90
β/°	104.195(10)
γ/°	90
Volume/Å^3	10507(5)
Z	4
ρ_{calc}g/cm^3	1.466
μ/mm^{-1}	0.618
F(000)	4744.0
Crystal size/mm^3	0.12 × 0.11 × 0.1
Radiation	MoKα (λ = 0.71076)
2Θ range for data collection/°	4.834 to 53.536
Index ranges	$-24 \le h \le 24$, $-45 \le k \le 45$, $-19 \le l \le 19$
Reflections collected	293490
Independent reflections	22321 [R_{int} = 0.0860, R_{sigma} = 0.0341]
Data/restraints/parameters	22321/2053/1490

Goodness-of-fit on F^2	1.123
Final R indexes [I>=2σ (I)]	R_1 = 0.0698, wR_2 = 0.1738
Final R indexes [all data]	R_1 = 0.0848, wR_2 = 0.1823
Largest diff. peak/hole / e Å^{-3}	1.53/-1.13

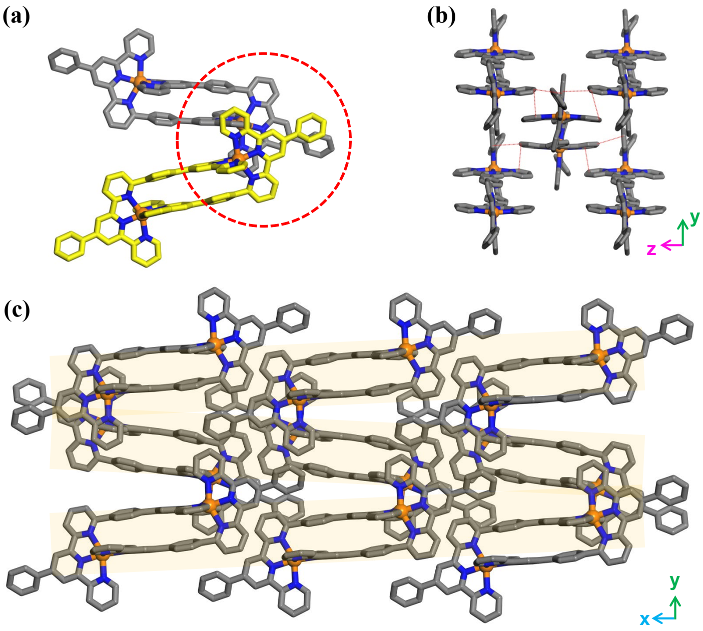

Supplementary Figure 103. (a) Crystal packing of complex **$Zn_2(MA)_2$** and (b), (c) 2D stacking diagram of **$Zn_2(MA)_2$**.

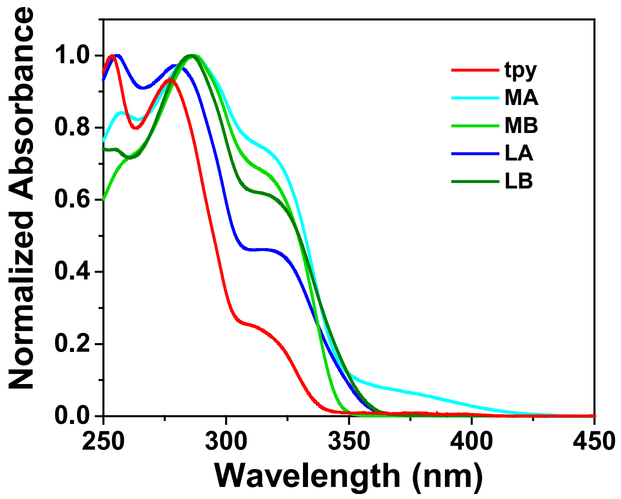

Supplementary Figure 104. UV/Vis absorption spectra of the ligands in $CHCl_3$ (10^{-5} M).

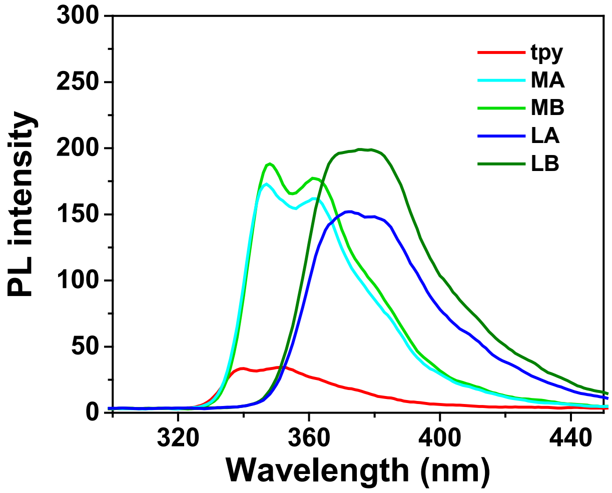

Supplementary Figure 105. PL spectra of the ligands in $CHCl_3$ (10^{-6} M, λ_e = 280 nm).

Supplementary References

1. Schnaubelt, L.; Petzold, H.; Hörner, G.; Rüffer, T.; Klein, N.; Lang, H., *Eur. J. Inorg. Chem.* **2019**, 2019, 988-1001.
2. Zhang, G.; Zhu, C.; Liu, D.; Pan, J.; Zhang, J.; Hu, D.; Song, B., *Tetrahedron* **2017**, *73*, 129-136.
3. Song, G.; Liu, R.; He, G.; Yuan, S.; Zhu, H., *Asian J. Org. Chem.* **2015**, *4*, 346-353.
4. KröHnke, F., *Synthesis* **1976**, 1976, 1-24.
5. Wang, M.; Wang, C.; Hao, X.-Q.; Liu, J.; Li, X.; Xu, C.; Lopez, A.; Sun, L.; Song, M.-P.; Yang, H.-B.; Li, X., *J. Am. Chem. Soc.* **2014**, *136*, 6664-6671.
